# Supplementary material for: Effectiveness of the Strengthening Families Programme in the UK at preventing substance misuse in 10–14 year-olds: a pragmatic randomised controlled trial
Source: BMJ Open. 2022 Feb 21;12(2):e049647. doi: 10.1136/bmjopen-2021-049647 (PMC8862464; doi:10.1136/bmjopen-2021-049647)
Supplement: Supplementary data [file bmjopen-2021-049647supp001.pdf]

Supplementary material

Table S1: Measures collected at 24 month follow-up from young people

Domain/question topic	Purpose	Measure/Source
**Behaviour - Strengths and Difficulties**		
Strengths and difficulties / wellbeing and stress	TO	SDQ[1]
**Life at Home**		
Family activities (opportunities for involvement in pro-social activities; possibly bonding)	ST	From HBSC/ PEACH study
Young people's own time		From West of Scotland Twenty 07
Parenting/child management	IO/ TO	General Child Management Measure, Project Family[2-4]
Parents and school		Three questions; one adapted from the Child Rearing Practices Measure
Help around the home	IO	Adapted versions of questions asked to parents at 9/15 months.
Development of self-efficacy	ST/ TO	Bandura's Self Efficacy Scale[5]
Attachment to parents	ST	The Security Scale[6]
Befriending pro-social/anti-social peers	ST	Social Development Model scale on friends' anti-social qualities (Interaction with antisocial peers scale)[7]
Positive bonding to school	ST	School Bonding Measure / SDM[8]
**Participant substance use**		
Smoking behaviour – ever smoked?		Question from Substance Initiation Index[9]
Smoking status	TO/ SO	ASSIST study version of NatCen/NFER question[10]
Age first smoked	TO	From HBSC questionnaire
Ever drunk a drink?		NatCen/NFER[11]
Age of first drink	SO	Adapted from HBSC/NatCen/NFER
Drinking frequency, last month (including different types)	SO	Adapted questions from HBSC questionnaire
Ever really drunk?		From HBSC questionnaire
Age first drunk		Adapted version of question in HBSC questionnaire
Number of times drunk, drinking alcohol or 5+ drinks in a row in last month	PO/SO	Amended from HBSC questionnaire
Alcohol-related problems	SO	Q21 from ESPAD survey[12]
Drug use – ever used?		Amended from HBSC/NatCen/NFER[11]
Cannabis use (ever, 12 months, or 30 days)	SO	From HBSC questionnaire
Age of first drug use	TO	Adapted from HBSC questionnaire
**General health**		
Health state today	HE/TO	EQ-5D (child version)[13]
General health	HE/TO	UK Kidscreen 10[14]

Table S2: Measures collected at 24 month follow-up from parents/carers

Domain/question topic	Purpose	Measure/Source
**Life at home**		
Family activities		From HBSC/11-16 West of Scotland adult questionnaire
Family functioning	TO	Family relationship index[15,16]
Parenting/child management	SO, IO	General child management measure, Project Family[2-4]
Parent–child bonding	ST	Adapted from Spoth's rural urban cumulative risk index and Arthur, et al.[17,18]
Parents and school		Question developed by project team and one from Conger's child rearing practices measure
**Participant substance use**		
Smoking behaviour		Heaviness of smoking index[19]
Alcohol use		Adapted AUDIT-C questions, as used by Pre-empt study[20]
Drug use		Adapted from HBSC/NatCen/ NFER[11]
**Health**		
Health status	HE, TO	GHQ[21,22]
Health	HE	1 item from SF-36[23]
Health state today	HE, TO	EQ-5D[13]
**Service utilisation**		
Health, social care, education and criminal justice service utilisation	HE	Modified from ALSPAC

Secondary analyses of the primary outcome

After adjusting alcohol use outcome for gender of young people, time from randomisation to 24 month follow-up, and intervention receipt, the results from multilevel modelling indicated that none of the adjustments had altered the results drawn from the primary analysis (Table 8). Although the alcohol use outcome measure had been restructured into an ordinary outcome measure, the results from the ordinal regression model provided a corresponded proportional odds ratio of 1.09 (0.70-1.71) with adjustments and proportional odds ratio of 1.11 (0.73-1.67) without adjustments, which suggests that there was no evidence of a between-group difference.

Table S3: Secondary analyses of the primary alcohol use outcome

Analysis	Odds ratio	95% Confidence Interval		p-value
		Lower limit	Upper limit	
Without any adjustments	1.11	0.74	1.66	0.626
Adjusting for gender of young person	1.11	0.72	1.72	0.641
Adjusting for time from randomisation to 24-month follow-up	1.08	0.70	1.68	0.725
Adjusting for intervention receipt (binary definition)	1.21	0.79	1.86	0.384
Adjusting for intervention receipt (number of weeks)	1.03	0.97	1.09	0.382
Analysis	Proportional odds ratio	95% Confidence Interval		p-value
		Lower limit	Upper limit	
As an ordinal outcome (adjusted)	1.09	0.70	1.71	0.689
As an ordinal outcome (without any adjustments)	1.11	0.73	1.67	0.637

In terms of drunkenness in the previous 30 days, results show that the model without controlling any adjustments reduced the treatment effect odds ratio from 1.46 to 1.27 but did not alter the conclusions that could be drawn from the analysis (Table 9). Similarly, when the model was adjusted for gender for young person, time from randomisation to 24 month follow-up, and intervention receipt did not alter the conclusions, which suggests that there was no evidence of a between-group difference.

Table S4: Secondary analyses of the primary drunkenness outcome

Analysis	Odds ratio	95% Confidence Interval		p-value
		Lower limit	Upper limit	
Without any adjustments	1.27	0.73	2.21	0.392
Adjusting for gender of young person	1.48	0.84	2.58	0.173
Adjusting for time from randomisation to 24-month follow-up	1.35	0.77	2.37	0.298
Adjusting for intervention receipt (binary definition)	1.50	0.80	2.79	0.203
Adjusting for intervention receipt (number of weeks)	1.06	0.97	1.16	0.196
Analysis	Proportional odds ratio	95% Confidence Interval		p-value
		Lower limit	Upper limit	
As an ordinal outcome (adjusted)	1.42	0.82	2.46	0.211
As an ordinal outcome (without any adjustments)	1.26	0.71	2.25	0.429

Multiple imputation for primary outcomes

We randomised 931 young people within 715 families in total. For our two primary outcomes, this represents our full ITT population. We conducted sensitivity analysis in order to estimate the full ITT effect.

We had two participants who did not respond to the baseline alcohol questions and assumed that neither had consumed alcohol within the past 30 days when asked at baseline.

We imputed primary outcomes using a single-level imputation model which contained variables included in the analysis model and whether the young person had ever smoked (as reported at baseline). We imputed 20 datasets and used augmented regression to avoid perfect prediction.

In the table below, we present estimates from the primary analyses (which adjusts for multiple young people within families), estimates of a single-level version of the primary analyses, and single-level imputed estimates.

Table S5: Primary analyses accounting for missing data using multiple imputation

Outcome	Model	OR	95% CI	p-value
Alcohol use at 24-months	Primary	1.11	0.72 to 1.71	0.646
	Single-level primary	1.11	0.77 to 1.61	0.574
	Single-level imputed	1.11	0.75 to 1.63	0.612
Drunkenness at 24-months	Primary	1.46	0.83 to 2.55	0.185
	Single-level primary	1.46	0.83 to 2.55	0.185
	Single-level imputed	1.48	0.85 to 2.56	0.162

Primary outcomes – subgroup analysis

An association was found between age of young person at baseline and reported alcohol use at 24-month follow-up (1.82 [1.23-2.71]), but there was no evidence of any differential effect by trial arms (1.30 [0.85-1.98]). In terms of gender of young person, the odds ratios for main effects and subgroup interaction terms suggested that the intervention worked differently for males and females. However, both confidence intervals included one, which suggests that there was no evidence of a differential treatment effect by gender of young person. Additionally, the main effect of a young person reporting smoking at baseline (2.08 [1.06-4.11]) and having at least one parent/carer categorised at high-risk from problematic drinking (1.93 [1.01-3.69]) were significantly associated with the alcohol use outcome, but there were no discernible differences between arms. Other planned subgroup analyses provided no evidence of the SFP group having a differential effect.

An association was found between a young person reporting smoking at baseline and reported drunkenness at 24-month follow-up (5.73 [2.07-15.90]), but there was no evidence of any differential effect by trial arms (0.57 [0.17-1.94]). In terms of the average young person GCM score (General Child Management), a one-point increase on the GCM score was associated with a 59% decrease in the odds of being classed as a young person who had been drunk in the last 30 days (0.41 [0.18-0.96]) a (higher GCM score indicates greater levels of parent management). However, there was no evidence of any differential effect by trial arm (1.27 [0.45-3.56]). For gender of young person, the odds ratio for main effect showed a 17% increase for females who reported having been drunk in the previous 30 days at 24-month follow up compared to males, and the subgroup interaction indicated an 8% increase for females allocated to the SFP group compared to males and those allocated to the control group. However, both confidence intervals included one, which suggests that there was no evidence of a differential treatment effect by gender of young person. Overall, none of the subgroup analyses for the primary drunkenness outcome proved a significant association, which suggests that there was no evidence of a differential treatment effect.

Table S6: Subgroup analyses for the primary alcohol use outcome

Subgroup	Main effect odds ratio	95% Confidence Interval Lower limit	95% Confidence Interval Upper limit	p-value	Subgroup x SFP interaction	95% Confidence Interval Lower limit	95% Confidence Interval Upper limit	p-value
Age of young person at baseline	1.82	1.23	2.71	0.003	1.30	0.85	1.98	0.228
Gender of young person (female)	0.85	0.44	1.57	0.574	1.48	0.65	3.57	0.337
Young person had smoked at baseline	2.08	1.06	4.11	0.034	0.79	0.32	1.91	0.596
Having at least one parent/carer who is at high risk from problematic drinking	1.93	1.01	3.69	0.048	0.79	0.33	1.87	0.593
Baseline Family Affluence Scale (Medium)	1.13	0.44	2.86	0.750	1.08	0.31	3.73	0.589
Baseline Family Affluence Scale (High)	1.09	0.39	3.00		1.75	0.46	6.70	
Occupation Status (At least one parent in the family in part-time (but none in full-time) employment)	0.60	0.24	1.53	0.448	0.86	0.24	3.09	0.720
At least one parent in the family in full-time employment	0.91	0.41	2.02		1.46	0.50	4.25	
Young person belonging to a “family with challenges”	0.99	0.52	1.91	0.984	0.51	0.21	1.24	0.140
Strengths and difficulties score at baseline	1.02	0.97	1.07	0.498	1.00	0.93	1.07	0.929
Cohesion domain of the Family Relationship Index at baseline	1.01	0.99	1.03	0.328	0.99	0.96	1.01	0.273
Expressiveness domain of the Family Relationship Index at baseline	1.01	0.99	1.04	0.339	0.99	0.96	1.03	0.760
Conflict domain of the Family Relationship Index at baseline	1.02	0.99	1.05	0.211	1.00	0.97	1.04	0.871
Average Young person General Child Management score	1.01	0.54	1.91	0.968	0.45	0.18	1.12	0.086
Average Parent/carer General Child Management score	1.54	0.76	3.15	0.233	0.67	0.28	1.64	0.383

Table S7: Subgroup analyses for the primary drunkenness outcome

Subgroup	Main effect odds ratio	95% Confidence Interval		p-value	Subgroup x SFP interaction	95% Confidence Interval		p-value
		Lower limit	Upper limit			Lower limit	Upper limit	
Age of young person at baseline	2.22	0.98	5.04	0.056	1.30	0.67	2.53	0.435
Gender of young person (female)	1.17	0.49	2.80	0.690	1.08	0.35	3.29	0.920
Young person had smoked at baseline	5.73	2.07	15.90	0.001	0.57	0.17	1.94	0.369
Having at least one parent/carer who is at high risk from problematic drinking	1.40	0.58	3.38	0.448	1.68	0.53	5.26	0.376
Baseline Family Affluence Scale (Medium)	1.26	0.36	4.43	0.336	2.57	0.46	14.45	0.563
Baseline Family Affluence Scale (High)	0.99	0.25	3.88		2.18	0.34	14.15	
Occupation Status (At least one parent in the family in part-time (but none in full-time) employment)	1.31	0.46	3.73	0.673	0.76	0.17	3.33	0.550
At least one parent in the family in full-time employment	0.76	0.25	2.30		1.87	0.47	7.43	
Young person belonging to a "family with challenges"	1.19	0.50	2.85	0.692	0.41	0.13	1.30	0.131
Strengths and difficulties score at baseline	0.97	0.90	1.04	0.366	1.03	0.94	1.13	0.570
Cohesion domain of the Family Relationship Index at baseline	1.01	0.98	1.03	0.578	0.99	0.96	1.03	0.730
Expressiveness domain of the Family Relationship Index at baseline	0.99	0.96	1.02	0.516	1.01	0.97	1.05	0.661
Conflict domain of the Family Relationship Index at baseline	1.00	0.97	1.04	0.791	0.99	0.94	1.03	0.584
Average Young person General Child Management score	0.41	0.18	0.96	0.040	1.27	0.45	3.56	0.649
Average Parent/carer General Child Management score	1.13	0.43	2.98	0.804	0.77	0.24	2.46	0.656

Weekly smoking validated by salivary cotinine measures

Overall, 500 young people (53.7% of those randomised, 66.1% of those who provided 24-month follow-up interview data) provided saliva cotinine samples and self-reported weekly smoking behaviour at the 24-month follow-up interview. To identify weekly smokers using saliva cotinine, a cut-off of 15ng/mL was used[10,24]. Comparing cotinine samples with self-reported weekly smoking, 467 young people provided self-reported smoking data that agreed with their cotinine samples (93.4%). A slightly higher percentage of young people in

the SFP group reported being a non-weekly smoker and had cotinine samples ≤ 15 ng/mL and less than those who reported that they were a weekly smoker but had cotinine levels of ≤ 15 ng/mL.

Table S8: Agreement between self-reported weekly smoking behaviour and saliva cotinine

Self-report and saliva cotinine agreement		Control		SFP		Total	
		n	%	n	%	n	%
Concordant	Agree non-weekly smoker	176	78.6	227	82.2	403	80.6
	Agree weekly smoker	30	13.4	34	12.3	64	12.8
Discordant	Self-reported non-weekly smoker and cotinine > 15 ng/mL	13	5.8	9	3.3	22	4.4
	Self-reported weekly smoker and cotinine ≤ 15 ng/mL	5	2.2	6	2.2	11	2.2
Total		224	100.0	276	100.0	500	100.0

References

1. Goodman R. The Strengths and Difficulties Questionnaire: A Research Note. *Journal of Child Psychology and Psychiatry* 1997; **38**(5): 581-6.
2. McMahon R, Metzler C. Selecting parenting measures for assessing family based prevention interventions. In: Ashery R, Robertson E, Kumpfer K, Rockville M, eds. Drug abuse prevention through family interventions: National Institute on Drug Abuse; 1998: 294-323.
3. Spoth R, Redmond C, Haggerty K, Ward T. A Controlled Parenting Skills Outcome Study Examining Individual Difference and Attendance Effects. 1995; **57**(2): 449.
4. Spoth R, Redmond C, Shin C. Direct and indirect latent-variable parenting outcomes of two universal family-focused preventive interventions: extending a public health-oriented research base. *Journal of consulting and clinical psychology* 1998; **66**(2): 385-99.
5. Bandura A. A Guide for Constructing Self-Efficacy Scales. In: Pajares F, Urdan T, Greenwich, CT, eds. Self-Efficacy Beliefs of Adolescents: Information Age Publishing; 2006: 307-37.
6. Kerns K, Aspelmeier J, Gentzler A, Grabill C. Parent–child attachment and monitoring in middle childhood. *Journal of Family Psychology* 2001; **15**: 69-81.
7. Arthur M, Hawkins J, Catalano R, Pollard J. Student Survey of Risk and Protective Factors and Prevalence of Alcohol, Tobacco, & Other Drug Use. University of Washington: Social Development Research Group.
8. Hawkins JD, Guo J, Hill KG, Battin-Pearson S, Abbott RD. Long-Term Effects of the Seattle Social Development Intervention on School Bonding Trajectories. *Applied Developmental Science* 2001; **5**(4): 225-36.
9. Spoth RL, Redmond C, Trudeau L, Shin C. Longitudinal substance initiation outcomes for a universal preventive intervention combining family and school programs. *Psychology of Addictive Behaviors* 2002; **16**(2): 129-34.
10. Campbell R, Starkey F, Holliday J, et al. An informal school-based peer-led intervention for smoking prevention in adolescence (ASSIST): a cluster randomised trial. *The Lancet* 2008; **371**(9624): 1595-602.
11. National Centre for Social Research NFfE, Research. Smoking, Drinking and Drug Use Among Young People in England

in 2008 Full Report: NHS Information Centre for Health and Social Care, 2009.
12. Hibell B, Guttormsson U, Ahlström S, et al. The 2011 ESPAD Report: Substance Use Among Students in 36 European Countries: The Swedish Council for Information on Alcohol and other Drugs (CAN), 2011.

13. Rabin R, Charro FD. EQ-SD: a measure of health status from the EuroQol Group. *Annals of Medicine* 2001; **33**(5): 337-43.
14. The Kidscreen Group Europe. The KIDSCREEN Questionnaires: Quality of life questionnaires for children and adolescents: Handbook. Lengerich: Pabst Science Publishers, 2006.
15. Holahan CJ, Moos RH. Social support and adjustment: Predictive benefits of social climate indices. 1982; **10**(4): 403-15.
16. Billings AG, Moos RH. The role of coping responses and social resources in attenuating the stress of life events. *Journal of Behavioral Medicine* 1981; **4**(2): 139-57.

17. Spoth R, Goldberg C, Neppl T, Trudeau L, Ramisetty-Mikler S. Rural–urban differences in the distribution of parent-reported risk factors for substance use among young adolescents. *Journal of Substance Abuse* 2001; **13**(4): 609-23.
18. Arthur M, Hawkins J, Catalano R, Pollard J. Item-construct dictionary for the student survey of risk and protective factors and prevalence of alcohol, tobacco, and other drug use. Unpublished Technical Document. . Seattle, WA.: University of Washington, 1995.
19. Borland R, Yong HH, O'Connor RJ, Hyland A, Thompson ME. The reliability and predictive validity of the Heaviness of Smoking Index and its two components: Findings from the International Tobacco Control Four Country study. 2010; **12**(Supplement 1): S45-S50.
20. Bush K. The AUDIT Alcohol Consumption Questions (AUDIT-C)<subtitle>An Effective Brief Screening Test for Problem Drinking</subtitle>. *Archives of Internal Medicine* 1998; **158**(16): 1789.
21. Goldberg D. The Detection of Psychiatric Illness by Questionnaire. London: Oxford University Press; 1972.
22. Vieweg BW, Hedlund JL. The General Health Questionnaire (GHQ): A comprehensive review. *Journal of Operational Psychiatry* 1983; **14**(2): 74-81.
23. Ware JE, Sherbourne CD. The MOS 36-Item Short-Form Health Survey (SF-36). *Medical Care* 1992; **30**(6): 473-83.
24. Benowitz NL, Iii PJ, Ahijevych K, et al. Biochemical verification of tobacco use and cessation. *Nicotine & Tobacco Research* 2002; **4**(2): 149-59.
