# Supplementary material for: Effectiveness of the Strengthening Families Programme in the UK at preventing substance misuse in 10–14 year-olds: a pragmatic randomised controlled trial
Source: BMJ Open. 2022 Feb 21;12(2):e049647. doi: 10.1136/bmjopen-2021-049647 (PMC8862464; doi:10.1136/bmjopen-2021-049647)
Supplement: Author's manuscript [file bmjopen-2021-049647.draft_revisions.pdf]

BMJ Open is committed to open peer review. As part of this commitment we make the peer review history of every article we publish publicly available.

When an article is published we post the peer reviewers' comments and the authors' responses online. We also post the versions of the paper that were used during peer review. These are the versions that the peer review comments apply to.

The versions of the paper that follow are the versions that were submitted during the peer review process. They are not the versions of record or the final published versions. They should not be cited or distributed as the published version of this manuscript.

BMJ Open is an open access journal and the full, final, typeset and author-corrected version of record of the manuscript is available on our site with no access controls, subscription charges or pay-per-view fees (http://bmjopen.bmj.com).

If you have any questions on BMJ Open's open peer review process please email info.bmjopen@bmj.com

BMJ Open

The effectiveness of the Strengthening Families Programme in the UK at preventing substance misuse in 10-14 year olds: a pragmatic randomised controlled trial

Journal:	*BMJ Open*
Manuscript ID	bmjopen-2021-049647
Article Type:	Original research
Date Submitted by the Author:	03-Feb-2021

Complete List of Authors:	Segrott, Jeremy; Cardiff University, Centre for Trials Research; Cardiff University, DECIPHer Centre, Cardiff School of Social Sciences<br>Gillespie, David; Cardiff University, Centre for Trials Research<br>Lau, Mandy; Cardiff University, Centre for Trials Research<br>Holliday, Jo; University of Oxford, UK Biobank, Big Data Institute<br>Murphy, Simon; Cardiff University, DECIPHer Centre, Cardiff School of Social Sciences Cardiff, UK<br>Foxcroft, David; Oxford Brookes University UK, Department of Psychology, Health and Professional Development, Faculty of Health and Life Sciences<br>Hood, Kerenza; Cardiff University, Centre for Trials Research<br>Scourfield, Jonathan; Cardiff University, School of Social Sciences<br>Phillips, Ceri; Swansea University, Human and Health Sciences<br>Roberts, Zoe; Cardiff University, Centre for Medical Education, School of Medicine<br>Rothwell, Heather; Cardiff University, DECIPHer Centre, Cardiff School of Social Sciences<br>Hurlow, Claire; Swansea University, Swansea Trials Unit, Swansea University Medical School<br>Moore, L; University of Glasgow, MRC/CSO Social and Public Health Sciences Unit

Keywords:	PUBLIC HEALTH, Substance misuse < PSYCHIATRY, Community child health < PAEDIATRICS

SCHOLARONE™ Manuscripts

I, the Submitting Author has the right to grant and does grant on behalf of all authors of the Work (as defined in the below author licence), an exclusive licence and/or a non-exclusive licence for contributions from authors who are: i) UK Crown employees; ii) where BMJ has agreed a CC-BY licence shall apply, and/or iii) in accordance with the terms applicable for US Federal Government officers or employees acting as part of their official duties; on a worldwide, perpetual, irrevocable, royalty-free basis to BMJ Publishing Group Ltd ("BMJ") its licensees and where the relevant Journal is co-owned by BMJ to the co-owners of the Journal, to publish the Work in this journal and any other BMJ products and to exploit all rights, as set out in our licence.

The Submitting Author accepts and understands that any supply made under these terms is made by BMJ to the Submitting Author unless you are acting as an employee on behalf of your employer or a postgraduate student of an affiliated institution which is paying any applicable article publishing charge ("APC") for Open Access articles. Where the Submitting Author wishes to make the Work available on an Open Access basis (and intends to pay the relevant APC), the terms of reuse of such Open Access shall be governed by a Creative Commons licence – details of these licences and which Creative Commons licence will apply to this Work are set out in our licence referred to above.

Other than as permitted in any relevant BMJ Author's Self Archiving Policies, I confirm this Work has not been accepted for publication elsewhere, is not being considered for publication elsewhere and does not duplicate material already published. I confirm all authors consent to publication of this Work and authorise the granting of this licence.

For peer review only

The effectiveness of the Strengthening Families Programme in the UK at preventing substance misuse in 10-14 year olds: a pragmatic randomised controlled trial

Jeremy Segrott (0000-0001-6215-0870), David Gillespie, Mandy Lau, Jo Holliday, Simon Murphy, David Foxcroft, Kerenza Hood, Jonathan Scourfield, Ceri Phillips, Zoe Roberts, Heather Rothwell, Claire Hurlow, Laurence Moore

[1, 2]Jeremy Segrott, Senior Lecturer, segrottj@cardiff.ac.uk
[1]David Gillespie, Senior Research Fellow, gillespied1@cardiff.ac.uk
[1]Mandy Lau, Research Associate, lautm@cardiff.ac.uk
[3]Jo Holliday, Health Data Group Lead, jo.holliday@ndph.ox.ac.uk
[1]Simon Murphy, Professor in Social Interventions and Health, Director DECIPHer and Lead for the Schools Health Research Network, murphys7@cardiff.ac.uk
[4]David Foxcroft, Professor of Community Psychology and Public Health, david.foxcroft@brookes.ac.uk
Kerenza Hood, Director of the Centre for Trials Research, hoodk1@cardiff.ac.uk
[5]Jonathan Scourfield, Professor of Social Work, scourfield@cardiff.ac.uk
[6]Ceri Phillips, Professor Emeritus, c.j.phillips@swansea.ac.uk
[7]Zoe Roberts, Lecturer robertsze@cardiff.ac.uk
[2]Heather Rothwell, Retired
[8]Claire Hurlow, Study Manager, c.e.hurlow@swansea.ac.uk
[9]Laurence Moore, Director, MRC/CSO Social & Public Health Sciences Unit, Laurence.Moore@glasgow.ac.uk

[1]Centre for Trials Research, Cardiff University, Heath Park, Cardiff, UK. CF14 4YS
[2]DECIPHer Centre, Cardiff University, 1-3 Museum Place, Cardiff, UK. CF10 3BD.
[3]UK Biobank, Big Data Institute, University of Oxford, Li Ka Shing Centre for Health Information and Discovery, Old Road Campus, Roosevelt Drive, Oxford, UK. OX3 7BN
[4]Department of Psychology, Health and Professional Development, Faculty of Health and Life Sciences, Oxford Brookes University, Headington Campus, Oxford, UK. OX3 0BP.
[5]School of Social Sciences, Cardiff University, Glamorgan Building, King Edward VII Avenue, Cardiff, UK. CF10 3WT.
[6]College of Human and Health Sciences, Swansea University, Singleton Park, Swansea, UK. SA2 8PP.
[7]Centre for Medical Education, School of Medicine, Cardiff University, Heath Park, Cardiff, UK. CF14 4XN
[8]Swansea Trials Unit, Swansea University Medical School, Swansea University, Singleton Park, Swansea, UK. SA2 8PP.
[9]MRC/CSO Social & Public Health Sciences Unit, University of Glasgow, Berkeley Square, 99 Berkeley Street, Glasgow, UK. G3 7HR.

Correspondence to: Dr J Segrott, Centre for Trials Research, Cardiff University, 4th Floor, Neuadd Meirionnydd, Heath Park, Cardiff, CF14 4YS. Email: segrottj@cardiff.ac.uk

Word count for main text: 5008

For peer review only - http://bmjopen.bmj.com/site/about/guidelines.xhtml

Abstract

Objectives
The Strengthening Families Programme 10–14 (SFP10-14) is a USA-developed universal group-based intervention aiming to prevent substance misuse by strengthening protective factors within the family. This study evaluated a proportionate universal implementation of the adapted UK version (SFP10-14UK) which brought together families identified as likely/not likely to experience/present challenges within a group setting.

Design
Pragmatic cluster-randomised controlled effectiveness trial, with families as the unit of randomisation and embedded process and economic evaluations.

Setting
The study took places in seven counties in Wales, UK.

Participants
715 families (919 parents/carers, 931 young people) were randomised. Primary analysis included 746 young people (80.1%) (alcohol consumption) and 732 young people (78.6%) (drunkenness).

Interventions
Families randomised to the intervention received the Strengthening Families Programme 10–14 (SFP10-14) comprising 7 weekly sessions. Families in intervention and control arms received existing services as normal.

Outcome measures
Primary outcomes were the number of occasions young people reported drinking alcohol in the last 30 days; and drunkenness during the same period, dichotomised as 'never' and '1-2 times or more'. Secondary outcomes examined alcohol/tobacco/substance behaviours including: cannabis use; weekly smoking (validated by salivary cotinine measures); age of alcohol initiation; frequency of drinking >5 drinks in a row; frequency of different types of alcoholic drinks; alcohol-related problems.

Results
There was no evidence of between-group differences 2 years post-randomisation for primary outcomes (young people's alcohol consumption in the last 30 days adjusted odds ratio=1.11, 95% CI: 0.72 to 1.71, p=0.646; drunkenness in the last 30 days adjusted odds ratio=1.46, 95% CI: 0.83 to 2.55, p=0.185). There were no between-group differences for other substance use outcomes, or those relating to wellbeing/stress, and emotional/behavioural problems.

Conclusions
Previous evidence of effectiveness was not replicated. Findings highlight the importance of evaluating interventions when they are adapted for new settings.

Trial registration
Current Controlled Trials ISRCTN63550893.

Keywords
Substance misuse, prevention, RCT, Strengthening Families Programme 10-14(UK), SFP10-14UK, young people, parents, family.

For peer review only

Strengths and Limitations

- Both trial arms had good participant retention rates at main follow-up.
- Recruitment, intervention delivery systems, and implementation replicated how the intervention would be delivered outside a trial setting, thus maximising applicability of findings.
- There were good levels of agreement between self-report of smoking behaviour and cotinine levels in collected samples.
- Most outcomes – including the primary outcomes relating to alcohol, could only be measured using self-report.

For peer review only

Title: The effectiveness of the Strengthening Families Programme in the UK at preventing substance misuse in 10-14 year olds: a pragmatic RCT

Introduction

Prevention of substance misuse among young people is a key public health priority internationally. Early initiation of alcohol consumption and drug use during adolescence is associated with short term negative impacts (e.g. accidental injury, educational attainment) and poorer health over the life course, including substance use disorders and mental health problems. Family-based risk and protective factors, encompassing parental modelling of alcohol use,[1] substance use rules and monitoring,[2] and parenting styles and family relationships, have been identified as important,[3] and influence a range of outcomes alongside substance use such as adolescent mental health.[4] This has led to considerable policy interest in developing prevention interventions targeting key parenting/family variables[5] and a need to develop the international evidence base.[6]

The Strengthening Families Programme 10–14 (SFP10-14) is a USA-developed universal group-based intervention that aims to prevent substance misuse in young people by strengthening protective factors within the family.[7] It comprises 7 weekly sessions each lasting two hours. In the first hour parallel groups of young people and parents/carers from up to 12 families develop their understanding and skills, led by trained facilitators. Each session is guided by a facilitator manual which details activities and how they are to be delivered. In the second hour parents/carers and young people come together in family units to practise key skills. The programme has been culturally adapted for the UK (SFP10-14UK). A logic model for the culturally adapted intervention has been developed by the authors[8].

Trials of the programme in the USA using a universal delivery model have demonstrated its effectiveness in delaying the onset of alcohol use, reducing uptake of smoking, the incidence of harder drug use (methamphetamine)[9 10] (6.5 years past baseline) and substance use at 10 year follow-up.[11] However, findings of the trials conducted in the USA have been criticised in relation to sample 'selection bias', low rates of intervention attendance, and data analysis and reporting practices.[12 13] The current study evaluates the version of the intervention that was culturally adapted for the UK (SFP10-14UK).

Universal interventions (provided without recourse to criteria relating to risk/need) may inadvertently perpetuate inequalities because of their sometimes low reach among those most at need.[14] Targeted interventions may also face difficulties in identifying and recruiting such groups, create barriers to engagement through stigmatisation of potential participants[15] and generate iatrogenic effects through reinforcement and normalisation of harmful norms.[16 17] Targeted interventions – which bring together families with high levels of need, may also present problems in relation to feasibility of delivering activities with fidelity.[18] A distinctive aspect of this trial is that the intervention was implemented using a proportionate universal (PU) approach – combining universal provision but with variation in its intensity/scale varied according to need.[19] This aimed to address some of the limitations of both universal and targeted approaches described above and operationalise delivery of a universal intervention within an existing UK system mainly geared towards targeted interventions.

The proportionate universalism approach adopted had two key components. First, at recruitment stage the intervention was provided on a universal basis but with greater efforts and resources dedicated to recruiting those families with higher level support needs. Second, groups of families brought together for the intervention were composed of approximately 30% who were likely to experience or present challenges within a group setting - e.g. young people not attending or excluded from school/class/year group or working with behavioural support teams; Autistic Spectrum Disorder; aggression/anger; low literacy skills; learning difficulties; mental health issues; English/Welsh not being a family's first language; and substance misuse (parent or young person), and 70% without such challenges. This group composition was designed to: enable delivery of activities as intended, promote prosocial group dynamics, and support families with higher levels of support, with the goal of facilitating behaviour change processes hypothesized to generate the intervention's intended impacts.

Aim

The trial's primary objective was to assess the effectiveness of the SFP 10-14 UK in preventing alcohol misuse in young people. Its secondary objectives were to assess the programme's impact on drug misuse, smoking behaviour, alcohol initiation and drink-related problems and school performance, among young people. Tertiary objectives were to measure the extent to which SFP 10-14 UK had effects on young people's mental health and wellbeing, and protective factors for alcohol and tobacco use/misuse (e.g. family functioning, parenting and young people's peer resistance skills). The trial measured the costs associated with programme delivery and whether the intervention was cost effective. It assessed if there were important variations in delivery and receipt across trial sites, and identified key programme theory, content, and processes.

For peer review only - http://bmjopen.bmj.com/site/about/guidelines.xhtml

Methods

Study design

The trial comprised a pragmatic cluster randomised controlled effectiveness trial with families as the unit of randomisation, and all individuals within a family recruited into the study allocated to the same arm. There were embedded process and economic evaluations. The study took place in seven counties of Wales, UK. Ethical approval for the trial was given by the Research Ethics Committee for Wales (reference 09/MRE09/53). Full details of the design, methods and outcomes have been published in the study protocol.[20] Programme delivery – including staffing arrangements and participant eligibility were designed to mirror future 'real world' implementation outside of a research context to maximise generalisability of findings. The researchers did not impose any additional standardisation of intervention delivery. Local authorities and third sector organisations with responsibility for supporting families or substance misuse prevention led multiple agency partnerships that delivered the programme in community settings. The trial was prospectively registered with ISCTRN registry on 3rd December 2019, and prior to any participants being enrolled.

Participants

SFP 10–14 UK was offered to families with young people aged 10–14. Each programme was open to families from a loosely defined and large geographical area (e.g. town or rural district). Self-referrals to the Programme came forward in response to awareness-raising in community and educational settings. Other referrals came from agencies such as education, health and social services which identified families that may benefit from participating in the Programme. Potential referrers and families were informed that the Programme was being run as part of a trial and were provided with brief information about the trial. When a family was referred or applied to the SFP 10–14UK they were visited by a member of the programme delivery team (normally the programme coordinator) who undertook a needs and eligibility assessment. Based on the information contained in the family referral/application form and the needs and eligibility assessment, the coordinator determined if eligible families were likely to experience or present challenges within a group setting. If families were deemed eligible to attend the programme they were asked if they were willing for a member of the research team to have access to their referral notes and to visit them. Where the family agreed, a research fieldworker visited them to obtain informed consent to participate in the trial and collect baseline data using a computer assisted personal interview (CAPI). At the end of the interview, remote computerised randomisation was used to allocate families to intervention or control arm and the research fieldworker informed the family to which arm they had been randomised.

Any family deemed eligible to attend SFP 10–14 UK was included in the research trial, subject to their giving consent. Trial inclusion and exclusion criteria are listed in Table 1. Informed consent was obtained from all participants. For a family to be included in the research trial at least one parent/carer needed to consent to participate, and parent/carer consent for the inclusion of at least one young person in the trial was required. Young people also gave consent for their participation in the trial.

For peer review only

For peer review only - http://bmjopen.bmj.com/site/about/guidelines.xhtml

Table 1: Trial inclusion and exclusion criteria

Inclusion criteria	Exclusion criteria
Families in which at least one parent/carer and one young person are willing to attend the programme together	Families in which either a parent or young person does not want to attend the programme
Families with the ability to speak English (help can be provided for parents or young people with low literacy levels). Some programmes may also be delivered through the medium of Welsh if there is sufficient demand	Families in which there are either parents or young people who cannot speak English (or Welsh, where appropriate)
Families where a programme is being offered at a location to which it is practicable for a family to travel (as determined by the programme coordinator) within the next three months	Families where there is no programme being offered at a location to which it is practicable for a family to travel (as determined by the programme coordinator) within the next three months. In such a case the family would not be excluded. They will be placed on a waiting list for the programme and will be contacted when a programme is available. They will then be recruited into the trial
Families with a young person aged 10-14	-
-	Families who do not live together - e.g. the child/children are in care
-	Families with very high needs or challenges (such as serious substance misuse problems, family breakdown or crisis)

Randomisation and masking Procedures

Remote computerised randomisation was conducted by the research fieldworker at the end of the baseline interview once consent and baseline data had been obtained. Families were randomised within strata defined by the seven areas in which the study was conducted and using minimisation with a random element set at 80%. The minimisation algorithm aimed to achieve balance between randomised groups on family categorisation (general population/family with challenges), the average age of the young people within a family who were recruited into the trial (<12/12+), and the number of young people within a family who were recruited into the trial (1/>1). Families randomised to the intervention arm received SFP10-14UK in addition to usually provided services. Those randomised to the control continued to receive usually provided services only. These services included local authority parenting and family services, school-based support, and other providers' services such as substance misuse prevention teams, child, and adolescent mental health (CAMHS) and programmes operated by charities. There was some variation in the provision and organisation of services across the trial areas. Data on families' health, social care, education, and criminal justice service utilisation was collected from parents/carers at 9, 15 and 24 month follow-up points. Due to the nature of the intervention, it was not possible for participants or those delivering the intervention to be blinded to families' group assignment. Assessment of trial outcomes was done blind to group assignment.

Outcomes

The trial had two primary outcomes - the number of occasions that young people reported having drunk alcohol in the last 30 days; and drunkenness during the last 30 days, dichotomised as 'never' and '1-2 times or more'. Secondary outcomes were: reported use of cannabis (ever vs. never); weekly smoking (yes vs no, validated by salivary cotinine measures); age of alcohol use initiation; frequency of drinking more than 5 drinks in a row in the last 30 days; frequency of different types of alcoholic drinks; alcohol-related problems; and General Certificate of Secondary Education (GCSE) performance at age 15/16 (number of GCSEs passed and grades achieved, measured as a continuous outcome). All primary and secondary outcomes were collected from children at 2 year follow-up, with the exception of GCSE results, which it was proposed to collect via the Secure Anonymised Information Linkage (SAIL) Databank[21], once all participants had completed GCSEs/left compulsory education.

The trial's tertiary outcomes were: parenting (General Child Management Scale child report); family functioning (Family Relationship Index); children's wellbeing and stress (Strengths and Difficulties Questionnaire scores [SDQ]); children's health status (SF-36); parents/carers' health status (General Health Questionnaire - GHQ and EQ5D); indicators of relative cost-effectiveness (derived from the health data); children's smoking status (i.e. whether they have ever smoked/smoked now); young people's self-efficacy; age

For peer review only - http://bmjopen.bmj.com/site/about/guidelines.xhtml

of first cigarette; and age of first drug use. Tables S1-S2 (Supplementary material) provide a description of the trial's primary, secondary and tertiary outcomes, and the specific measures used at main follow-up at 24 months.

Selection of outcomes and length of follow-up were driven by the intervention's hypothesized mechanisms of action and impacts, including long term substance use prevention, and short/medium term mediators, particularly family relationships and parenting.

Procedures were put in place to assess and respond to reports of adverse events. Serious Adverse Events (SAEs) were reviewed by a senior member of the trial team within 24 hours to assess the nature of the SAE, its seriousness, causality, and expectedness. Where an SAE was both related (resulting from administration of any of the research procedures) and unexpected the Trial Manager notified the REC within 15 days of receiving notification of the SAE.

Statistical analysis

We aimed to randomise 756 families in total (378 per arm). Our target sample size provides 80% power with a two-sided alpha at 0.025 (halved to account for two primary outcomes) to detect either a 12 percentage point difference in young people reporting having drunk alcohol at the 24-month follow-up point (assuming a control group prevalence of 48%)[22] or a 10 percentage point difference in young people reporting having been drunk at the 24-month follow-up point (assuming a control group prevalence of 22%)[22]. The sample size was inflated based on the average family including 1.25 young people in the trial, an intracluster correlation coefficient of 0.2, and 25% loss to follow-up.

Descriptive statistics were calculated using means (standard deviation), medians (interquartile range) and percentages as appropriate.

The primary analyses were conducted on a modified intention to treat population (participants who provided outcome data analysed in the arm to which they were randomised) and involved fitting two-level logistic regression models to whether or not young people reported drinking alcohol/being drunk in the 30 days prior to their 24-month interview, with responses from young people nested within families. The models controlled for the variables balanced on at randomisation (other than number of young people, as this was balanced on to ensure equal number of young people per groups, rather than scientific reasons), the study area, and the baseline-reported alcohol use/drunkenness (depending on the outcome). To test the strength of the findings of the primary analyses, the models were re-analysed controlling for gender, time from randomisation to 24-month follow-up, and without controlling for any covariates (pre-planned analyses). The primary outcomes were also analysed as ordinal variables (i.e. the number of occasions in the last 30 days that young people reported drinking alcohol/being drunk). The three categories used in the analysis were 'no occasions', '1-2 occasions' and '3 or more occasions'. Two-level ordinal regression models were fitted to both outcomes, with and without adjusting for variables balanced on at randomisation and baseline alcohol use/drunkenness.

In order to assess the impact that intervention non-receipt had on the findings of the primary analyses, double logistic structural mean models using generalised method of moments were fitted to each primary outcome.[23] A binary definition of intervention receipt was agreed by the study team a priori which involved participants attending at least five sessions, without missing more than one session in a row. The quantitative measure of intervention receipt was also used (i.e. the number of weeks attended).

Multiple imputation was used to assess the impact of missing primary outcome data on the conclusions drawn. Additional sensitivity analyses were also performed using more extreme assumptions about missing primary outcome data. For further details, see the Supplementary material.

Secondary outcomes were analysed by fitting two-level logistic regression models (proportion of young people at 24 months classing themselves as weekly smokers, reporting having ever used cannabis, having used cannabis in the previous 12 months, having used cannabis in the previous 30 days, and having experienced at least one of the listed alcohol-related problems in the previous 12 months) and two-level Cox proportional hazards models (time to initiation of alcohol, smoking, and drugs – in those participants who had not initiated at baseline). Covariate adjustment for secondary analyses mirrored that used for the primary analyses.

Potential modifiers of the intervention with respect to both primary outcomes were investigated by fitting interaction terms to trial arm and pre-planned subgroups (age, gender, and smoking behaviour of the young person, drinking behaviour of the parents/carers, socioeconomic status assessed using the Family Affluence Scale (low, medium or high) and highest occupational status of parents/carers (categories based on the NS-SEC

For peer review only - http://bmjopen.bmj.com/site/about/guidelines.xhtml

self-coded method), family categorisation (family likely to experience challenges in a group setting / family without challenges in a group setting), young person reported SDQ, domains of the Family Relationship Index, and averaged young person and parent/carer scores of General Child Management scale at baseline).

As it was one of the distinguishing features of this trial, further post-hoc subgroup analyses were conducted to investigate potential differential intervention effects by family categorisation for the smoking, cannabis use, and SDQ outcomes, the latter being included because of its focus on mental health, conduct problems, and peer influences.

All analyses were conducted using STATA version 13.0.

Role of funder

The study funder was not involved in the design of the study, or collection, analysis, or interpretation of the data. The corresponding author had access to all data in the study and had final responsibility for the decision to submit for publication.

Patient and Public Involvement

Members of the public were involved in refining and piloting of outcome measures and study procedures. This included a mix of families who had already received SFP10-14 (and who were able to comment on the perceived relevance of proposed outcomes) and those without any prior knowledge of the intervention (and were therefore able to provide input on how trial participants with no previous knowledge might perceive study materials). Piloting comprised asking participants to complete baseline interviews with researchers, and then provide feedback on clarity of written information, time taken to complete the baseline interview, and the acceptability and appropriateness of outcome measures. This information was then used to refine the final selection of outcome measures and format of information sheets, etc. Input was also sought from a young people's research advisory group (ALPHA), led by a public involvement officer embedded within the study team. The public involvement officer formed stakeholder groups – formed of trial participants, to advise the study team at key points on recruitment and retention strategies, maintaining contact with the trial cohort, and plans for disseminating trial findings.

Results

Participant recruitment took place between 8th February 2010 and 18th June 2012. Seven hundred and fifteen families were recruited into the trial. This comprised 918 parents/carers and 931 young people. The number of participants completing questionnaires at each assessment point is included in the trial profile (Figure 1). Primary outcome data was collected from young people. The primary analyses included 746 young people for the alcohol use outcome at two-years post-randomisation (80.1%) and 732 for the drunkenness outcome at two-years post-randomisation (78.6%). Completion rates were similar across trial arms. Analysis of levels of service utilisation data at 9, 15 and 24 month follow up did not identify any discernible differences between the intervention and control arms.

For peer review only

Baseline characteristics

Table 2 illustrates the characteristics of parents/carers and young people at baseline in control and intervention groups. Randomised groups were broadly similar at baseline, with small differences noted for young person gender and parent/carers' reported cigarette use.

Table 2: Baseline characteristics for Parent/carers and young people according to experimental group

Parent/carer baseline characteristics		Control (n=457)	SFP (Intervention) (n=461)
Age (Median, IQR)		37.0 (32.0 to 43.0)	37.0 (33.0 to 42.5)
Gender	Female	105 (23.0%)	103 (22.3%)
	Male	352 (77.0%)	358 (77.7%)
Ethnic group	White	451 (98.7%)	458 (99.6%)
	Non-white	6 (1.3%)	2 (0.4%)
Relationship status	Single, not in a relationship	54 (12.0%)	68 (14.8%)
	Single, in a relationship	97 (21.5%)	91 (19.8%)
	Married/ civil partnership	200 (44.3%)	183 (39.9%)
	Separated/ divorced	95 (21.1%)	113 (24.6%)
	Widowed	4 (0.9%)	3 (0.7%)
	Other	1 (0.2%)	1 (0.2%)
Ever smoked a cigarette	Never	231 (50.5%)	207 (44.9%)
	Yes, at least once	226 (49.5%)	254 (55.1%)
Ever drunk alcohol	Never	85 (18.6%)	90 (19.5%)
	Yes, at least once	372 (81.4%)	371 (80.5%)
AUDIT-C	Low risk	304 (66.5%)	294 (64.6%)
	High risk	153 (33.5%)	161 (35.4%)
Ever taken drug	Never	300 (65.6%)	307 (66.6%)
	Yes, at least once	157 (34.4%)	154 (33.4%)
Deprivation overall score (Median, IQR)		27.5 (18.5 to 41.2)	29.4 (18.0 to 44.5)
Young people baseline characteristics		Control (n=454)	SFP (n=477)
	Age (Median, IQR)	11.5 (10.0 to 13.0)	12.0 (11.0 to 13.0)
Gender	Female	231 (50.9%)	269 (56.4%)
	Male	223 (49.1%)	208 (43.6%)
Ethnic group	White	446 (98.5%)	474 (99.6%)
	Non-white	7 (1.5%)	2 (0.4%)
Family affluence scale	Low (0-2)	83 (18.4%)	99 (20.9%)
	Medium (3-5)	234 (52.0%)	231 (48.8%)
	High (6-9)	133 (29.6%)	143 (30.2%)
Ever smoked a cigarette	Never	330 (72.7%)	348 (73.0%)
	Yes, at least once	124 (27.3%)	129 (27.0%)
Ever drunk alcohol	Never	314 (69.3%)	326 (68.5%)
	Yes, at least once	139 (30.7%)	150 (31.5%)
Ever taken drug	Never	433 (95.4%)	450 (94.3%)
	Yes, at least once	21 (4.6%)	27 (5.7%)
Child-deprivation overall score (Median, IQR)		28.1 (19.7 to 39.2)	28.2 (18.3 to 39.6)

Primary analysis

Table 3 shows the results of alcohol use and drunkenness in the previous 30 days prior to their 24-month follow-up interview for young people. Young people allocated to the SFP group had higher alcohol use and drunkenness prevalence than those allocated to the control group (26.4% versus 24.6% for alcohol use, 10.2% versus 8.3% for drunkenness). However, results from the multilevel models indicated insufficient evidence to reject the null hypothesis of no difference between groups (alcohol use adjusted odds ratio 1.11 [95% CI 0.72 to 1.71], p = 0.646; drunkenness AOR = 1.46 [95% CI 0.83 to 2.55], p = 0.185). Conclusions were unaffected by pre-planned secondary analyses of the primary outcomes (see Supplementary material – tables S3-4).

For peer review only - http://bmjopen.bmj.com/site/about/guidelines.xhtml

Table 3: Primary alcohol use and drunkenness outcomes

Outcome	ICC*	Proportion of events in control arm (%)	Proportion of events in SFP arm (%)	Adjusted odds ratio†	95% Confidence Interval		p-value
					Lower limit	Upper limit	
Alcohol use in previous 30 days‡	0.25	85/345 (24.6)	106/401 (26.4)	1.11	0.72	1.71	0.646
Drunkenness in previous 30 days§	0.18	28/338 (8.3)	40/394 (10.2)	1.46	0.83	2.55	0.185

**Intracluster correlation coefficient: proportion of the total variance (in whether or not a young person reports having used alcohol/been drunk in the 30 days prior to their 24-month follow-up interview) attributable to differences between families. Calculated using the standard $\pi^2/3$ estimator for binary outcomes. †Analyses adjusted for variables that were balanced on at randomisation (study area, family categorisation and average age of young people within the family) and baseline alcohol use / drunkenness (corresponding with the outcome). ‡Number of occasions in the last 30 days that participant has drunk alcohol (asked at 24-month follow-up) – dichotomised to 0/1 or more occasions. § Number of occasions in the last 30 days that participant has been drunk (asked at 24-month follow-up) – dichotomised to 0/1 or more occasions.*

Subgroup analysis for primary outcomes

Tables S5 and S6 (Supplementary material) provide the results of the planned subgroup analysis for alcohol use and drunkenness in the previous 30 days. There was no evidence of any differential intervention effect for any of the subgroups for either primary outcome.

Other substance use secondary outcomes

There was no evidence of difference between groups for the other substance use outcome (Table 4). Rates of agreement between self-reported weekly smoking behaviour and saliva cotinine are show in Table S7 (supplementary material).

For peer review only

Table 4: Other substance use secondary outcomes

Outcome		ICC*	Proportion of events in control arm (%)	Proportion of events in SFP arm (%)	Adjusted odds ratio	95% Confidence Interval		p-value
						Lower limit	Upper limit	
Alcohol-related problems in the last 12 months		0.18	51/326 (15.6)	60/384 (15.6)	0.95	0.59	1.54	0.849
Currently smoke at least one cigarette a week		0.41	52/347 (15.0)	59/402 (14.7)	0.94	0.53	1.66	0.828
Ever taken cannabis			46/338 (13.6)	58/393 (14.8)	1.21	0.57	2.55	0.617
Taken cannabis at least once in the last 12 months		0.43	41/337 (12.2)	49/393 (12.5)	1.01	0.51	1.97	0.985
Taken cannabis at least once in the last 30 days			20/338 (5.9)	21/393 (5.3)	0.77	0.36	1.68	0.518
Time to alcohol initiation†			88/246 (35.8)	81/273 (29.7)	0.86	0.64	1.17	0.344
Frequency of consuming 5+ drinks in a row in the last 30 days‡	0	0.00	315/345(91.3)	348/401 (86.8)	1.55	0.95	2.51	0.080
	1 to 2		15/345(4.3)	36/401 (9.0)				
	3 to 5		15/345(4.8)	17/401 (4.2)				
Frequency of drinking beer‡	Never	0.00	290/346 (83.8)	331/401 (82.5)	1.15	0.77	1.72	0.492
	Rarely		20/346 (5.8)	17/401 (4.2)				
	Every month		26/346 (7.5)	36/401 (9.0)				
	At least once a week		10/346 (2.9)	17/401 (4.2)				
Frequency of drinking wine‡	Never	0.00	323/345 (93.6)	375/402 (93.3)	1.06	0.59	1.92	0.837
	Rarely		5/345 (1.4)	7/402 (1.7)				
	At least once a month		17/345 (4.9)	20/402 (5.0)				
Frequency of drinking spirits‡	Never	0.71	298/346 (86.1)	351/401 (87.5)	0.85	0.50	1.46	0.567
	Rarely		15/346 (4.3)	13/401 (3.2)				
	Every month		21/346 (6.1)	24/401 (6.0)				
	At least once a week		12/346 (3.5)	13/401 (3.2)				
Frequency of drinking alcopops‡	Never	0.35	303/346 (87.6)	345/402 (85.8)	1.24	0.78	1.97	0.361
	Rarely		19/346 (5.5)	17/402 (4.2)				
	At least once a month		24/346 (6.9)	40/402 (10.0)				
Frequency of drinking cider‡	Never	0.00	305/346 (88.2)	346/402 (86.1)	1.26	0.80	1.97	0.313
	Rarely		12/346 (3.5)	13/402 (3.2)				
	At least once a month		29/346 (8.4)	43/402 (10.7)				

**Calculated using the standard $\pi^2/3$ estimator for binary outcomes. †Single level Cox regression; Percentage of young people who initiated alcohol after baseline; adjusted hazard ratio. ‡Multilevel ordinal regression. §multilevel linear regression; mean (SD); adjusted mean difference*

For peer review only

GCSE Performance at age 15/16
Based on the analysis of the primary and secondary self-reported outcomes at 2 year follow-up, it was determined that collection of the GCSE data was not justified.

Post-hoc subgroup analysis
Table 5 provides the results of unplanned subgroup analysis aiming to explore differential intervention effects on the secondary substance use outcomes and SDQ score by whether or not the family was likely to experience/present challenges in a group setting. The motivation for this exploratory work stemmed from the subgroup findings for the two primary outcomes, which demonstrated small (not statistically significant) differential effects favouring families experiencing/presenting challenges within a group setting. While all but the SDQ subgroup analysis are not statistically significant, the pattern found for the primary outcomes (i.e. differential outcomes favouring families with challenges) was reflected across the additional outcomes investigated. Taking the 'cannabis' outcome as one example, we see that the odds of reporting cannabis use in the last 12 months are higher for the main effect (i.e. higher for those in a family with challenges and allocated to the control arm, compared to families without challenges in a group setting, allocated to the control arm), and the interaction term indicates a reversal of this effect for those in the SFP arm (i.e. cannabis use is higher for families without challenges allocated to the SFP arm than families with challenges allocated to the SFP arm).

Table 5: Post-hoc subgroup analyses for the secondary substance use outcomes and SDQ

Subgroup	Main effect adjusted odds ratio*	95% Confidence Interval		p-value	Subgroup x SFP interaction adjusted odds ratio	95% Confidence Interval		p-value
		Lower limit	Upper limit			Lower limit	Upper limit	
Currently smoke at least one cigarette a week	1.95	0.83	4.55	0.125	0.35	0.11	1.10	0.073
Ever taken cannabis	2.14	0.70	6.58	0.184	0.33	0.07	1.50	0.150
Taken cannabis at least once in the last 12 months	2.11	0.79	5.68	0.138	0.25	0.06	0.99	0.048
Taken cannabis at least once in the last 30 days	1.22	0.39	3.77	0.734	0.30	0.06	1.54	0.150
Subgroup	Adjusted mean difference	95% Confidence Interval		p-value	Subgroup x SFP interaction adjusted mean difference	95% Confidence Interval		p-value
		Lower limit	Upper limit			Lower limit	Upper limit	
Strengths and difficulties total difficulties score	1.77	0.61	2.93	0.003	-2.27	-3.83	-0.71	0.004

*The main effect can be interpreted as the effect of belonging to a family with challenges in a group setting (relative to a family without challenges in a group setting) for those allocated to the control arm.

Headline cost-consequences analysis results
The costs associated with the implementation of the intervention were £1,163,278, which equated to £20,773 per programme and £1,240 per participant, with wide variation in costs across programme areas. There was no difference in costs of healthcare utilisation between the groups at 9 months, 15 months and 24 months. At 9 and 15-months the main cost driver was that of primary care usage, while at 24 months the main driver was in-patient episodes. There was no difference between the groups for adult EQ-5D scores at baseline or 24 months.

EQ-5D scores for adults at both baseline and 24 months were very similar between the intervention and control arms. Conversely, the EQ-5D scores for the children in the intervention arm saw a difference in the scores between baseline and 24 months (a mean increase of 0.043 per participant in the intervention arms, compared to 0.030 in the control).

A complete case analysis was conducted looking at the number of parents who answered the 9, 15 and 24 month questionnaires and who also answered both the baseline and 24 month EQ-5D questionnaire (Intervention

For peer review only - http://bmjopen.bmj.com/site/about/guidelines.xhtml

n=197; Control n=129). The mean EQ5D difference in the control group was 0.053 at a cost difference of £6,271, compared to 0.007 and £7,175 in the intervention group. This resulted in a difference of -0.046 at a cost of £904 with an incremental cost effectiveness ratio (ICER) of -£19,652. Therefore the intervention arm was dominated.

There was no difference in resource utilisation or associated costs between the trial arms at 9, 15 and 24 months. This was the case for resources utilisation in general and specifically to family/parenting-based support services.

Process evaluation

Detailed findings from the process evaluation have been reported.[8] Of the 56 programmes run, 47 (84%) achieved the target group size (5-12 families) at enrolment. Facilitators rated participant engagement high in 94% of activities. There was no evidence that engagement differed by group size. Twenty-two (39%) of the 56 programmes achieved the target composition of families with challenges (30%) and those without challenges in a group setting (70%). However, most of the remaining programmes (39/56) achieved a mix of families which was in line with the intended group composition model (i.e. the majority of families were those without challenges in a group setting).

Overall implementation fidelity was good. Facilitators were asked to rate fidelity of the sessions they delivered using a checklist designed by the SFP10-14 developers. Data were available for 50 of 56 programmes, across which facilitators rated 96% of individual activities as mostly/fully covered – this varied across trial sites from 90-99%. To assess reliability of facilitator reports a sample of 47 sessions from the 50 programmes for which data were available were observed and scored by researcher observers using the same fidelity assessment sheet. Observers rated 77% of observed activities as mostly or fully covered (range 63-88%): ICC scores from 2 observers = 0.73 (95% CI=0.65-0.79). Facilitators' and observers' scores agreed 83% of the time (area range 73-93%).

Recruitment of families was a key implementation challenge. Practitioners reported that the Proportionate Universal approach heightened such challenges as the universal recruitment diverged from the focus of existing systems and referring agencies on families with higher level needs. However, facilitators identified the proportionate universal approach to group composition (with a mix of families with and without challenges within a group setting) as helpful in enabling activities to be delivered as intended, the cultivation of positive group dynamics, and the provision of support to participants with additional needs.

Adverse outcomes

There were no SAEs which were both related (resulting from administration of any of the research procedures) and unexpected.

Discussion

In this paper we have reported findings from a randomised controlled trial of the SFP10-14 following its adaptation for a UK context. We found no evidence of between-group differences 2 years past baseline for the primary outcomes (young people's alcohol consumption and drunkenness in the last 30 days) or for any of the other substance use outcomes. Similarly, there were no between-group differences in relation to young people's wellbeing, stress, and emotional and behavioural problems. Findings were unaltered for pre-planned subgroup analyses. Our study findings contrast with evaluations of SFP10-14 conducted in the USA in which long term effects on substance use outcomes have been detected[9 11]. However, the absence of intervention effects in the UK setting replicates those of more recent European trials in which adapted versions of SFP10-14 have been evaluated. In Sweden no effects on substance use or 'breaking behaviours' were detected[24] though significant adaptations made to the intervention may have undermined its hypothesized mechanisms of action[25]. Likewise, findings from the trial conducted in Poland showed no effects on the primary outcomes (substance use) or aspects of family functioning.[26] An RCT in Germany identified only limited evidence of effectiveness.[27] For young people, there were no differences in rates of substance use, except for tobacco initiation (but only among those followed up at 18 months).

Process evaluation findings indicated that SFP10-14UK was delivered with good fidelity (albeit facilitators' reports were biased towards overstating fidelity), suggesting that our trial was a fair assessment of SFP10-14 when delivered as intended. The Proportionate Universal approach adopted successfully recruited families with differing levels of need (but without the potential stigmatisation generated by targeted interventions), and most groups achieved positive group dynamics in line with hypothesized behaviour change mechanisms.

For peer review only

Our study had several strengths. There were good participant retention rates and high rates of data completion across both trial arms at main follow-up. At 24-month follow-up there were good levels of agreement between self-report of smoking behaviour and cotinine levels in collected samples. Our study was designed as a pragmatic trial in which recruitment, intervention delivery systems, and implementation were designed to replicate how the intervention was likely to be delivered outside a trial setting, thus maximising the applicability of our findings. We did not identify evidence of compensatory provision of services in the control arm, increasing our confidence that the study findings would have detected any intervention effects which were present.

In terms of limitations, by virtue of necessity, all outcomes (including the primary outcomes) were measured using self-report. However, self-report of smoking behaviours was validated using measures of salivary cotinine. The trial utilised multiple statistical tests given the large number of outcomes. All outcomes measured hypothesized impacts of SFP10-14UK and mapped on to the intervention's logic model. In line with similar previous studies of complex interventions[28], we pre-specified primary and secondary outcomes, and also pre-defined subgroup analysis, clearly indicating those subgroup-analyses which were post-hoc.

SFP10-14UK did not replicate the impacts on substance misuse prevention found in the American evaluations. There are a number of possible explanations for the null findings. US trials tended to have longer-term follow-ups and impacts were sometimes stronger in the longer-term (e.g. over 5-10 years). However, the epidemiological trajectory of young people's drinking etc in the UK is earlier than in the US studies so we anticipated any effects to be apparent earlier. The intervention was delivered with good fidelity, and thus implementation failure is unlikely to explain the absence of impacts on hypothesized outcomes. It is possible that 'usual care' (to which SFP10-14UK was added for the intervention group) may vary in important ways between the American context and the European settings into which the intervention has been introduced. Better routine provision of health and welfare services in the UK may have made it more difficult for SFP10-14UK to demonstrate impacts compared with its implementation in the USA. Another possibility is that intervention content may have interacted in different ways with the context of families receiving it. Post-hoc subgroup analysis of substance use-related secondary outcomes appeared to show that for families with challenges in a group setting, differences between intervention and control group were in favour of the intervention group. For families without challenges in a group setting, this pattern was reversed. It is possible that the intervention activities better met the needs of those families identified as likely to experience or present challenges in a group setting, or that key learning had greater fit with their circumstances, and was thus easier to adopt and integrate. However, no such differences according to families with/without challenges in a group setting were identified for our primary outcomes. Whilst some previous trials of SFP10-14 have identified potential patterning of intervention effects by sub groups according to baseline assessment of risk for later substance use[27 29 30], our findings here are not directly comparable, since we were concerned with experience/presentation of challenges within a group setting, and not general levels of support needs or risk of later substance use. Previous research on interventions which are targeted based on assessment of young people's risk behaviours has identified the potential for stigmatisation of participants and generation of iatrogenic effects through reinforcement and normalisation of harmful norms.[16 17] The Proportionate Universal approach adopted by implementation of SFP10-14UK in this study appeared to succeed in reaching families with higher levels of need whilst avoiding the stigmatisation of participants encountered by targeted interventions. However, it is unclear whether the potential intervention effects for families with challenges and the potential harms for families without challenges may be driven by intervention 'fit', or at least derives partly from group dynamics and the ways in which participants interact.

Our trial highlights the importance of assessing the effectiveness of interventions when they are adapted for and then implemented in new settings. Such assessment involves not only a-priori identifying whether an adapted intervention remains faithful to its original logic model, but also understanding how it interacts with the new context into which is introduced. This context includes both the existing provision of services, and the needs of the participants who receive it.

Author Contributions

JSe wrote the first draft of the paper with input from other authors and led the process evaluation.
DG led the design and conduct of statistical analysis and assisted with writing the first draft of the paper and revisions.
ML contributed to statistical analysis and the writing of the first draft of the paper and subsequent drafts
JH was a trial manager and collected and analysed data on recruitment and retention.
SM provided input on the design, conduct and analysis of data for the process evaluation.
DF advised on the design of the study, the selection of outcome measures and the interpretation of the results.

For peer review only - http://bmjopen.bmj.com/site/about/guidelines.xhtml

KH contributed to the overall design of the study the design of the trial's statistical analysis plan, and the analysis of trial data.
JSc provided input on data collection and analysis within the process evaluation.
CP designed and led the health economic evaluation.
ZR contributed to the design and conduct of statistical analysis.
HR conducted data collection and analysis for the process evaluation.
CH was a trial manager and contributed to data collection, collation of data on recruitment and retention.
LM was chief investigator and led the overall design and conduct of the study.

All authors contributors contributed to the writing of the paper.
Jeremy Segrott is responsible for the overall content as guarantor, accepts full responsibility for the work and the conduct of the study, had access to the data, and controlled the decision to publish. The corresponding author attests that all listed authors meet authorship criteria and that no others meeting the criteria have been omitted.

Competing interests
All authors have completed the ICMJE uniform disclosure form at www.icmje.org/coi_disclosure.pdf and declare financial support from the National Prevention Research Initiative, Welsh Government and Cardiff Strengthening Families Team. DF's institution has previously received financial support for the development of the SFP 10–14 UK programme materials from the alcohol industry; no other relationships or activities that could appear to have influenced the submitted work.

Funding
Funding of £2.1 M from the National Prevention Research Initiative, managed by the Medical Research Council – award G0802128, included c.£650 k implementation costs. The NPRI funding partners are Alzheimer's Research Trust; Alzheimer's Society; Biotechnology and Biological Sciences Research Council; British Heart Foundation; Cancer Research UK; Chief Scientist Office, Scottish Government Health Directorate; Department of Health; Diabetes UK; Economic and Social Research Council; Engineering and Physical Sciences Research Council; Health & Social Care Research & Development Office for Northern Ireland; Medical Research Council; The Stroke Association; Welsh Government; and World Cancer Research Fund.

A representative from the study funders was a member of the trial's Independent Trial Steering Committee. The Welsh Government provided c.£675 k of partnership funding, to cover the cost of implementation in three trial areas, and the associated training and support provided by the Cardiff Strengthening Families Programme team. Further support from Welsh Government provided £208k to cover programme delivery in six trial sites from August 2011-July 2012. The Cardiff Strengthening Families Programme team also provided financial support for programme delivery and trial recruitment in schools.

At the time of the study DECIPHer was a UKCRC Public Health Research Centre of Excellence. Funding from the British Heart Foundation, Cancer Research UK, Economic and Social Research Council (RES-590-28-0005), Medical Research Council, the Welsh Government and the Wellcome Trust (WT087640MA), under the auspices of the UK Clinical Research Collaboration, is gratefully acknowledged. DECIPHer funding has supported JSe and JH's input into the trial. The centre is now funded by Welsh Government through Health and Care Research Wales. DECIPHer funding has supported JSe and JH's input into the trial.

LM is supported by the Medical Research Council (MC_UU_12017/14) and Chief Scientist Office (SPHSU14).

The Centre for Trials Research is funded by Health & Care Research Wales and Cancer Research UK.

Acknowledgements
We would like to thank the parents/carers and young people who participated in the trial. Grateful thanks are also due to the delivery teams who implemented SFP10-14UK as part of the trial, the independent trial steering committee and data monitoring committee, and the Participant Resource Centre at Cardiff University.

Data sharing
Researchers can request access to de identified participant data by contacting the corresponding author. Requests for access to data will be subject to approval of a proposal, completion of a signed data access agreement, and compliance with institutional and legal requirements. The study protocol is freely available, and the statistical analysis plan is available upon request.

For peer review only - http://bmjopen.bmj.com/site/about/guidelines.xhtml

Transparency Declaration
The lead author (the manuscript's guarantor) affirms that the manuscript is an honest, accurate, and transparent account of the study being reported; that no important aspects of the study have been omitted; and that any discrepancies from the study as originally planned (and, if relevant, registered) have been explained.

Licence Statement
I Jeremy Segrott, the Submitting Author has the right to grant and does grant on behalf of all authors of the Work (as defined in the below author licence), an exclusive licence and/or a non-exclusive licence for contributions from authors who are: i) UK Crown employees; ii) where BMJ has agreed a CC-BY licence shall apply, and/or iii) in accordance with the terms applicable for US Federal Government officers or employees acting as part of their official duties; on a worldwide, perpetual, irrevocable, royalty-free basis to BMJ Publishing Group Ltd ("BMJ") its licensees and where the relevant Journal is co-owned by BMJ to the co-owners of the Journal, to publish the Work in BMJ Open and any other BMJ products and to exploit all rights, as set out in our licence.

The Submitting Author accepts and understands that any supply made under these terms is made by BMJ to the Submitting Author unless you are acting as an employee on behalf of your employer or a postgraduate student of an affiliated institution which is paying any applicable article publishing charge ("APC") for Open Access articles. Where the Submitting Author wishes to make the Work available on an Open Access basis (and intends to pay the relevant APC), the terms of reuse of such Open Access shall be governed by a Creative Commons licence – details of these licences and which Creative Commons licence will apply to this Work are set out in our licence referred to above.

For peer review only

References

1. Foxcroft DR, Lowe G. Adolescents' Alcohol use and Misuse: The socializing influence of perceived family life. *Drugs: Education, Prevention and Policy* 1997;4(3):215-29. doi: 10.3109/09687639709028544
2. Garmienė A, Žemaitienė N, Zaborskis A. Family time, parental behaviour model and the initiation of smoking and alcohol use by ten-year-old children: an epidemiological study in Kaunas, Lithuania. *BMC Public Health* 2006;6(1):287. doi: 10.1186/1471-2458-6-287
3. Velleman RDB, Templetonl LJ. Substance misuse by children and young people: the role of the family and implications for intervention and prevention. *Paediatrics and Child Health* 2007;17(1):25-30. doi: 10.1016/j.paed.2006.12.002
4. Oldfield J, Humphrey N, Hebron J. The role of parental and peer attachment relationships and school connectedness in predicting adolescent mental health outcomes. *Child and Adolescent Mental Health* 2016;21(1):21-29. doi: 10.1111/camh.12108
5. Cuijpers P. Three Decades of Drug Prevention Research. 2003;10(1):7-20. doi: 10.1080/0968763021000018900
6. Turner KMT, Sanders MR. Dissemination of evidence-based parenting and family support strategies: Learning from the Triple P—Positive Parenting Program system approach. *Aggression and Violent Behavior* 2006;11(2):176-93. doi: 10.1016/j.avb.2005.07.005
7. Molgaard VM, Spoth R, Redmond C. Competency training: The Strengthening Families Program for Parents and Youth 10 142000.
8. Segrott J, Murphy S, Rothwell H, et al. An application of Extended Normalisation Process Theory in a randomised controlled trial of a complex social intervention: process evaluation of the Strengthening Families Programme (10-14) in Wales, UK. 2017 doi: 10.1016/j.ssmph.2017.01.002
9. Spoth R, Redmond C, Shin C, et al. Brief Family Intervention Effects on Adolescent Substance Initiation: School-Level Growth Curve Analyses 6 Years Following Baseline. *Journal of Consulting and Clinical Psychology* 2004;72(3):535-42. doi: 10.1037/0022-006X.72.3.535
10. Spoth RL, Clair S, Shin C, et al. Long-term Effects of Universal Preventive Interventions on Methamphetamine Use Among Adolescents. *Archives of Pediatrics & Adolescent Medicine* 2006;160(9) doi: 10.1001/archpedi.160.9.876
11. Spoth RL, Trudeau LS, Guyll M, et al. Benefits of Universal Intervention Effects on a Youth Protective Shield 10 Years After Baseline. *Journal of Adolescent Health* 2012;50(4):414-17. doi: 10.1016/j.jadohealth.2011.06.010
12. Gorman DM. Letter to the Editor: Sources of bias and need for caution in interpreting the results of Spoth et al.'s (2017) PROSPER study. *Psychological Medicine* 2018;48(4):694-96. doi: 10.1017/s0033291717002355
13. Gorman DM. The decline effect in evaluations of the impact of the Strengthening Families Program for Youth 10-14 (SFP 10-14) on adolescent substance use. *Children and Youth Services Review* 2017;81:29-39. doi: 10.1016/j.childyouth.2017.07.009
14. Dryden R, Williams B, McCowan C, et al. What do we know about who does and does not attend general health checks? Findings from a narrative scoping review. *BMC Public Health* 2012;12(1):723. doi: 10.1186/1471-2458-12-723
15. Bayer JK, Hiscock H, Morton-Allen E, et al. Prevention of mental health problems: rationale for a universal approach. *Archives of Disease in Childhood* 2007;92(1):34-38.
16. Sorhaindo A, Bonell C, Fletcher A, et al. Being targeted: Young women's experience of being identified for a teenage pregnancy prevention programme. *Journal of Adolescence* 2016;49:181-90. doi: 10.1016/j.adolescence.2016.03.013

For peer review only - http://bmjopen.bmj.com/site/about/guidelines.xhtml

17. Dishion TJ, Tipsord JM. Peer Contagion in Child and Adolescent Social and Emotional Development. *Annual Review of Psychology* 2011;62(1):189-214. doi: 10.1146/annurev.psych.093008.100412
18. Segrott J. Recruitment and group composition strategies for family-based substance misuse prevention interventions: an exploratory evaluation. 2013;8(2):89-109. doi: 10.1108/jcs-03-2013-0007
19. Marmott Review. Marmot Review: Fair society, healthy lives: strategic review of health inequalities in England post 2010. London: Institute of Health Equity, 2010.
20. Segrott J, Gillespie D, Holliday J, et al. Preventing substance misuse: study protocol for a randomised controlled trial of the Strengthening Families Programme 10–14 UK (SFP 10–14 UK). *BMC Public Health* 2014;14(1):49. doi: 10.1186/1471-2458-14-49
21. Lyons RA, Jones KH, John G, et al. The SAIL databank: linking multiple health and social care datasets. *Bmc Medical Informatics and Decision Making* 2009;9 doi: 10.1186/1472-6947-9-3
22. Currie C, Zanotti C, Morgan A, et al. Social determinants of Health and Well-Being among Young People. Health Behaviour in School-Aged Children (HBSC) Study: International Report from the 2009/2010 Survey. Health Policy for Children and Adolescents. Copenhagen: WHO Regional Office for Europe, 2012.
23. Vansteelandt S, Goetghebeur E. Causal inference with generalized structural mean models. *Journal of the Royal Statistical Society: Series B (Statistical Methodology)* 2003;65(4):817-35. doi: 10.1046/j.1369-7412.2003.00417.x
24. Skarstrand E, Sundell K, Andreasson S. Evaluation of a Swedish version of the Strengthening Families Programme. *The European Journal of Public Health* 2014;24(4):578-84. doi: 10.1093/eurpub/ckt146
25. Segrott J, Holliday J, Rothwell H, et al. Cultural adaptation and intervention integrity: a response to Skarstrand, Sundell and Andreasson. *The European Journal of Public Health* 2014;24(3):354-55. doi: 10.1093/eurpub/cku039
26. Foxcroft DR, Callen H, Davies EL, et al. Effectiveness of the strengthening families programme 10–14 in Poland: cluster randomized controlled trial. *The European Journal of Public Health* 2016:ckw195. doi: 10.1093/eurpub/ckw195
27. Baldus C, Thomsen M, Sack PM, et al. Evaluation of a German version of the Strengthening Families Programme 10-14: a randomised controlled trial. *European journal of public health* 2016;26(6):953-59. doi: 10.1093/eurpub/ckw082 [published Online First: 2016/07/05]
28. Bonell C, Allen E, Warren E, et al. Effects of the Learning Together intervention on bullying and aggression in English secondary schools (INCLUSIVE): a cluster randomised controlled trial. *The Lancet* 2018;392(10163):2452-64. doi: 10.1016/s0140-6736(18)31782-3
29. Spoth R, Trudeau L, Redmond C, et al. Replicating and extending a model of effects of universal preventive intervention during early adolescence on young adult substance misuse. *Journal of Consulting and Clinical Psychology* 2016;84(10):913-21. doi: 10.1037/ccp0000131
30. Spoth R, Trudeau L, Redmond C, et al. Replication RCT of Early Universal Prevention Effects on Young Adult Substance Misuse. *Journal of consulting and clinical psychology* 2014;82 doi: 10.1037/a0036840

For peer review only - http://bmjopen.bmj.com/site/about/guidelines.xhtml

Figure 1: Trial consort participant flow chart

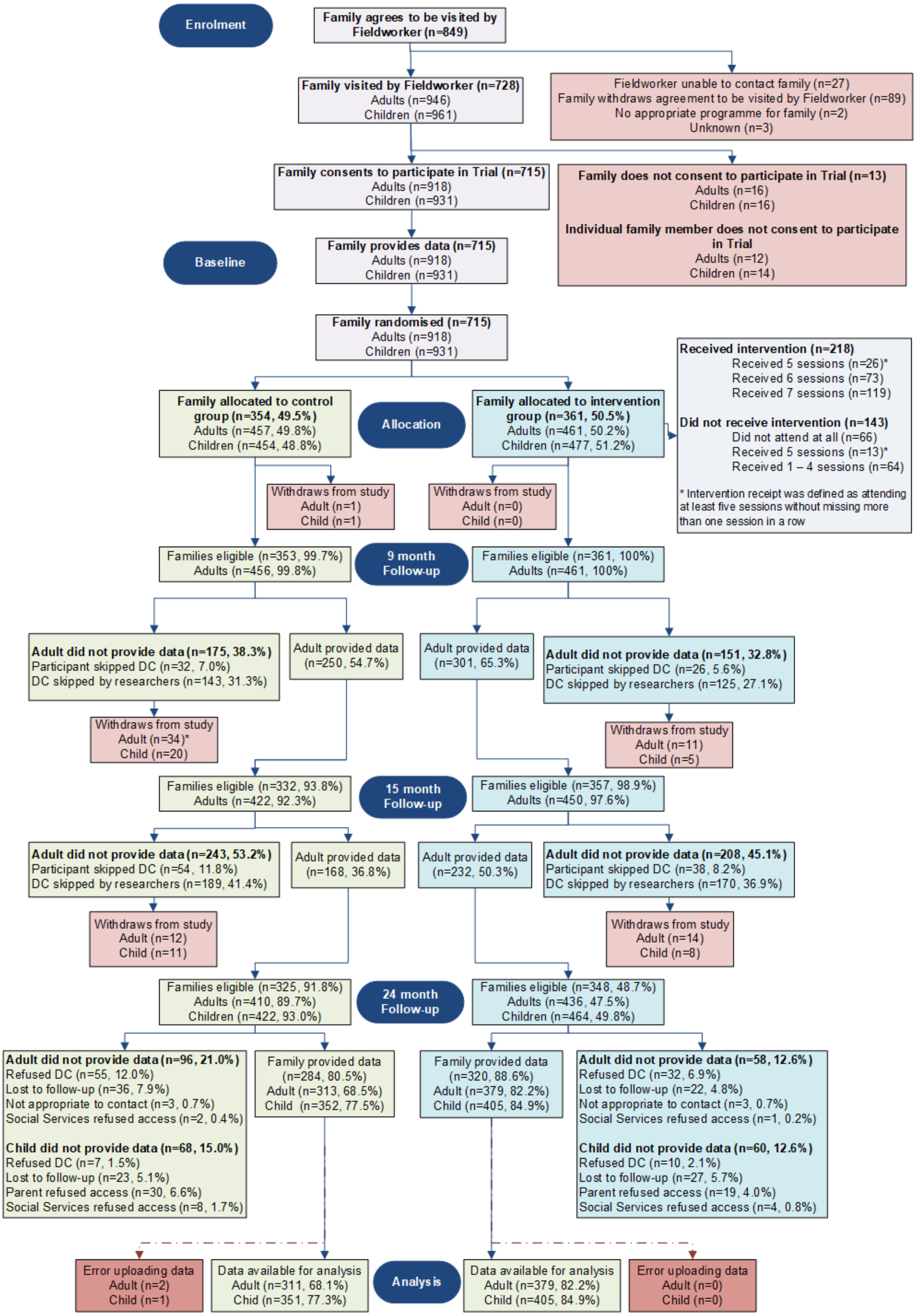

* 32 adults in the Control group withdrew at the 9 month data sweep. A further two adults withdrew prior to being scheduled for their 15 month data sweep

Supplementary material

Table S1: Measures collected at 24 month follow-up from young people

**Domain/question topic**	**Purpose**	**Measure/Source**
**Behaviour - Strengths and Difficulties**		
Strengths and difficulties / wellbeing and stress	TO	SDQ[1]
**Life at Home**		
Family activities (opportunities for involvement in pro-social activities; possibly bonding)	ST	From HBSC/ PEACH study
Young people's own time		From West of Scotland Twenty 07
Parenting/child management	IO/ TO	General Child Management Measure, Project Family[2-4]
Parents and school		Three questions; one adapted from the Child Rearing Practices Measure
Help around the home	IO	Adapted versions of questions asked to parents at 9/15 months.
Development of self-efficacy	ST/ TO	Bandura's Self Efficacy Scale[5]
Attachment to parents	ST	The Security Scale[6]
Befriending pro-social/anti-social peers	ST	Social Development Model scale on friends' anti-social qualities (Interaction with antisocial peers scale)[7]
Positive bonding to school	ST	School Bonding Measure / SDM[8]
**Participant substance use**		
Smoking behaviour – ever smoked?		Question from Substance Initiation Index[9]
Smoking status	TO/ SO	ASSIST study version of NatCen/NFER question[10]
Age first smoked	TO	From HBSC questionnaire
Ever drunk a drink?		NatCen/NFER[11]
Age of first drink	SO	Adapted from HBSC/NatCen/NFER
Drinking frequency, last month (including different types)	SO	Adapted questions from HBSC questionnaire
Ever really drunk?		From HBSC questionnaire
Age first drunk		Adapted version of question in HBSC questionnaire
Number of times drunk, drinking alcohol or 5+ drinks in a row in last month	PO/SO	Amended from HBSC questionnaire
Alcohol-related problems	SO	Q21 from ESPAD survey[12]
Drug use – ever used?		Amended from HBSC/NatCen/NFER[11]
Cannabis use (ever, 12 months, or 30 days)	SO	From HBSC questionnaire
Age of first drug use	TO	Adapted from HBSC questionnaire
**General health**		
Health state today	HE/TO	EQ-5D (child version)[13]
General health	HE/TO	UK Kidscreen 10[14]

For peer review only

Table S2: Measures collected at 24 month follow-up from parents/carers

Domain/question topic	Purpose	Measure/Source
**Life at home**		
Family activities		From HBSC/11-16 West of Scotland adult questionnaire
Family functioning	TO	Family relationship index[15,16]
Parenting/child management	SO, IO	General child management measure, Project Family[2-4]
Parent–child bonding	ST	Adapted from Spoth's rural urban cumulative risk index and Arthur, et al.[17,18]
Parents and school		Question developed by project team and one from Conger's child rearing practices measure
**Participant substance use**		
Smoking behaviour		Heaviness of smoking index[19]
Alcohol use		Adapted AUDIT-C questions, as used by Pre-empt study[20]
Drug use		Adapted from HBSC/NatCen/ NFER[11]
**Health**		
Health status	HE, TO	GHQ[21,22]
Health	HE	1 item from SF-36[23]
Health state today	HE, TO	EQ-5D[13]
**Service utilisation**		
Health, social care, education and criminal justice service utilisation	HE	Modified from ALSPAC

Secondary analyses of the primary outcome

After adjusting alcohol use outcome for gender of young people, time from randomisation to 24 month, and intervention receipt, the results from multilevel modelling indicated that none of the adjustment had altered the results that drawn from the primary analysis (Table 8). Although the alcohol use outcome measure had been restructured into an ordinary outcome measure, the results from ordinal regression model provided a corresponded proportional odds ratio of 1.09 (0.70-1.71) with adjustments and proportional odds ratio of 1.11 (0.73-1.67) without adjustments, which suggests that there was no evidence of a between-group difference.

For peer review only

Table S3: Secondary analyses of the primary alcohol use outcome

Analysis	Odds ratio	95% Confidence Interval		p-value
		Lower limit	Upper limit	
Without any adjustments	1.11	0.74	1.66	0.626
Adjusting for gender of young person	1.11	0.72	1.72	0.641
Adjusting for time from randomisation to 24-month follow-up	1.08	0.70	1.68	0.725
Adjusting for intervention receipt (binary definition)	1.21	0.79	1.86	0.384
Adjusting for intervention receipt (number of weeks)	1.03	0.97	1.09	0.382
Analysis	Proportional odds ratio	95% Confidence Interval		p-value
		Lower limit	Upper limit	
As an ordinal outcome (adjusted)	1.09	0.70	1.71	0.689
As an ordinal outcome (without any adjustments)	1.11	0.73	1.67	0.637

In terms of drunkenness in the previous 30 days, results show that the model without controlling any adjustments reduced the treatment effect odds ratio from 1.46 to 1.27 but did not alter the conclusions that could be drawn from the analysis (Table 9). Similarly, model adjusted for gender for young person, time from randomisation to 24 month, and intervention receipt did not alter the conclusions, which suggests that there was no evidence of a between-group difference.

Table S4: Secondary analyses of the primary drunkenness outcome

Analysis	Odds ratio	95% Confidence Interval		p-value
		Lower limit	Upper limit	
Without any adjustments	1.27	0.73	2.21	0.392
Adjusting for gender of young person	1.48	0.84	2.58	0.173
Adjusting for time from randomisation to 24-month follow-up	1.35	0.77	2.37	0.298
Adjusting for intervention receipt (binary definition)	1.50	0.80	2.79	0.203
Adjusting for intervention receipt (number of weeks)	1.06	0.97	1.16	0.196
Analysis	Proportional odds ratio	95% Confidence Interval		p-value
		Lower limit	Upper limit	
As an ordinal outcome (adjusted)	1.42	0.82	2.46	0.211
As an ordinal outcome (without any adjustments)	1.26	0.71	2.25	0.429

Primary outcomes – subgroup analysis

An association was found between age of young person at baseline and reported alcohol use at 24-month follow-up (1.82 [1.23-2.71]), but there was no evidence of any differential effect by trial arms (1.30 [0.85-1.98]). In terms of gender of young person, the odds ratios for main effects and subgroup interaction terms suggested that the intervention worked differently for males and females. However, both confidence intervals included one, which suggests that there was no evidence of a differential treatment effect by gender of young person. Additionally, the main effect of young person who reported smoked at baseline (2.08 [1.06-4.11]) and having at least one parent/carer categorised at high-risk from problematic drinking (1.93 [1.01-3.69]) were significantly associated with the alcohol use outcome, but there were no discernible differences between arms. Other planned subgroup analyses provided no evidence of the SFP group having a differential effect.

An association was found between young person who reported smoked at baseline and reported drunkenness at 24-month follow-up (5.73 [2.07-15.90]), but there was no evidence of any differential effect by trial arms (0.57 [0.17-1.94]). In terms of the average young person GCM score (General Child Management), a one-point increase on the GCM score was associated with a 59% decrease in the odds of being classed as a young person who had been drunk in the last 30 days (0.41 [0.18-0.96]) a (higher GCM score indicates greater levels of parent management). However, there was no evidence of any differential effect by trial arm (1.27 [0.45-3.56]). For

gender of young person, the odds ratio for main effect showed a 17% increase for females who reported having been drunk in the previous 30 days at 24-month follow up compared to males, and the subgroup interaction indicated an 8% increase for females allocated to the SFP group compared to males and those allocated to the control group. However, both confidence intervals included one, which suggests that there was no evidence of a differential treatment effect by gender of young person. Overall, none of the subgroup analyses for the primary drunkenness outcome proved a significant association, which suggests that there was no evidence of a differential treatment effect.

For peer review only

Table S5: Subgroup analyses for the primary alcohol use outcome

Subgroup	Main effect odds ratio	95% Confidence Interval		p-value	Subgroup x SFP interaction	95% Confidence Interval		p-value
		Lower limit	Upper limit			Lower limit	Upper limit	
Age of young person at baseline	1.82	1.23	2.71	0.003	1.30	0.85	1.98	0.228
Gender of young person (female)	0.85	0.44	1.57	0.574	1.48	0.65	3.57	0.337
Young person had smoked at baseline	2.08	1.06	4.11	0.034	0.79	0.32	1.91	0.596
Having at least one parent/carer who is at high risk from problematic drinking	1.93	1.01	3.69	0.048	0.79	0.33	1.87	0.593
Baseline Family Affluence Scale (Medium)	1.13	0.44	2.86	0.750	1.08	0.31	3.73	0.589
Baseline Family Affluence Scale (High)	1.09	0.39	3.00		1.75	0.46	6.70	
Occupation Status (At least one parent in the family in part-time (but none in full-time) employment)	0.60	0.24	1.53	0.448	0.86	0.24	3.09	0.720
At least one parent in the family in full-time employment	0.91	0.41	2.02		1.46	0.50	4.25	
Young person belonging to a "family with challenges"	0.99	0.52	1.91	0.984	0.51	0.21	1.24	0.140
Strengths and difficulties score at baseline	1.02	0.97	1.07	0.498	1.00	0.93	1.07	0.929
Cohesion domain of the Family Relationship Index at baseline	1.01	0.99	1.03	0.328	0.99	0.96	1.01	0.273
Expressiveness domain of the Family Relationship Index at baseline	1.01	0.99	1.04	0.339	0.99	0.96	1.03	0.760
Conflict domain of the Family Relationship Index at baseline	1.02	0.99	1.05	0.211	1.00	0.97	1.04	0.871
Average Young person General Child Management score	1.01	0.54	1.91	0.968	0.45	0.18	1.12	0.086
Average Parent/carer General Child Management score	1.54	0.76	3.15	0.233	0.67	0.28	1.64	0.383

For peer review only

Table S6: Subgroup analyses for the primary drunkenness outcome

Subgroup	Main effect odds ratio	95% Confidence Interval		p-value	Subgroup x SFP interaction	95% Confidence Interval		p-value
		Lower limit	Upper limit			Lower limit	Upper limit	
Age of young person at baseline	2.22	0.98	5.04	0.056	1.30	0.67	2.53	0.435
Gender of young person (female)	1.17	0.49	2.80	0.690	1.08	0.35	3.29	0.920
Young person had smoked at baseline	5.73	2.07	15.90	0.001	0.57	0.17	1.94	0.369
Having at least one parent/carer who is at high risk from problematic drinking	1.40	0.58	3.38	0.448	1.68	0.53	5.26	0.376
Baseline Family Affluence Scale (Medium)	1.26	0.36	4.43	0.336	2.57	0.46	14.45	0.563
Baseline Family Affluence Scale (High)	0.99	0.25	3.88		2.18	0.34	14.15	
Occupation Status (At least one parent in the family in part-time (but none in full-time) employment)	1.31	0.46	3.73	0.673	0.76	0.17	3.33	0.550
At least one parent in the family in full-time employment	0.76	0.25	2.30		1.87	0.47	7.43	
Young person belonging to a "family with challenges"	1.19	0.50	2.85	0.692	0.41	0.13	1.30	0.131
Strengths and difficulties score at baseline	0.97	0.90	1.04	0.366	1.03	0.94	1.13	0.570
Cohesion domain of the Family Relationship Index at baseline	1.01	0.98	1.03	0.578	0.99	0.96	1.03	0.730
Expressiveness domain of the Family Relationship Index at baseline	0.99	0.96	1.02	0.516	1.01	0.97	1.05	0.661
Conflict domain of the Family Relationship Index at baseline	1.00	0.97	1.04	0.791	0.99	0.94	1.03	0.584
Average Young person General Child Management score	0.41	0.18	0.96	0.040	1.27	0.45	3.56	0.649
Average Parent/carer General Child Management score	1.13	0.43	2.98	0.804	0.77	0.24	2.46	0.656

Weekly smoking validated by salivary cotinine measures

Overall, 500 young people (53.7% of those randomised, 66.1% of those who provided 24-month follow-up interview data) provided saliva cotinine samples and self-reported weekly smoking behaviour at the 24-month follow-up interview. To identify weekly smokers using saliva cotinine, a cut-off of 15ng/mL was used[10,24]. Comparing cotinine samples with self-reported weekly smoking, 467 young people provided self-reported smoking data that agreed with their cotinine samples (93.4%). A slightly higher percentage of young people in

For peer review only

the SFP group reported being a non-weekly smoker and had cotinine samples ≤ 15 ng/mL and less than those who reported that they were a weekly smoker but had cotinine levels of ≤ 15 ng/mL.

Table S7: Agreement between self-reported weekly smoking behaviour and saliva cotinine

Self-report and saliva cotinine agreement		Control		SFP		Total	
		n	%	n	%	n	%
Concordant	Agree non-weekly smoker	176	78.6	227	82.2	403	80.6
	Agree weekly smoker	30	13.4	34	12.3	64	12.8
Discordant	Self-reported non-weekly smoker and cotinine > 15 ng/mL	13	5.8	9	3.3	22	4.4
	Self-reported weekly smoker and cotinine ≤ 15 ng/mL	5	2.2	6	2.2	11	2.2
Total		224	100.0	276	100.0	500	100.0

For peer review only

References

1. Goodman R. The Strengths and Difficulties Questionnaire: A Research Note. *Journal of Child Psychology and Psychiatry* 1997; **38**(5): 581-6.

2. McMahon R, Metzler C. Selecting parenting measures for assessing family based prevention interventions. In: Ashery R, Robertson E, Kumpfer K, Rockville M, eds. Drug abuse prevention through family interventions: National Institute on Drug Abuse; 1998: 294-323.

3. Spoth R, Redmond C, Haggerty K, Ward T. A Controlled Parenting Skills Outcome Study Examining Individual Difference and Attendance Effects. 1995; **57**(2): 449.

4. Spoth R, Redmond C, Shin C. Direct and indirect latent-variable parenting outcomes of two universal family-focused preventive interventions: extending a public health-oriented research base. *Journal of consulting and clinical psychology* 1998; **66**(2): 385-99.

5. Bandura A. A Guide for Constructing Self-Efficacy Scales. In: Pajares F, Urdan T, Greenwich, CT, eds. Self-Efficacy Beliefs of Adolescents: Information Age Publishing; 2006: 307-37.

6. Kerns K, Aspelmeier J, Gentzler A, Grabill C. Parent–child attachment and monitoring in middle childhood. *Journal of Family Psychology* 2001; **15**: 69-81.

7. Arthur M, Hawkins J, Catalano R, Pollard J. Student Survey of Risk and Protective Factors and Prevalence of Alcohol, Tobacco, & Other Drug Use. University of Washington: Social Development Research Group.

8. Hawkins JD, Guo J, Hill KG, Battin-Pearson S, Abbott RD. Long-Term Effects of the Seattle Social Development Intervention on School Bonding Trajectories. *Applied Developmental Science* 2001; **5**(4): 225-36.

9. Spoth RL, Redmond C, Trudeau L, Shin C. Longitudinal substance initiation outcomes for a universal preventive intervention combining family and school programs. *Psychology of Addictive Behaviors* 2002; **16**(2): 129-34.

10. Campbell R, Starkey F, Holliday J, et al. An informal school-based peer-led intervention for smoking prevention in adolescence (ASSIST): a cluster randomised trial. *The Lancet* 2008; **371**(9624): 1595-602.

11. National Centre for Social Research NFfE, Research. Smoking, Drinking and Drug Use Among Young People in England

in 2008 Full Report: NHS Information Centre for Health and Social Care, 2009.

12. Hibell B, Guttormsson U, Ahlström S, et al. The 2011 ESPAD Report: Substance Use Among Students in 36 European Countries: The Swedish Council for Information on Alcohol and other Drugs (CAN), 2011.

13. Rabin R, Charro FD. EQ-SD: a measure of health status from the EuroQol Group. *Annals of Medicine* 2001; **33**(5): 337-43.

14. The Kidscreen Group Europe. The KIDSCREEN Questionnaires: Quality of life questionnaires for children and adolescents: Handbook. Lengerich: Pabst Science Publishers, 2006.

15. Holahan CJ, Moos RH. Social support and adjustment: Predictive benefits of social climate indices. 1982; **10**(4): 403-15.

16. Billings AG, Moos RH. The role of coping responses and social resources in attenuating the stress of life events. *Journal of Behavioral Medicine* 1981; **4**(2): 139-57.

For peer review only

17. Spoth R, Goldberg C, Neppl T, Trudeau L, Ramisetty-Mikler S. Rural–urban differences in the distribution of parent-reported risk factors for substance use among young adolescents. *Journal of Substance Abuse* 2001; **13**(4): 609-23.
18. Arthur M, Hawkins J, Catalano R, Pollard J. Item-construct dictionary for the student survey of risk and protective factors and prevalence of alcohol, tobacco, and other drug use. Unpublished Technical Document. . Seattle, WA.: University of Washington, 1995.
19. Borland R, Yong HH, O'Connor RJ, Hyland A, Thompson ME. The reliability and predictive validity of the Heaviness of Smoking Index and its two components: Findings from the International Tobacco Control Four Country study. 2010; **12**(Supplement 1): S45-S50.
20. Bush K. The AUDIT Alcohol Consumption Questions (AUDIT-C)<subtitle>An Effective Brief Screening Test for Problem Drinking</subtitle>. *Archives of Internal Medicine* 1998; **158**(16): 1789.
21. Goldberg D. The Detection of Psychiatric Illness by Questionnaire. London: Oxford University Press; 1972.
22. Vieweg BW, Hedlund JL. The General Health Questionnaire (GHQ): A comprehensive review. *Journal of Operational Psychiatry* 1983; **14**(2): 74-81.
23. Ware JE, Sherbourne CD. The MOS 36-Item Short-Form Health Survey (SF-36). *Medical Care* 1992; **30**(6): 473-83.
24. Benowitz NL, Iii PJ, Ahijevych K, et al. Biochemical verification of tobacco use and cessation. *Nicotine & Tobacco Research* 2002; **4**(2): 149-59.

For peer review only

Checklist of items for reporting pragmatic trials

Section	Item	Standard CONSORT description	Extension for pragmatic trials	Location in the submitted paper
Title and abstract	1	How participants were allocated to interventions (eg, “random allocation,” “randomised,” or “randomly assigned”)		p1 (title); p3 (abstract)
**Introduction**				
Background	2	Scientific background and explanation of rationale	Describe the health or health service problem that the intervention is intended to address and other interventions that may commonly be aimed at this problem	p4
**Methods**				
Participants	3	Eligibility criteria for participants; settings and locations where the data were collected	Eligibility criteria should be explicitly framed to show the degree to which they include	pp5-6

Section	Item	Standard CONSORT description	Extension for pragmatic trials	Location in the submitted paper
			typical participants and/or, where applicable, typical providers (eg, nurses), institutions (eg, hospitals), communities (or localities eg, towns) and settings of care (eg, different healthcare financing systems)	
Interventions	4	Precise details of the interventions intended for each group and how and when they were actually administered	Describe extra resources added to (or resources removed from) usual settings in order to implement intervention. Indicate if efforts were made to standardise the intervention or if the intervention and its delivery were allowed to vary between	P6, p13; see also TIDIeR checklist

For peer review only

Section	Item	Standard CONSORT description	Extension for pragmatic trials	Location in the submitted paper
			participants, practitioners, or study sites	
			Describe the comparator in similar detail to the intervention	p6
Objectives	5	Specific objectives and hypotheses		p4
Outcomes	6	Clearly defined primary and secondary outcome measures and, when applicable, any methods used to enhance the quality of measurements (eg, multiple observations, training of assessors)	Explain why the chosen outcomes and, when relevant, the length of follow-up are considered important to those who will use the results of the trial	pp6-7

For peer review only

Section	Item	Standard CONSORT description	Extension for pragmatic trials	Location in the submitted paper
Sample size	7	How sample size was determined; explanation of any interim analyses and stopping rules when applicable	If calculated using the smallest difference considered important by the target decision maker audience (the minimally important difference) then report where this difference was obtained	7
Randomisation—sequence generation	8	Method used to generate the random allocation sequence, including details of any restriction (eg, blocking, stratification)		6
Randomisation—allocation concealment	9	Method used to implement the random allocation sequence (eg, numbered containers or central telephone), clarifying whether the sequence was concealed until interventions were assigned		6

For peer review only

Section	Item	Standard CONSORT description	Extension for pragmatic trials	Location in the submitted paper
Randomisation—implementation	10	Who generated the allocation sequence, who enrolled participants, and who assigned participants to their groups		6
Blinding (masking)	11	Whether participants, those administering the interventions, and those assessing the outcomes were blinded to group assignment	If blinding was not done, or was not possible, explain why	6
Statistical methods	12	Statistical methods used to compare groups for primary outcomes; methods for additional analyses, such as subgroup analyses and adjusted analyses		7-8
**Results**				
Participant flow	13	Flow of participants through each stage (a diagram is strongly recommended)—specifically, for each group, report the numbers of participants randomly assigned,	The number of participants or units approached to take part in the trial, the number which were	Summarised on p8, with full details

For peer review only

Section	Item	Standard CONSORT description	Extension for pragmatic trials	Location in the submitted paper
		receiving intended treatment, completing the study protocol, and analysed for the primary outcome; describe deviations from planned study protocol, together with reasons	eligible, and reasons for non-participation should be reported	included in Figure 1
Recruitment	14	Dates defining the periods of recruitment and follow-up		p8
Baseline data	15	Baseline demographic and clinical characteristics of each group		p9
Numbers analysed	16	Number of participants (denominator) in each group included in each analysis and whether analysis was by "intention-to-treat"; state the results in absolute numbers when feasible (eg, 10/20, not 50%)		Number of participants included in analysis

For peer review only

Section	Item	Standard CONSORT description	Extension for pragmatic trials	Location in the submitted paper
				described on pages 9-12. Use of ITT described on p7.
Outcomes and estimation	17	For each primary and secondary outcome, a summary of results for each group and the estimated effect size and its precision (eg, 95% CI)		pp9-11
Ancillary analyses	18	Address multiplicity by reporting any other analyses performed, including subgroup analyses and adjusted analyses, indicating which are prespecified and which are exploratory		pp10-13

For peer review only

Section	Item	Standard CONSORT description	Extension for pragmatic trials	Location in the submitted paper
Adverse events	19	All important adverse events or side effects in each intervention group		p13
**Discussion**				
Interpretation	20	Interpretation of the results, taking into account study hypotheses, sources of potential bias or imprecision, and the dangers associated with multiplicity of analyses and outcomes		pp13-14
Generalisability	21	Generalisability (external validity) of the trial findings	Describe key aspects of the setting which determined the trial results. Discuss possible differences in other settings where clinical traditions, health service organisation, staffing, or resources may vary from those of the trial	pp13-14

For peer review only

Section	Item	Standard CONSORT description	Extension for pragmatic trials	Location in the submitted paper
Overall evidence	22	General interpretation of the results in the context of current evidence		pp13-14

Cite as: *Zwarenstein M, Treweek S, Gagnier JJ, Altman DG, Tunis S, Haynes B, Oxman AD, Moher D for the CONSORT and Pragmatic Trials in Healthcare (Practihc) group. Improving the reporting of pragmatic trials: an extension of the CONSORT statement. BMJ 2008; 337;a2390.*

For peer review only

BMJ Open

The effectiveness of the Strengthening Families Programme in the UK at preventing substance misuse in 10-14 year olds: a pragmatic randomised controlled trial

Journal:	*BMJ Open*
Manuscript ID	bmjopen-2021-049647.R1
Article Type:	Original research
Date Submitted by the Author:	16-Sep-2021

Complete List of Authors:	Segrott, Jeremy; Cardiff University, Centre for Trials Research; Cardiff University, DECIPHer Centre, Cardiff School of Social Sciences<br>Gillespie, David; Cardiff University, Centre for Trials Research<br>Lau, Mandy; Cardiff University, Centre for Trials Research<br>Holliday, Jo; University of Oxford, UK Biobank, Big Data Institute<br>Murphy, Simon; Cardiff University, DECIPHer Centre, Cardiff School of Social Sciences Cardiff, UK<br>Foxcroft, David; Oxford Brookes University UK, Department of Psychology, Health and Professional Development, Faculty of Health and Life Sciences<br>Hood, Kerenza; Cardiff University, Centre for Trials Research<br>Scourfield, Jonathan; Cardiff University, School of Social Sciences<br>Phillips, Ceri; Swansea University, Human and Health Sciences<br>Roberts, Zoe; Cardiff University, Centre for Medical Education, School of Medicine<br>Rothwell, Heather; Cardiff University, DECIPHer Centre, Cardiff School of Social Sciences<br>Hurlow, Claire; Swansea University, Swansea Trials Unit, Swansea University Medical School<br>Moore, L; University of Glasgow, MRC/CSO Social and Public Health Sciences Unit

**Primary Subject Heading**:	Public health
Secondary Subject Heading:	Public health
Keywords:	PUBLIC HEALTH, Substance misuse < PSYCHIATRY, Community child health < PAEDIATRICS

SCHOLARONE™
Manuscripts

I, the Submitting Author has the right to grant and does grant on behalf of all authors of the Work (as defined in the below author licence), an exclusive licence and/or a non-exclusive licence for contributions from authors who are: i) UK Crown employees; ii) where BMJ has agreed a CC-BY licence shall apply, and/or iii) in accordance with the terms applicable for US Federal Government officers or employees acting as part of their official duties; on a worldwide, perpetual, irrevocable, royalty-free basis to BMJ Publishing Group Ltd ("BMJ") its licensees and where the relevant Journal is co-owned by BMJ to the co-owners of the Journal, to publish the Work in this journal and any other BMJ products and to exploit all rights, as set out in our licence.

The Submitting Author accepts and understands that any supply made under these terms is made by BMJ to the Submitting Author unless you are acting as an employee on behalf of your employer or a postgraduate student of an affiliated institution which is paying any applicable article publishing charge ("APC") for Open Access articles. Where the Submitting Author wishes to make the Work available on an Open Access basis (and intends to pay the relevant APC), the terms of reuse of such Open Access shall be governed by a Creative Commons licence – details of these licences and which Creative Commons licence will apply to this Work are set out in our licence referred to above.

Other than as permitted in any relevant BMJ Author's Self Archiving Policies, I confirm this Work has not been accepted for publication elsewhere, is not being considered for publication elsewhere and does not duplicate material already published. I confirm all authors consent to publication of this Work and authorise the granting of this licence.

For peer review only

The effectiveness of the Strengthening Families Programme in the UK at preventing substance misuse in 10-14 year olds: a pragmatic randomised controlled trial

Jeremy Segrott (0000-0001-6215-0870), David Gillespie, Mandy Lau, Jo Holliday, Simon Murphy, David Foxcroft, Kerenza Hood, Jonathan Scourfield, Ceri Phillips, Zoe Roberts, Heather Rothwell, Claire Hurlow, Laurence Moore

[1, 2]Jeremy Segrott, Senior Lecturer, segrottj@cardiff.ac.uk
[1]David Gillespie, Senior Research Fellow, gillespied1@cardiff.ac.uk
[1]Mandy Lau, Research Associate, lautm@cardiff.ac.uk
[3]Jo Holliday, Health Data Group Lead, jo.holliday@ndph.ox.ac.uk
[1]Simon Murphy, Professor in Social Interventions and Health, Director DECIPHer and Lead for the Schools Health Research Network, murphys7@cardiff.ac.uk
[4]David Foxcroft, Professor of Community Psychology and Public Health, david.foxcroft@brookes.ac.uk
[1]Kerenza Hood, Director of the Centre for Trials Research, hoodk1@cardiff.ac.uk
[5]Jonathan Scourfield, Professor of Social Work, scourfield@cardiff.ac.uk
[6]Ceri Phillips, Professor Emeritus, c.j.phillips@swansea.ac.uk
[7]Zoe Roberts, Lecturer robertsze@cardiff.ac.uk
[2]Heather Rothwell, Retired
[8]Claire Hurlow, Study Manager, c.e.hurlow@swansea.ac.uk
[9]Laurence Moore, Director, MRC/CSO Social & Public Health Sciences Unit, Laurence.Moore@glasgow.ac.uk

[1]Centre for Trials Research, Cardiff University, Heath Park, Cardiff, UK. CF14 4YS
[2]DECIPHer Centre, Cardiff University, 1-3 Museum Place, Cardiff, UK. CF10 3BD.
[3]UK Biobank, Big Data Institute, University of Oxford, Li Ka Shing Centre for Health Information and Discovery, Old Road Campus, Roosevelt Drive, Oxford, UK. OX3 7BN
[4]Department of Psychology, Health and Professional Development, Faculty of Health and Life Sciences, Oxford Brookes University, Headington Campus, Oxford, UK. OX3 0BP.
[5]School of Social Sciences, Cardiff University, Glamorgan Building, King Edward VII Avenue, Cardiff, UK. CF10 3WT.
[6]College of Human and Health Sciences, Swansea University, Singleton Park, Swansea, UK. SA2 8PP.
[7]Centre for Medical Education, School of Medicine, Cardiff University, Heath Park, Cardiff, UK. CF14 4XN
[8]Swansea Trials Unit, Swansea University Medical School, Swansea University, Singleton Park, Swansea, UK. SA2 8PP.
[9]MRC/CSO Social & Public Health Sciences Unit, University of Glasgow, Berkeley Square, 99 Berkeley Street, Glasgow, UK. G3 7HR.

Correspondence to: Dr J Segrott, Centre for Trials Research, Cardiff University, 4th Floor, Neuadd Meirionnydd, Heath Park, Cardiff, CF14 4YS. Email: segrottj@cardiff.ac.uk

Word count for main text: 6048

For peer review only - http://bmjopen.bmj.com/site/about/guidelines.xhtml

Abstract

Objectives
The Strengthening Families Programme 10–14 (SFP10-14) is a USA-developed universal group-based intervention aiming to prevent substance misuse by strengthening protective factors within the family. This study evaluated a Proportionate Universal implementation of the adapted UK version (SFP10-14UK) which brought together families identified as likely/not likely to experience/present challenges within a group setting.

Design
Pragmatic cluster-randomised controlled effectiveness trial, with families as the unit of randomisation and embedded process and economic evaluations.

Setting
The study took place in seven counties of Wales, UK.

Participants
715 families (919 parents/carers, 931 young people) were randomised.

Interventions
Families randomised to the intervention arm received the Strengthening Families Programme 10–14 (SFP10-14) comprising 7 weekly sessions. Families in intervention and control arms received existing services as normal.

Outcome measures
Primary outcomes were the number of occasions young people reported drinking alcohol in the last 30 days; and drunkenness during the same period, dichotomised as 'never' and '1-2 times or more'. Secondary outcomes examined alcohol/tobacco/substance behaviours including: cannabis use; weekly smoking (validated by salivary cotinine measures); age of alcohol initiation; frequency of drinking >5 drinks in a row; frequency of different types of alcoholic drinks; alcohol-related problems. Retention: Primary analysis included 746 young people (80.1%) (alcohol consumption) and 732 young people (78.6%) (drunkenness).

Results
There was no evidence of statistically significant between-group differences 2 years post-randomisation for primary outcomes (young people's alcohol consumption in the last 30 days adjusted odds ratio=1.11, 95% CI: 0.72 to 1.71, p=0.646; drunkenness in the last 30 days adjusted odds ratio=1.46, 95% CI: 0.83 to 2.55, p=0.185). There were no statistically significant between-group differences for other substance use outcomes, or those relating to wellbeing/stress, and emotional/behavioural problems.

Conclusions
Previous evidence of effectiveness was not replicated. Findings highlight the importance of evaluating interventions when they are adapted for new settings.

Trial registration
Current Controlled Trials ISRCTN63550893.

Keywords
Substance misuse, prevention, RCT, Strengthening Families Programme 10-14(UK), SFP10-14UK, young people, parents, family.

For peer review only - http://bmjopen.bmj.com/site/about/guidelines.xhtml

Strengths and Limitations

- Both trial arms had good participant retention rates at two-year follow-up.
- Recruitment, intervention delivery systems, and implementation replicated how the intervention would be delivered outside a trial setting, thus maximising applicability of findings.
- There were good levels of agreement between self-reported smoking behaviour and cotinine levels in collected saliva samples.
- Most outcomes – including the primary outcomes relating to alcohol, could only be measured using self-report.

For peer review only

Title: The effectiveness of the Strengthening Families Programme in the UK at preventing substance misuse in 10-14 year olds: a pragmatic RCT

Introduction

Prevention of substance misuse among young people is a key public health priority internationally. Early initiation of alcohol consumption and drug use during adolescence is associated with short term negative impacts (e.g. accidental injury, educational attainment) and poorer health over the life course, including substance use disorders and mental health problems. Family-based risk and protective factors, encompassing parental modelling of alcohol use,[1] substance use rules and monitoring,[2] and parenting styles and family relationships, have been identified as important,[3] and influence a range of outcomes alongside substance use such as adolescent mental health.[4] This has led to considerable policy interest in developing prevention interventions targeting key parenting/family variables[5] and a need to develop the international evidence base.[6]

The Strengthening Families Programme 10–14 (SFP10-14) is a USA-developed universal group-based intervention that aims to prevent substance misuse in young people by strengthening protective factors within the family.[7]

Three main trials of SFP10-14 in the USA using a universal delivery model have been conducted – each led by the team responsible for intervention development at Iowa State University (ISU). These trials have demonstrated the programme's effectiveness in delaying the onset of alcohol use, reducing uptake of smoking, the incidence of harder drug use (methamphetamine)[8 9] (6.5 years past baseline) and substance use at 10 year follow-up.[10] However, findings from the trials conducted in the USA have been criticised in relation to data analysis and reporting practices, sample 'selection bias', and low rates of intervention attendance.[11 12] For example, Gorman's methodological critique of the ISU's Capable Families and Youth (CAFY) evaluation argues that:

> … there were no statistically significant results reported using 2-tailed tests for individual point-in-time measures of alcohol, cigarettes and marijuana use, or for the drunkenness measure, in the CaFaY evaluation. The five effects reported as statistically significant were based on 1-tailed tests of significance and one of these was for a measure unique to that publication and two were based on a highly irregular practice of using posttest data as the baseline[12] (p35).

A critique by the same author of the third ISU- led trial – PROSPER, notes that only 19% of young people allocated to the intervention attended at least one SFP10-14 session. Another critique of this study is that it fails to provide adequate details concerning rates of refusal by participants to provide data at 7.5 years follow-up, and uncertainty regarding how many of those in the intervention arm who did complete questionnaires had attended any SFP10-14 session. [11]

The current study evaluates the version of the intervention that was culturally adapted for the UK (SFP10-14UK). It seeks to address some of the key limitations of the ISU-led trials. Steps to achieve this include pre-specification of outcomes and analysis plans, prospective publication of a trial protocol, and comprehensive reporting of data on recruitment, retention and intervention attendance rates.

Universal interventions (provided without recourse to criteria relating to risk/need) may inadvertently perpetuate inequalities because of their sometimes low reach among those most at need.[13] Targeted interventions may also face difficulties in identifying and recruiting such groups, create barriers to engagement through stigmatisation of potential participants[14] and generate iatrogenic effects through reinforcement and normalisation of harmful norms.[15 16] Targeted interventions – which bring together families with high levels of need, may also present problems in relation to feasibility of delivering activities with fidelity.[17] A distinctive aspect of this trial is that the intervention was implemented using a Proportionate Universal (PU) approach – combining universal provision but with its intensity/scale varied according to need.[18] This aimed to address some of the limitations of both universal and targeted approaches described above and operationalise delivery of a universal intervention within an existing UK system mainly geared towards targeted interventions. Driven by a desire to address health and social inequalities, a Proportionate Universal approach provides services universally but "at a scale and intensity of action […] proportionate to the level of disadvantage", thus aiming to improve engagement with, and effectiveness among groups with higher needs [19] (p16). Universal provision is designed to achieve sustainability, secure political and financial support, and avoid stigmatisation [20]. Variation in the "scale and intensity" of interventions is applied to recruitment (greater effort/resources may be needed to engage certain social groups) and during programme implementation to meet differing needs, aiding participant retention. Thus, within a Proportionate Universal approach, targeting may comprise aspects of both *selectivism*

For peer review only - http://bmjopen.bmj.com/site/about/guidelines.xhtml

(identifying whether certain groups should be targeted and how) and *particularism* – how interventions can be tailored to meet differing needs[21].

The Proportionate Universalism approach adopted had two key components. First, at recruitment stage the intervention was provided on a universal basis but with greater efforts and resources dedicated to recruiting those families with higher level support needs. This recruitment strategy aimed to reach families with higher level needs whilst avoiding creating barriers to engagement, and the limitations of a purely targeted approach in identifying such families.

Second, groups of families brought together for the intervention were composed of approximately 30% who were likely to experience or present challenges within a group setting - e.g. young people not attending or excluded from school/class/year group or working with behavioural support teams; Autistic Spectrum Condition; aggression/anger; low literacy skills; learning difficulties; mental health issues; English/Welsh not being a family's first language; and substance misuse (parent or young person), and 70% without such challenges. In line with a Proportionate Universal approach, this group composition was designed to ensure that there were sufficient numbers of staff to provide additional support to families who might experience or present challenges within a group setting. It was also designed to enable delivery of activities as intended within a highly manualised and structured intervention, and to promote prosocial group dynamics, with the goal of facilitating behaviour change processes hypothesized to generate the intervention's intended impacts.

Aim

The trial's primary objective was to assess the effectiveness of the SFP 10-14 UK in preventing alcohol misuse in young people. Its secondary objectives were to assess the programme's impact on drug misuse, smoking behaviour, alcohol initiation and drink-related problems and school performance, among young people. Tertiary objectives were to measure the extent to which SFP 10-14 UK had effects on young people's mental health and wellbeing, and protective factors for alcohol and tobacco use/misuse (e.g. family functioning, parenting and young people's peer resistance skills). The trial encompassed a cost-consequences analysis to assess whether the intervention was cost effective. It assessed if there were important variations in delivery and receipt across trial sites, and identified key programme theory, content, and processes.

Methods

Study design

The trial comprised a pragmatic cluster randomised controlled effectiveness trial with families as the unit of randomisation, and all individuals within a family recruited into the study allocated to the same arm. There were embedded process and economic evaluations. The study took place in seven counties of Wales, UK. Ethical approval for the trial was given by the Research Ethics Committee for Wales (reference 09/MRE09/53). Full details of the design, methods and outcomes have been published in the study protocol.[22] Programme delivery systems – including staffing arrangements and participant eligibility were designed to mirror future 'real world' implementation outside of a research context to maximise generalisability of findings. The researchers did not impose any additional standardisation of intervention delivery. Local authorities and third sector organisations with responsibility for supporting families or substance misuse prevention led multiple agency partnerships that delivered the programme in community settings. The trial was prospectively registered with ISCTRN registry on 3rd December 2009, and prior to any participants being enrolled.

Intervention

Drawing on theoretical models relating to bio-psychosocial vulnerability, youth resiliency and family process, SFP10-14 addresses risk and protective factors within the family environment known to shape later substance use behaviours [23]. It comprises 7 weekly sessions each lasting two hours. In the first hour parallel groups of young people and parents/carers from up to 12 families develop their understanding and skills, led by trained facilitators. Each session is guided by a facilitator manual which details activities and how they are to be delivered. In the second hour parents/carers and young people come together in family units to practise key skills. The programme has been culturally adapted for the UK (SFP10-14UK). Adaptations comprised changes to language and terms to fit with a UK context, and production of revised videos which used 'real life' situations filmed in home settings instead of studio-based narration. Core content and function of the intervention were retained and not subjected to significant change. [24]A logic model for the culturally adapted intervention has been developed by the authors[25].

Participants

For peer review only - http://bmjopen.bmj.com/site/about/guidelines.xhtml

Each programme was open to families with young people aged 10–14 from a large geographical area (e.g. town or rural district). Self-referrals to the Programme came forward in response to awareness-raising in community and educational settings. This included distribution of fliers to parents across relevant school year groups, attendance at community events, promotion via social media and placing of leaflets in local venues such as leisure centres. Other referrals came from agencies such as education, health and social services which identified families that may benefit from participating in the Programme. Potential referrers and families were informed that the Programme was being run as part of a trial and were provided with information about the trial. When a family was referred or applied to the SFP 10–14UK they were visited by a member of the programme delivery team who undertook a needs and eligibility assessment, which included exploration of any difficulties or support needs which participants might experience within a group setting. For example, this encompassed exploring the support needs of those with low literacy levels (as much of the programme involved text-based materials) or addressing how conflict within families might manifest when they came to work together on activities during sessions. Based on the information contained in the family referral/application form and the needs and eligibility assessment, the coordinator determined if eligible families were likely to experience or present challenges within a group setting. If families were deemed eligible to attend the programme they were asked if they were willing for a member of the research team to have access to their referral notes and to visit them. Where the family agreed, a research fieldworker visited them to obtain informed consent to participate in the trial and collect baseline data using a computer assisted personal interview (CAPI).

Any family deemed eligible to attend SFP 10–14 UK was included in the research trial, subject to their giving consent. Trial inclusion and exclusion criteria are listed in Table 1. Informed consent was obtained from all participants. For a family to be included in the research trial at least one parent/carer needed to consent to participate, and parent/carer consent for the inclusion of at least one young person in the trial was required. Young people also gave consent for their participation in the trial.

For peer review only

Table 1: Trial inclusion and exclusion criteria

Inclusion criteria	Exclusion criteria
Families in which at least one parent/carer and one young person are willing to attend the programme together	Families in which either a parent or young person does not want to attend the programme
Families with the ability to speak English (help can be provided for parents or young people with low literacy levels). Some programmes may also be delivered through the medium of Welsh if there is sufficient demand	Families in which there are either parents or young people who cannot speak English (or Welsh, where appropriate)
Families where a programme is being offered at a location to which it is practicable for a family to travel (as determined by the programme coordinator) within the next three months	Families where there is no programme being offered at a location to which it is practicable for a family to travel (as determined by the programme coordinator) within the next three months. In such a case the family would not be excluded. They will be placed on a waiting list for the programme and will be contacted when a programme is available. They will then be recruited into the trial
Families with a young person aged 10-14	-
-	Families who do not live together - e.g. the child/children are in care
-	Families with very high needs or challenges (such as serious substance misuse problems, family breakdown or crisis)

Randomisation and masking Procedures

Remote computerised randomisation was conducted by the research fieldworker at the end of the baseline interview once consent and baseline data had been obtained. Families were randomised within strata defined by the seven areas in which the study was conducted and using minimisation with a random element set at 80%. The minimisation algorithm aimed to achieve balance between randomised groups on family categorisation (family without challenge/family with challenges in a group setting), the average age of the young people within a family who were recruited into the trial (<12/12+), and the number of young people within a family who were recruited into the trial (1/>1). Families randomised to the intervention arm received SFP10-14UK in addition to usually provided services. Those randomised to the control continued to receive usually provided services only. These services included local authority parenting and family services, school-based support, and other providers' services such as substance misuse prevention teams, child, and adolescent mental health (CAMHS) and programmes operated by charities. There was some variation in the provision and organisation of services across the trial areas. Data on families' health, social care, education, and criminal justice service utilisation was collected from parents/carers at 9-, 15- and 24-month follow-up points. Due to the nature of the intervention, it was not possible for participants or those delivering the intervention to be blinded to families' group assignment. Assessment of trial outcomes was done blind to group assignment.

Outcomes

The trial had two primary outcomes - the number of occasions that young people reported having drunk alcohol in the last 30 days; and drunkenness during the last 30 days, dichotomised as 'never' and '1-2 times or more'.

Secondary outcomes were: reported use of cannabis (ever vs. never); weekly smoking (yes vs no, validated by salivary cotinine measures); age of alcohol use initiation; frequency of drinking more than 5 drinks in a row in the last 30 days; frequency of different types of alcoholic drinks; alcohol-related problems; and General Certificate of Secondary Education (GCSE) performance at age 15/16 (number of GCSEs passed and grades achieved, measured as a continuous outcome). All primary and secondary outcomes were collected from children at 2-year follow-up, with the exception of GCSE results, which it was proposed to collect via the Secure Anonymised Information Linkage (SAIL) Databank[26], once all participants had completed GCSEs/left compulsory education.

The trial's tertiary outcomes were: parenting (General Child Management Scale child report); family functioning (Family Relationship Index); children's wellbeing and stress (Strengths and Difficulties Questionnaire scores [SDQ]); children's health status (SF-36); parents/carers' health status (General Health Questionnaire - GHQ and EQ5D); costs and an assessment of relative cost-effectiveness, derived from a cost-consequences analysis; children's smoking status (i.e. whether they have ever smoked/smoked now); young

For peer review only - http://bmjopen.bmj.com/site/about/guidelines.xhtml

people's self-efficacy; age of first cigarette; and age of first drug use. Tables S1-S2 (Supplementary material) provide a description of the trial's primary, secondary and tertiary outcomes, and the specific measures used at main follow-up at 24 months.

Selection of outcomes and length of follow-up were driven by the intervention's hypothesized mechanisms of action and impacts, including long term substance use prevention, and short/medium term mediators, particularly family relationships and parenting.

Procedures were put in place to assess and respond to reports of adverse events. Serious Adverse Events (SAEs) were reviewed by a senior member of the trial team within 24 hours to assess the nature of the SAE, its seriousness, causality, and expectedness. Where an SAE was both related (resulting from administration of any of the research procedures) and unexpected the Trial Manager notified the REC within 15 days of receiving notification of the SAE.

Cost-consequences analysis
The costs of the programme were identified, measured and valued in monetary terms and combined with changes in resource utilisation of services as a result of programme participation. Distinctions were made between the costs incurred in each programme area to assess variation and potential for efficiency gains. The costs of delivering the programmes were derived from conversations with relevant colleagues and comprised staff time (plus training costs), venue and equipment costs, provision of support facilities, promotional materials and other resources involved in patient recruitment, together with costs incurred by participants. The cost per participant and cost per family were computed for the study and for each of the areas of implementation. The extent to which participation resulted in changes in utilisation of services was assessed by undertaking a series of participant interviews. Changes to resource utilisation were translated into monetary terms using appropriate published unit cost data and merged with the costs of programme delivery to generate the net cost of the programme, net cost per family, net cost per adult and net cost per young person. The net cost of delivering the programme was used alongside differences in primary, secondary and tertiary outcomes to generate a set of ratios of relative cost-effectiveness within the study period. Sensitivity analyses were undertaken to assess the extent to which changes in parameter estimates affected the baseline findings.

Statistical analysis
We aimed to randomise 756 families in total (378 per arm). Our target sample size provides 80% power with a two-sided alpha at 0.025 (halved to account for two primary outcomes) to detect either a 12 percentage point difference in young people reporting having drunk alcohol at the 24-month follow-up point (assuming a control group prevalence of 48%)[27] or a 10 percentage point difference in young people reporting having been drunk at the 24-month follow-up point (assuming a control group prevalence of 22%)[27]. The sample size was inflated based on the average family including 1.25 young people in the trial, an intracluster correlation coefficient of 0.2, and 25% loss to follow-up.

Descriptive statistics were calculated using means (standard deviation), medians (interquartile range) and percentages as appropriate.

The primary analyses were conducted on a modified intention to treat population (participants who provided outcome data analysed in the arm to which they were randomised) and involved fitting two-level logistic regression models to whether or not young people reported drinking alcohol/being drunk in the 30 days prior to their 24-month interview, with responses from young people nested within families. The models controlled for the variables balanced on at randomisation (other than number of young people, as this was balanced on to ensure equal number of young people per groups, rather than scientific reasons), the study area, and the baseline-reported alcohol use/drunkenness (depending on the outcome). To test the strength of the findings of the primary analyses, the models were re-analysed controlling for gender, time from randomisation to 24-month follow-up, and without controlling for any covariates (pre-planned analyses). The primary outcomes were also analysed as ordinal variables (i.e. the number of occasions in the last 30 days that young people reported drinking alcohol/being drunk). The three categories used in the analysis were 'no occasions', '1-2 occasions' and '3 or more occasions'. Two-level ordinal regression models were fitted to both outcomes, with and without adjusting for variables balanced on at randomisation and baseline alcohol use/drunkenness.

In order to assess the impact that intervention non-receipt had on the findings of the primary analyses, double logistic structural mean models using generalised method of moments were fitted to each primary outcome.[28] A binary definition of intervention receipt was agreed by the study team a priori which involved participants

For peer review only - http://bmjopen.bmj.com/site/about/guidelines.xhtml

attending at least five sessions, without missing more than one session in a row. The quantitative measure of intervention receipt was also used (i.e. the number of weeks attended).

Multiple imputation was used in a sensitivity analysis to assess the impact of missing primary outcome data on the conclusions drawn. Additional sensitivity analyses were also performed using more extreme assumptions about missing primary outcome data. For further details, see the Supplementary material.

Secondary outcomes were analysed by fitting two-level logistic regression models (proportion of young people at 24 months classing themselves as weekly smokers, reporting having ever used cannabis, having used cannabis in the previous 12 months, having used cannabis in the previous 30 days, and having experienced at least one of the listed alcohol-related problems in the previous 12 months) and two-level Cox proportional hazards models (time to initiation of alcohol, smoking, and drugs – in those participants who had not initiated at baseline). Covariate adjustment for secondary analyses mirrored that used for the primary analyses.

Potential modifiers of the intervention with respect to both primary outcomes were investigated by fitting interaction terms to trial arm and pre-planned subgroups (age, gender, and smoking behaviour of the young person, drinking behaviour of the parents/carers, socioeconomic status assessed using the Family Affluence Scale (low, medium or high) and highest occupational status of parents/carers (categories based on the NS-SEC self-coded method), family categorisation (family likely to experience challenges in a group setting / family without challenges in a group setting), young person reported SDQ, domains of the Family Relationship Index, and averaged young person and parent/carer scores of General Child Management scale at baseline).

As it was one of the distinguishing features of this trial, further exploratory post-hoc subgroup analyses were conducted to investigate potential differential intervention effects by family categorisation for the smoking, cannabis use, and SDQ outcomes, the latter being included because of its focus on mental health, conduct problems, and peer influences.

All analyses were conducted using STATA version 13.0.

Role of funder
The study funder was not involved in the design of the study, or collection, analysis, or interpretation of the data. The corresponding author had access to all data in the study and had final responsibility for the decision to submit for publication.

Patient and Public Involvement
Members of the public were involved in refining and piloting of outcome measures and study procedures. This included a mix of families who had already received SFP10-14 (and who were able to comment on the perceived relevance of proposed outcomes) and those without any prior knowledge of the intervention (and were therefore able to provide input on how trial participants with no previous knowledge might perceive study materials). Piloting comprised asking participants to complete baseline interviews with researchers, and then provide feedback on clarity of written information, time taken to complete the baseline interview, and the acceptability and appropriateness of outcome measures. This information was then used to refine the final selection of outcome measures and format of information sheets, etc. Input was also sought from a young people's research advisory group (ALPHA), led by a public involvement officer embedded within the study team. The public involvement officer formed stakeholder groups – formed of trial participants, to advise the study team at key points on recruitment and retention strategies, maintaining contact with the trial cohort, and plans for disseminating trial findings.

Results
Participant recruitment took place between 8th February 2010 and 18th June 2012. Seven hundred and fifteen families were recruited into the trial. This comprised 918 parents/carers and 931 young people. The number of participants completing questionnaires at each assessment point is included in the trial profile (Figure 1). Primary outcome data was collected from young people. The primary analyses included 746 young people for the alcohol use outcome at two-years post-randomisation (80.1%) and 732 for the drunkenness outcome at two-years post-randomisation (78.6%). Completion rates were similar across trial arms. Analysis of levels of service utilisation data at 9, 15 and 24 month follow up did not identify any discernible differences between the intervention and control arms.

For peer review only - http://bmjopen.bmj.com/site/about/guidelines.xhtml

Baseline characteristics

Table 2 illustrates the characteristics of parents/carers and young people at baseline in control and intervention groups. Randomised groups were broadly similar at baseline, with small differences noted for young person gender and parent/carers' reported cigarette use.

Table 2: Baseline characteristics for Parent/carers and young people according to experimental group

Parent/carer baseline characteristics		Control (n=457)	SFP (Intervention) (n=461)
Age (Median, IQR)		37.0 (32.0 to 43.0)	37.0 (33.0 to 42.5)
Gender	Female	105 (23.0%)	103 (22.3%)
	Male	352 (77.0%)	358 (77.7%)
Ethnic group	White	451 (98.7%)	458 (99.6%)
	Non-white	6 (1.3%)	2 (0.4%)
Relationship status	Single, not in a relationship	54 (12.0%)	68 (14.8%)
	Single, in a relationship	97 (21.5%)	91 (19.8%)
	Married/ civil partnership	200 (44.3%)	183 (39.9%)
	Separated/ divorced	95 (21.1%)	113 (24.6%)
	Widowed	4 (0.9%)	3 (0.7%)
	Other	1 (0.2%)	1 (0.2%)
Ever smoked a cigarette	Never	231 (50.5%)	207 (44.9%)
	Yes, at least once	226 (49.5%)	254 (55.1%)
Ever drunk alcohol	Never	85 (18.6%)	90 (19.5%)
	Yes, at least once	372 (81.4%)	371 (80.5%)
AUDIT-C	Low risk	304 (66.5%)	294 (64.6%)
	High risk	153 (33.5%)	161 (35.4%)
Ever taken drug	Never	300 (65.6%)	307 (66.6%)
	Yes, at least once	157 (34.4%)	154 (33.4%)
Deprivation overall score (Median, IQR)		27.5 (18.5 to 41.2)	29.4 (18.0 to 44.5)
Young people baseline characteristics		Control (n=454)	SFP (n=477)
	Age (Median, IQR)	11.5 (10.0 to 13.0)	12.0 (11.0 to 13.0)
Gender	Female	231 (50.9%)	269 (56.4%)
	Male	223 (49.1%)	208 (43.6%)
Ethnic group	White	446 (98.5%)	474 (99.6%)
	Non-white	7 (1.5%)	2 (0.4%)
Family affluence scale	Low (0-2)	83 (18.4%)	99 (20.9%)
	Medium (3-5)	234 (52.0%)	231 (48.8%)
	High (6-9)	133 (29.6%)	143 (30.2%)
Ever smoked a cigarette	Never	330 (72.7%)	348 (73.0%)
	Yes, at least once	124 (27.3%)	129 (27.0%)
Ever drunk alcohol	Never	314 (69.3%)	326 (68.5%)
	Yes, at least once	139 (30.7%)	150 (31.5%)
Ever taken drug	Never	433 (95.4%)	450 (94.3%)
	Yes, at least once	21 (4.6%)	27 (5.7%)
Child-deprivation overall score (Median, IQR)		28.1 (19.7 to 39.2)	28.2 (18.3 to 39.6)

Primary analysis

Table 3 shows the results of alcohol use and drunkenness in the previous 30 days prior to their 24-month follow-up interview for young people. Young people allocated to the SFP group had higher alcohol use and drunkenness prevalence than those allocated to the control group (26.4% versus 24.6% for alcohol use, 10.2% versus 8.3% for drunkenness). However, results from the multilevel models indicated insufficient evidence to reject the null hypothesis of no difference between groups (alcohol use adjusted odds ratio 1.11 [95% CI 0.72 to 1.71], p = 0.646; drunkenness AOR = 1.46 [95% CI 0.83 to 2.55], p = 0.185). Conclusions were unaffected by pre-planned secondary analyses of the primary outcomes (see Supplementary material – tables S3-4).

For peer review only - http://bmjopen.bmj.com/site/about/guidelines.xhtml

Table 3: Primary alcohol use and drunkenness outcomes (24-month follow-up)

Outcome	ICC*	Proportion of events in control arm (%)	Proportion of events in SFP arm (%)	Adjusted odds ratio†	95% Confidence Interval		p-value
					Lower limit	Upper limit	
Alcohol use in previous 30 days‡	0.25	85/345 (24.6)	106/401 (26.4)	1.11	0.72	1.71	0.646
Drunkenness in previous 30 days§	0.18	28/338 (8.3)	40/394 (10.2)	1.46	0.83	2.55	0.185

**Intracluster correlation coefficient: proportion of the total variance (in whether or not a young person reports having used alcohol/been drunk in the 30 days prior to their 24-month follow-up interview) attributable to differences between families. Calculated using the standard $\pi^2/3$ estimator for binary outcomes. †Analyses adjusted for variables that were balanced on at randomisation (study area, family categorisation and average age of young people within the family) and baseline alcohol use / drunkenness (corresponding with the outcome). ‡Number of occasions in the last 30 days that participant has drunk alcohol (asked at 24-month follow-up) – dichotomised to 0/1 or more occasions. § Number of occasions in the last 30 days that participant has been drunk (asked at 24-month follow-up) – dichotomised to 0/1 or more occasions.*

Subgroup analysis for primary outcomes

Tables S5 and S6 (Supplementary material) provide the results of the planned subgroup analysis for alcohol use and drunkenness in the previous 30 days. There was no evidence of any statistically significant differential intervention effects for any of the subgroups for either primary outcome.

Other substance use secondary outcomes

There was no evidence of statistically significant differences between groups for the other substance use outcomes (Table 4). Rates of agreement between self-reported weekly smoking behaviour and saliva cotinine are show in Table S7 (supplementary material).

For peer review only

For peer review only - http://bmjopen.bmj.com/site/about/guidelines.xhtml

Table 4: Other substance use secondary outcomes (24 month follow-up)

Outcome		ICC*	Proportion of events in control arm (%)	Proportion of events in SFP arm (%)	Adjusted odds ratio	95% Confidence Interval		p-value
						Lower limit	Upper limit	
Alcohol-related problems in the last 12 months		0.18	51/326 (15.6)	60/384 (15.6)	0.95	0.59	1.54	0.849
Currently smoke at least one cigarette a week		0.41	52/347 (15.0)	59/402 (14.7)	0.94	0.53	1.66	0.828
Ever taken cannabis			46/338 (13.6)	58/393 (14.8)	1.21	0.57	2.55	0.617
Taken cannabis at least once in the last 12 months		0.43	41/337 (12.2)	49/393 (12.5)	1.01	0.51	1.97	0.985
Taken cannabis at least once in the last 30 days			20/338 (5.9)	21/393 (5.3)	0.77	0.36	1.68	0.518
Time to alcohol initiation†			88/246 (35.8)	81/273 (29.7)	0.86	0.64	1.17	0.344
Frequency of consuming 5+ drinks in a row in the last 30 days‡	0	0.00	315/345 (91.3)	348/401 (86.8)	1.55	0.95	2.51	0.080
	1 to 2		15/345 (4.3)	36/401 (9.0)				
	3 to 5		15/345 (4.8)	17/401 (4.2)				
Frequency of drinking beer‡	Never	0.00	290/346 (83.8)	331/401 (82.5)	1.15	0.77	1.72	0.492
	Rarely		20/346 (5.8)	17/401 (4.2)				
	Every month		26/346 (7.5)	36/401 (9.0)				
	At least once a week		10/346 (2.9)	17/401 (4.2)				
Frequency of drinking wine‡	Never	0.00	323/345 (93.6)	375/402 (93.3)	1.06	0.59	1.92	0.837
	Rarely		5/345 (1.4)	7/402 (1.7)				
	At least once a month		17/345 (4.9)	20/402 (5.0)				
Frequency of drinking spirits‡	Never	0.71	298/346 (86.1)	351/401 (87.5)	0.85	0.50	1.46	0.567
	Rarely		15/346 (4.3)	13/401 (3.2)				
	Every month		21/346 (6.1)	24/401 (6.0)				
	At least once a week		12/346 (3.5)	13/401 (3.2)				
Frequency of drinking alcopops‡	Never	0.35	303/346 (87.6)	345/402 (85.8)	1.24	0.78	1.97	0.361
	Rarely		19/346 (5.5)	17/402 (4.2)				
	At least once a month		24/346 (6.9)	40/402 (10.0)				
Frequency of drinking cider‡	Never	0.00	305/346 (88.2)	346/402 (86.1)	1.26	0.80	1.97	0.313
	Rarely		12/346 (3.5)	13/402 (3.2)				
	At least once a month		29/346 (8.4)	43/402 (10.7)				

**Calculated using the standard $\pi^2/3$ estimator for binary outcomes. †Single level Cox regression; Percentage of young people who initiated alcohol after baseline; adjusted hazard ratio. ‡Multilevel ordinal regression - not consuming 5+ drinks in a row and never drunk anything alcoholic were the reference categories.*

For peer review only

For peer review only - http://bmjopen.bmj.com/site/about/guidelines.xhtml

GCSE Performance at age 15/16

Based on the analysis of the primary and secondary self-reported outcomes at 2-year follow-up, it was determined that collection of the GCSE data was not justified.

Post-hoc subgroup analysis

Table 5 provides the results of unplanned subgroup analysis aiming to explore differential intervention effects on the secondary substance use outcomes and SDQ score by whether or not the family was likely to experience/present challenges in a group setting. The motivation for this exploratory work stemmed from the subgroup findings for the two primary outcomes, which demonstrated small (not statistically significant) differential effects favouring families experiencing/presenting challenges within a group setting. The attrition rate for the 24-month data was similar between families with or without challenges in a group setting (17.1% for families with challenges versus 12.2% without). The pattern found for the primary outcomes (i.e. differential outcomes favouring families with challenges) was reflected across the additional outcomes investigated. Taking the 'cannabis' outcome as one example, we see that the odds of reporting cannabis use in the last 12 months are higher for the main effect (i.e. higher for those in a family with challenges and allocated to the control arm, compared to families without challenges in a group setting, allocated to the control arm), and the interaction term indicates a reversal of this effect for those in the SFP arm (i.e. cannabis use is higher for families without challenges allocated to the SFP arm than families with challenges allocated to the SFP arm).

Table 5: Post-hoc subgroup analyses for the secondary substance use outcomes and SDQ

Subgroup	Main effect adjusted odds ratio*	95% Confidence Interval		Subgroup x SFP interaction adjusted odds ratio	95% Confidence Interval	
		Lower limit	Upper limit		Lower limit	Upper limit
Currently smoke at least one cigarette a week	1.95	0.83	4.55	0.35	0.11	1.10
Ever taken cannabis	2.14	0.70	6.58	0.33	0.07	1.50
Taken cannabis at least once in the last 12 months	2.11	0.79	5.68	0.25	0.06	0.99
Taken cannabis at least once in the last 30 days	1.22	0.39	3.77	0.30	0.06	1.54
Subgroup	Adjusted mean difference	95% Confidence Interval		Subgroup x SFP interaction adjusted mean difference	95% Confidence Interval	
		Lower limit	Upper limit		Lower limit	Upper limit
Strengths and difficulties total difficulties score	1.77	0.61	2.93	-2.27	-3.83	-0.71

*The main effect can be interpreted as the effect of belonging to a family with challenges in a group setting (relative to a family without challenges in a group setting) for those allocated to the control arm.

Cost-consequences analysis results

The costs associated with the implementation of the intervention were £1,163,278, which equated to £20,773 per programme and £1,240 per participant, with wide variation in costs across programme areas. There was no difference in costs of healthcare utilisation between the groups at 9-, 15- and 24-month follow-up. At 9- and 15-month follow-up the main cost driver was that of primary care usage, while at 24-month follow-up the main driver was in-patient episodes. There was no difference between the groups for adult EQ-5D scores at baseline or 24 –month follow-up.

EQ-5D scores for adults at both baseline and 24 –month follow-up were very similar between the intervention and control arms. Conversely, the EQ-5D scores for the children in the intervention arm saw a difference in the scores between baseline and 24 –month follow-up (a mean increase of 0.043 per participant in the intervention arms, compared to 0.030 in the control).

For peer review only - http://bmjopen.bmj.com/site/about/guidelines.xhtml

A complete case analysis was conducted looking at the number of parents who answered the 9-, 15- and 24-month questionnaires and who also answered both the baseline and 24-month EQ-5D questionnaire (Intervention n=197; Control n=129). The mean EQ-5D difference in the control group was 0.053 at a cost difference of £6,271, compared to 0.007 and £7,175 in the intervention group. This resulted in a difference of -0.046 at a cost of £904 with an incremental cost effectiveness ratio (ICER) of -£19,652. Therefore the intervention arm was dominated.

As with the vast majority of outcomes, there was no difference in resource utilisation or associated costs between the trial arms at 9-, 15- and 24-month follow-up. This was the case for resource utilisation in general and specifically to family/parenting-based support services.

Process evaluation

Detailed findings from the process evaluation have been reported.[25] Of the 56 programmes run, 47 (84%) achieved the target group size (5-12 families) at enrolment. Facilitators rated participant engagement high in 94% of activities. There was no evidence that engagement differed by group size. Twenty-two (39%) of the 56 programmes achieved the target composition of families with challenges (30%) and those without challenges in a group setting (70%). However, most of the remaining programmes (39/56) achieved a mix of families which was in line with the intended group composition model (i.e. the majority of families were those without challenges in a group setting).

Overall implementation fidelity was good. Facilitators were asked to rate fidelity of the sessions they delivered using a checklist designed by the SFP10-14 developers. Data were available for 50 of 56 programmes, across which facilitators rated 96% of individual activities as mostly/fully covered – this varied across trial sites from 90-99%. To assess reliability of facilitator reports a sample of 47 sessions from the 50 programmes for which data were available were observed and scored by researcher observers using the same fidelity assessment sheet. Observers rated 77% of observed activities as mostly or fully covered (range 63-88%): ICC scores from 2 observers = 0.73 (95% CI=0.65-0.79). Facilitators' and observers' scores agreed 83% of the time (area range 73-93%).

Recruitment of families was a key implementation challenge. Practitioners reported that the Proportionate Universal approach heightened such challenges as the universal recruitment diverged from the focus of existing systems and referring agencies on families with higher level needs. However, facilitators identified the Proportionate Universal approach to group composition (with a mix of families with and without challenges within a group setting) as helpful in enabling activities to be delivered as intended, the cultivation of positive group dynamics, and the provision of support to participants with additional needs.

Adverse outcomes

There were no SAEs which were both related (resulting from administration of any of the research procedures) and unexpected.

Discussion

In this paper we have reported findings from a randomised controlled trial of the SFP10-14 following its adaptation for a UK context. We found no evidence of between-group differences 2 years past baseline for the primary outcomes (young people's alcohol consumption and drunkenness in the last 30 days) or for any of the other substance use outcomes. Similarly, there were no between-group differences in relation to young people's wellbeing, stress, and emotional and behavioural problems. Findings were unaltered for pre-planned subgroup analyses. Our study findings contrast with evaluations of SFP10-14 conducted in the USA in which long term effects on substance use outcomes have been detected[8 10]. However, the absence of intervention effects in the UK setting replicates those of more recent European trials in which adapted versions of SFP10-14 have been evaluated. In Sweden no effects on substance use or 'breaking behaviours' were detected[29] though significant adaptations made to the intervention may have undermined its hypothesized mechanisms of action[30]. Likewise, findings from the trial conducted in Poland showed no effects on the primary outcomes (substance use) or aspects of family functioning.[31] An RCT in Germany identified only limited evidence of effectiveness.[32] For young people, there were no differences in rates of substance use, except for tobacco initiation (but only among those followed up at 18 months).

Process evaluation findings indicated that SFP10-14UK was delivered with good fidelity (albeit facilitators' reports were biased towards overstating fidelity), suggesting that this trial was a fair assessment of SFP10-14 when delivered as intended. The Proportionate Universal approach adopted successfully recruited families with

For peer review only - http://bmjopen.bmj.com/site/about/guidelines.xhtml

differing levels of need (but without the potential stigmatisation generated by targeted interventions), and most groups achieved positive group dynamics in line with hypothesized behaviour change mechanisms.

Several strengths of this study should be noted. There were good participant retention rates and high rates of data completion across both trial arms at main follow-up. At 24-month follow-up there were good levels of agreement between self-report of smoking behaviour and cotinine levels in collected samples. This study was designed as a pragmatic trial in which recruitment, intervention delivery systems, and implementation were intended to replicate how the intervention was likely to be delivered outside a trial setting, thus maximising the applicability of our findings. We did not identify evidence of compensatory provision of services in the control arm, increasing our confidence that the study findings would have detected any intervention effects which were present.

In terms of limitations, by virtue of necessity, all outcomes (including the primary outcomes) were measured using self-report. However, self-report of smoking behaviours was validated using measures of salivary cotinine. The trial utilised multiple statistical tests given the large number of outcomes. All outcomes measured hypothesized impacts of SFP10-14UK and mapped on to the intervention's logic model. In line with similar previous studies of complex interventions[33], we pre-specified primary and secondary outcomes, and also pre-defined subgroup analysis, clearly indicating those subgroup-analyses which were post-hoc.

SFP10-14UK did not replicate the impacts on substance misuse prevention found in the American evaluations. There are a number of possible explanations for the null findings. The American trials tended to have longer-term follow-ups and impacts were sometimes stronger in the longer-term (e.g. over 5-10 years). However, the epidemiological trajectory of young people's drinking etc in the UK is earlier than in the US studies so we anticipated any effects to be apparent earlier. The intervention was delivered with good fidelity, and thus implementation failure is unlikely to explain the absence of impacts on hypothesized outcomes. Likewise, cultural adaptations made to SFP10-14 for delivery in the UK prior to this trial focused on aspects of language and presentation of videos, and the core content and functions of the intervention were retained. The intervention as evaluated in this trial is therefore comparable with the original version evaluated in the USA. Trials of SFP10-14 in the USA have been criticised for their approach to data analysis and presentation of outcomes, with some suggestion that these might have led to unfounded claims for intervention effectiveness. [11 12 34] In this trial we addressed these criticisms by pre-specifying trial outcomes (via a published protocol) and how these would be analysed (through a statistical analysis plan which was approved by an independent steering committee).

It is possible that 'usual care' (to which SFP10-14UK was added for the intervention group) may vary in important ways between the American context and the European settings into which the intervention has been introduced. We were not able to compare data on routine provision of health and welfare services across the current and previous trials. In the present study, SFP10-14UK was integrated into existing parenting, family support and substance misuse prevention provision. Such provision, and their take up by families, may have varied across settings in which trials of SFP10-14 have taken place. The relatively well developed suite of services which formed 'usual care' in the current study may have made it more difficult for the intervention to show significant effects.

Another possibility is that intervention content may have interacted in different ways with the context of families receiving it. Post-hoc subgroup analysis of substance use-related secondary outcomes appeared to show that for families with challenges in a group setting, differences between intervention and control group were in favour of the intervention group. For families without challenges in a group setting, this pattern was reversed. It is possible that the intervention activities better met the needs of those families identified as likely to experience or present challenges in a group setting, or that key learning had greater fit with their circumstances and was thus easier to adopt and integrate. Alternatively it may be that group dynamics and the interaction between participants played a role in shaping this patterning, with benefits for those with challenges to the detriment of those without. For example, Wiggins, et al. have previously noted that "some interventions targeting people at risk can expose participants to the influence of new peers who are more supportive of or more engaged in behaviours associated with risk, thereby spreading risk"[35]. However, no such differences according to families with/without challenges in a group setting were identified for our primary outcomes. Whilst some previous trials of SFP10-14 have identified potential patterning of intervention effects by sub groups according to baseline assessment of risk for later substance use[32 36 37], our findings here are not directly comparable, since we were concerned with experience/presentation of challenges within a group setting, and not general levels of support needs or risk of later substance use. Previous research on interventions which are targeted based on assessment of young people's risk behaviours has identified the potential for stigmatisation of

For peer review only - http://bmjopen.bmj.com/site/about/guidelines.xhtml

participants and generation of iatrogenic effects through reinforcement and normalisation of harmful norms.[15 16] The Proportionate Universal approach adopted by implementation of SFP10-14UK in this study appeared to succeed in reaching families with higher levels of need whilst avoiding the stigmatisation of participants encountered by targeted interventions. However, it is unclear whether the potential intervention effects for families with challenges and the potential harms for families without challenges may be driven by intervention 'fit', or at least derives partly from group dynamics and the ways in which participants interact.

This trial highlights the importance of assessing the effectiveness of interventions when they are adapted for and then implemented in new settings. Such assessment involves not only a-priori identifying whether an adapted intervention remains faithful to its original logic model, but also understanding how it interacts with the new context into which is introduced. This context includes both the existing provision of services, and the needs of the participants who receive it.

Author Contributions

JSe wrote the first draft of the paper with input from other authors and led the process evaluation.
DG led the design and conduct of statistical analysis and assisted with writing the first draft of the paper and revisions.
ML contributed to statistical analysis and the writing of the first draft of the paper and subsequent drafts
JH was a trial manager and collected and analysed data on recruitment and retention.
SM provided input on the design, conduct and analysis of data for the process evaluation.
DF advised on the design of the study, the selection of outcome measures and the interpretation of the results.
KH contributed to the overall design of the study the design of the trial's statistical analysis plan, and the analysis of trial data.
JSc provided input on data collection and analysis within the process evaluation.
CP designed and led the health economic evaluation.
ZR contributed to the design and conduct of statistical analysis.
HR conducted data collection and analysis for the process evaluation.
CH was a trial manager and contributed to data collection, collation of data on recruitment and retention.
LM was chief investigator and led the overall design and conduct of the study.

All authors contributors contributed to the writing of the paper.
Jeremy Segrott is responsible for the overall content as guarantor, accepts full responsibility for the work and the conduct of the study, had access to the data, and controlled the decision to publish. The corresponding author attests that all listed authors meet authorship criteria and that no others meeting the criteria have been omitted.

Competing interests

All authors have completed the ICMJE uniform disclosure form at www.icmje.org/coi_disclosure.pdf and declare financial support from the National Prevention Research Initiative (managed by the Medical Research Council), Welsh Government and Cardiff Strengthening Families Team. DF's institution has previously received financial support for the development of the SFP 10–14 UK programme materials from the alcohol industry; there are no other relationships or activities that could appear to have influenced the submitted work.

Ethics Approval

Ethical approval for the trial was given by the Research Ethics Committee for Wales (reference 09/MRE09/53).

Funding

Funding of £2.1 M from the National Prevention Research Initiative, managed by the Medical Research Council – award G0802128, included c.£650 k implementation costs. The NPRI funding partners are Alzheimer's Research Trust; Alzheimer's Society; Biotechnology and Biological Sciences Research Council; British Heart Foundation; Cancer Research UK; Chief Scientist Office, Scottish Government Health Directorate; Department of Health; Diabetes UK; Economic and Social Research Council; Engineering and Physical Sciences Research Council; Health & Social Care Research & Development Office for Northern Ireland; Medical Research Council; The Stroke Association; Welsh Government; and World Cancer Research Fund.

A representative from the study funders was a member of the trial's Independent Trial Steering Committee. The Welsh Government provided c.£675 k of partnership funding, to cover the cost of implementation in three trial areas, and the associated training and support provided by the Cardiff Strengthening Families Programme team. Further support from Welsh Government provided £208k to cover programme delivery in six trial sites from

For peer review only - http://bmjopen.bmj.com/site/about/guidelines.xhtml

August 2011-July 2012. The Cardiff Strengthening Families Programme team also provided financial support for programme delivery and trial recruitment in schools.

At the time of the study DECIPHer was a UKCRC Public Health Research Centre of Excellence. Funding from the British Heart Foundation, Cancer Research UK, Economic and Social Research Council (RES-590-28-0005), Medical Research Council, the Welsh Government and the Wellcome Trust (WT087640MA), under the auspices of the UK Clinical Research Collaboration, is gratefully acknowledged. DECIPHer funding has supported JSe and JH's input into the trial. The centre is now funded by Welsh Government through Health and Care Research Wales. DECIPHer funding has supported JSe and JH's input into the trial.

LM is supported by the Medical Research Council (MC_UU_12017/14) and Chief Scientist Office (SPHSU14).

The Centre for Trials Research is funded by Health & Care Research Wales and Cancer Research UK.

Acknowledgements
We would like to thank the parents/carers and young people who participated in the trial. Grateful thanks are also due to the delivery teams who implemented SFP10-14UK as part of the trial, the independent trial steering committee and data monitoring committee, and the Participant Resource Centre at Cardiff University.

Data sharing
Researchers can request access to de identified participant data by contacting the corresponding author. Requests for access to data will be subject to approval of a proposal, completion of a signed data access agreement, and compliance with institutional and legal requirements. The study protocol is freely available, and the statistical analysis plan is available upon request.

Transparency Declaration
The lead author (the manuscript's guarantor) affirms that the manuscript is an honest, accurate, and transparent account of the study being reported; that no important aspects of the study have been omitted; and that any discrepancies from the study as originally planned (and, if relevant, registered) have been explained.

Licence Statement
I Jeremy Segrott, the Submitting Author has the right to grant and does grant on behalf of all authors of the Work (as defined in the below author licence), an exclusive licence and/or a non-exclusive licence for contributions from authors who are: i) UK Crown employees; ii) where BMJ has agreed a CC-BY licence shall apply, and/or iii) in accordance with the terms applicable for US Federal Government officers or employees acting as part of their official duties; on a worldwide, perpetual, irrevocable, royalty-free basis to BMJ Publishing Group Ltd ("BMJ") its licensees and where the relevant Journal is co-owned by BMJ to the co-owners of the Journal, to publish the Work in BMJ Open and any other BMJ products and to exploit all rights, as set out in our licence.

The Submitting Author accepts and understands that any supply made under these terms is made by BMJ to the Submitting Author unless you are acting as an employee on behalf of your employer or a postgraduate student of an affiliated institution which is paying any applicable article publishing charge ("APC") for Open Access articles. Where the Submitting Author wishes to make the Work available on an Open Access basis (and intends to pay the relevant APC), the terms of reuse of such Open Access shall be governed by a Creative Commons licence – details of these licences and which Creative Commons licence will apply to this Work are set out in our licence referred to above.

List of figures
Figure 1: Trial consort participant flow chart.

References

1. Foxcroft DR, Lowe G. Adolescents' Alcohol use and Misuse: The socializing influence of perceived family life. *Drugs: Education, Prevention and Policy* 1997;4(3):215-29. doi: 10.3109/09687639709028544
2. Garmienė A, Žemaitienė N, Zaborskis A. Family time, parental behaviour model and the initiation of smoking and alcohol use by ten-year-old children: an epidemiological study in Kaunas, Lithuania. *BMC Public Health* 2006;6(1):287. doi: 10.1186/1471-2458-6-287
3. Velleman RDB, Templetonl LJ. Substance misuse by children and young people: the role of the family and implications for intervention and prevention. *Paediatrics and Child Health* 2007;17(1):25-30. doi: 10.1016/j.paed.2006.12.002
4. Oldfield J, Humphrey N, Hebron J. The role of parental and peer attachment relationships and school connectedness in predicting adolescent mental health outcomes. *Child and Adolescent Mental Health* 2016;21(1):21-29. doi: 10.1111/camh.12108
5. Cuijpers P. Three Decades of Drug Prevention Research. 2003;10(1):7-20. doi: 10.1080/0968763021000018900
6. Turner KMT, Sanders MR. Dissemination of evidence-based parenting and family support strategies: Learning from the Triple P—Positive Parenting Program system approach. *Aggression and Violent Behavior* 2006;11(2):176-93. doi: 10.1016/j.avb.2005.07.005
7. Molgaard VM, Spoth R, Redmond C. Competency training: The Strengthening Families Program for Parents and Youth 10 142000.
8. Spoth R, Redmond C, Shin C, et al. Brief Family Intervention Effects on Adolescent Substance Initiation: School-Level Growth Curve Analyses 6 Years Following Baseline. *Journal of Consulting and Clinical Psychology* 2004;72(3):535-42. doi: 10.1037/0022-006X.72.3.535
9. Spoth RL, Clair S, Shin C, et al. Long-term Effects of Universal Preventive Interventions on Methamphetamine Use Among Adolescents. *Archives of Pediatrics & Adolescent Medicine* 2006;160(9) doi: 10.1001/archpedi.160.9.876
10. Spoth RL, Trudeau LS, Guyll M, et al. Benefits of Universal Intervention Effects on a Youth Protective Shield 10 Years After Baseline. *Journal of Adolescent Health* 2012;50(4):414-17. doi: 10.1016/j.jadohealth.2011.06.010
11. Gorman DM. Letter to the Editor: Sources of bias and need for caution in interpreting the results of Spoth et al.'s (2017) PROSPER study. *Psychological Medicine* 2018;48(4):694-96. doi: 10.1017/s0033291717002355
12. Gorman DM. The decline effect in evaluations of the impact of the Strengthening Families Program for Youth 10-14 (SFP 10-14) on adolescent substance use. *Children and Youth Services Review* 2017;81:29-39. doi: 10.1016/j.childyouth.2017.07.009
13. Dryden R, Williams B, McCowan C, et al. What do we know about who does and does not attend general health checks? Findings from a narrative scoping review. *BMC Public Health* 2012;12(1):723. doi: 10.1186/1471-2458-12-723
14. Bayer JK, Hiscock H, Morton-Allen E, et al. Prevention of mental health problems: rationale for a universal approach. *Archives of Disease in Childhood* 2007;92(1):34-38.
15. Sorhaindo A, Bonell C, Fletcher A, et al. Being targeted: Young women's experience of being identified for a teenage pregnancy prevention programme. *Journal of Adolescence* 2016;49:181-90. doi: 10.1016/j.adolescence.2016.03.013
16. Dishion TJ, Tipsord JM. Peer Contagion in Child and Adolescent Social and Emotional Development. *Annual Review of Psychology* 2011;62(1):189-214. doi: 10.1146/annurev.psych.093008.100412

For peer review only - http://bmjopen.bmj.com/site/about/guidelines.xhtml

17. Segrott J. Recruitment and group composition strategies for family-based substance misuse prevention interventions: an exploratory evaluation. 2013;8(2):89-109. doi: 10.1108/jcs-03-2013-0007
18. Marmott Review. Marmot Review: Fair society, healthy lives: strategic review of health inequalities in England post 2010. London: Institute of Health Equity, 2010.
19. Marmot Review. Marmot Review: Fair society, healthy lives: strategic review of health inequalities in England post 2010. London: Institute of Health Equity, 2010.
20. UCL Institute of Health Equity. Review of social determinants and the health divide in the WHO European Region: final report. Copenhagen: WHO Regional Office for Europe, 2013.
21. Carey G, Crammond B, De Leeuw E. Towards health equity: a framework for the application of proportionate universalism. *International Journal for Equity in Health* 2015;14(1) doi: 10.1186/s12939-015-0207-6
22. Segrott J, Gillespie D, Holliday J, et al. Preventing substance misuse: study protocol for a randomised controlled trial of the Strengthening Families Programme 10–14 UK (SFP 10–14 UK). *BMC Public Health* 2014;14(1):49. doi: 10.1186/1471-2458-14-49
23. Molgaard VM, Spoth R, Redmond C. Competency training: the Strengthening Families Program for parents and youth 10–14. *OJJDP Juvenile Justice Bulletin* 2000;(NCJ 182208)
24. Allen D, Coombes L, Foxcroft DR. Cultural accommodation of the Strengthening Families Programme 10-14: UK Phase I study. *Health Education Research* 2006;22(4):547-60. doi: 10.1093/her/cyl122
25. Segrott J, Murphy S, Rothwell H, et al. An application of Extended Normalisation Process Theory in a randomised controlled trial of a complex social intervention: process evaluation of the Strengthening Families Programme (10-14) in Wales, UK. 2017 doi: 10.1016/j.ssmph.2017.01.002
26. Lyons RA, Jones KH, John G, et al. The SAIL databank: linking multiple health and social care datasets. *Bmc Medical Informatics and Decision Making* 2009;9 doi: 10.1186/1472-6947-9-3
27. Currie C, Zanotti C, Morgan A, et al. Social determinants of Health and Well-Being among Young People. Health Behaviour in School-Aged Children (HBSC) Study: International Report from the 2009/2010 Survey. Health Policy for Children and Adolescents. Copenhagen: WHO Regional Office for Europe, 2012.
28. Vansteelandt S, Goetghebeur E. Causal inference with generalized structural mean models. *Journal of the Royal Statistical Society: Series B (Statistical Methodology)* 2003;65(4):817-35. doi: 10.1046/j.1369-7412.2003.00417.x
29. Skarstrand E, Sundell K, Andreasson S. Evaluation of a Swedish version of the Strengthening Families Programme. *The European Journal of Public Health* 2014;24(4):578-84. doi: 10.1093/eurpub/ckt146
30. Segrott J, Holliday J, Rothwell H, et al. Cultural adaptation and intervention integrity: a response to Skarstrand, Sundell and Andreasson. *The European Journal of Public Health* 2014;24(3):354-55. doi: 10.1093/eurpub/cku039
31. Foxcroft DR, Callen H, Davies EL, et al. Effectiveness of the strengthening families programme 10–14 in Poland: cluster randomized controlled trial. *The European Journal of Public Health* 2016:ckw195. doi: 10.1093/eurpub/ckw195
32. Baldus C, Thomsen M, Sack PM, et al. Evaluation of a German version of the Strengthening Families Programme 10-14: a randomised controlled trial. *European journal of public health* 2016;26(6):953-59. doi: 10.1093/eurpub/ckw082 [published Online First: 2016/07/05]

For peer review only - http://bmjopen.bmj.com/site/about/guidelines.xhtml

33. Bonell C, Allen E, Warren E, et al. Effects of the Learning Together intervention on bullying and aggression in English secondary schools (INCLUSIVE): a cluster randomised controlled trial. *The Lancet* 2018;392(10163):2452-64. doi: 10.1016/s0140-6736(18)31782-3
34. Gorman DM. Flexible data analysis and evaluations of the SFP 10-14. *The European Journal of Public Health* 2015;25(5):900.2-01. doi: 10.1093/eurpub/ckv155
35. Wiggins M, Bonell C, Sawtell M, et al. Health outcomes of youth development programme in England: prospective matched comparison study. *BMJ* 2009;339(jul07 2):b2534-b34. doi: 10.1136/bmj.b2534
36. Spoth R, Trudeau L, Redmond C, et al. Replicating and extending a model of effects of universal preventive intervention during early adolescence on young adult substance misuse. *Journal of Consulting and Clinical Psychology* 2016;84(10):913-21. doi: 10.1037/ccp0000131
37. Spoth R, Trudeau L, Redmond C, et al. Replication RCT of Early Universal Prevention Effects on Young Adult Substance Misuse. *Journal of consulting and clinical psychology* 2014;82 doi: 10.1037/a0036840

For peer review only

Figure 1: Trial consort participant flow chart

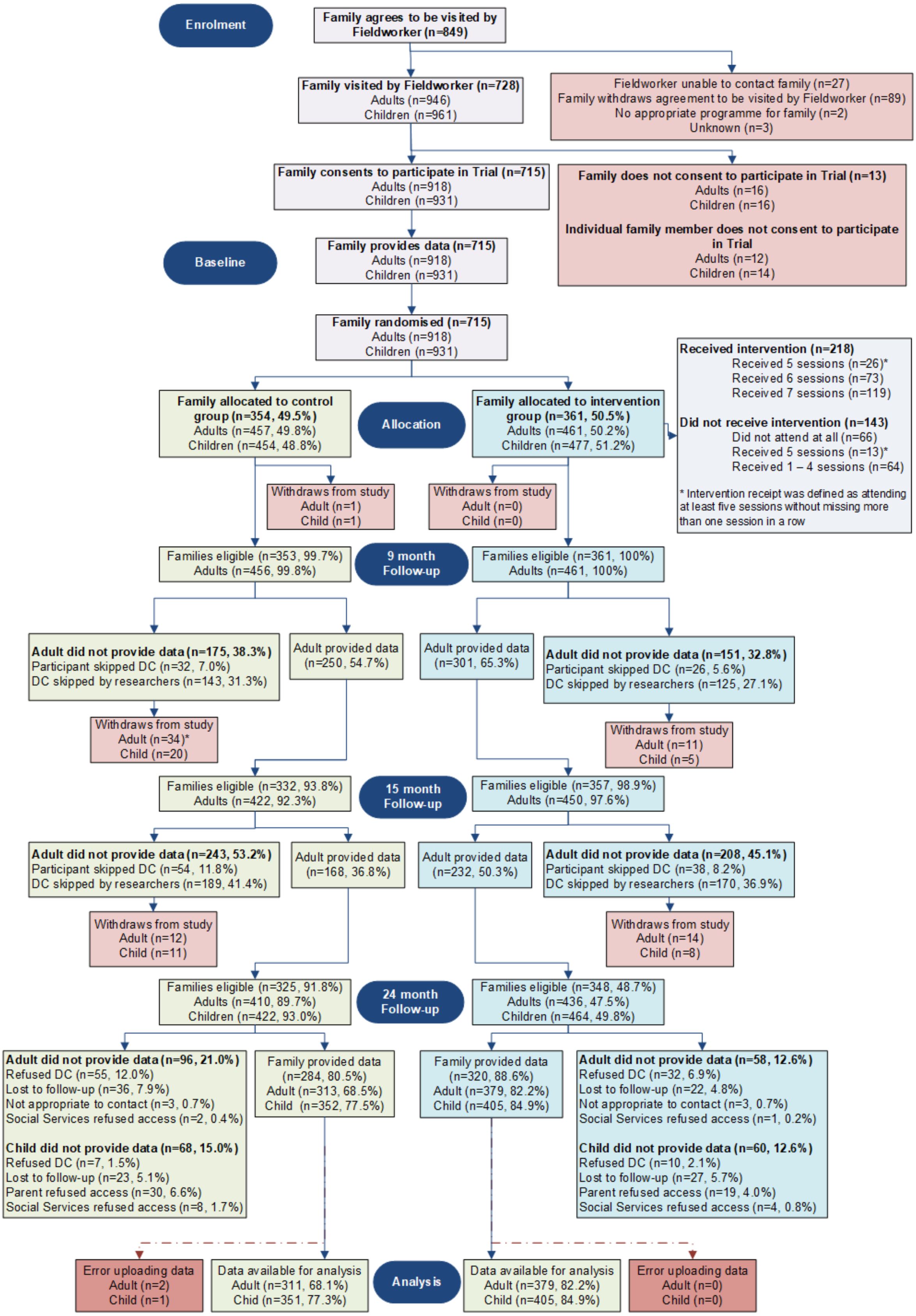

* 32 adults in the Control group withdrew at the 9 month data sweep. A further two adults withdrew prior to being scheduled for their 15 month data sweep

Supplementary material

Table S1: Measures collected at 24 month follow-up from young people

Domain/question topic	Purpose	Measure/Source
**Behaviour - Strengths and Difficulties**		
Strengths and difficulties / wellbeing and stress	TO	SDQ[1]
**Life at Home**		
Family activities (opportunities for involvement in pro-social activities; possibly bonding)	ST	From HBSC/ PEACH study
Young people's own time		From West of Scotland Twenty 07
Parenting/child management	IO/ TO	General Child Management Measure, Project Family[2-4]
Parents and school		Three questions; one adapted from the Child Rearing Practices Measure
Help around the home	IO	Adapted versions of questions asked to parents at 9/15 months.
Development of self-efficacy	ST/ TO	Bandura's Self Efficacy Scale[5]
Attachment to parents	ST	The Security Scale[6]
Befriending pro-social/anti-social peers	ST	Social Development Model scale on friends' anti-social qualities (Interaction with antisocial peers scale)[7]
Positive bonding to school	ST	School Bonding Measure / SDM[8]
**Participant substance use**		
Smoking behaviour – ever smoked?		Question from Substance Initiation Index[9]
Smoking status	TO/ SO	ASSIST study version of NatCen/NFER question[10]
Age first smoked	TO	From HBSC questionnaire
Ever drunk a drink?		NatCen/NFER[11]
Age of first drink	SO	Adapted from HBSC/NatCen/NFER
Drinking frequency, last month (including different types)	SO	Adapted questions from HBSC questionnaire
Ever really drunk?		From HBSC questionnaire
Age first drunk		Adapted version of question in HBSC questionnaire
Number of times drunk, drinking alcohol or 5+ drinks in a row in last month	PO/SO	Amended from HBSC questionnaire
Alcohol-related problems	SO	Q21 from ESPAD survey[12]
Drug use – ever used?		Amended from HBSC/NatCen/NFER[11]
Cannabis use (ever, 12 months, or 30 days)	SO	From HBSC questionnaire
Age of first drug use	TO	Adapted from HBSC questionnaire
**General health**		
Health state today	HE/TO	EQ-5D (child version)[13]
General health	HE/TO	UK Kidscreen 10[14]

For peer review only

Table S2: Measures collected at 24 month follow-up from parents/carers

Domain/question topic	Purpose	Measure/Source
**Life at home**		
Family activities		From HBSC/11-16 West of Scotland adult questionnaire
Family functioning	TO	Family relationship index[15,16]
Parenting/child management	SO, IO	General child management measure, Project Family[2-4]
Parent–child bonding	ST	Adapted from Spoth's rural urban cumulative risk index and Arthur, et al.[17,18]
Parents and school		Question developed by project team and one from Conger's child rearing practices measure
**Participant substance use**		
Smoking behaviour		Heaviness of smoking index[19]
Alcohol use		Adapted AUDIT-C questions, as used by Pre-empt study[20]
Drug use		Adapted from HBSC/NatCen/ NFER[11]
**Health**		
Health status	HE, TO	GHQ[21,22]
Health	HE	1 item from SF-36[23]
Health state today	HE, TO	EQ-5D[13]
**Service utilisation**		
Health, social care, education and criminal justice service utilisation	HE	Modified from ALSPAC

Secondary analyses of the primary outcome

After adjusting alcohol use outcome for gender of young people, time from randomisation to 24 month, and intervention receipt, the results from multilevel modelling indicated that none of the adjustment had altered the results that drawn from the primary analysis (Table 8). Although the alcohol use outcome measure had been restructured into an ordinary outcome measure, the results from ordinal regression model provided a corresponded proportional odds ratio of 1.09 (0.70-1.71) with adjustments and proportional odds ratio of 1.11 (0.73-1.67) without adjustments, which suggests that there was no evidence of a between-group difference.

For peer review only

Table S3: Secondary analyses of the primary alcohol use outcome

Analysis	Odds ratio	95% Confidence Interval		p-value
		Lower limit	Upper limit	
Without any adjustments	1.11	0.74	1.66	0.626
Adjusting for gender of young person	1.11	0.72	1.72	0.641
Adjusting for time from randomisation to 24-month follow-up	1.08	0.70	1.68	0.725
Adjusting for intervention receipt (binary definition)	1.21	0.79	1.86	0.384
Adjusting for intervention receipt (number of weeks)	1.03	0.97	1.09	0.382
Analysis	Proportional odds ratio	95% Confidence Interval		p-value
		Lower limit	Upper limit	
As an ordinal outcome (adjusted)	1.09	0.70	1.71	0.689
As an ordinal outcome (without any adjustments)	1.11	0.73	1.67	0.637

In terms of drunkenness in the previous 30 days, results show that the model without controlling any adjustments reduced the treatment effect odds ratio from 1.46 to 1.27 but did not alter the conclusions that could be drawn from the analysis (Table 9). Similarly, model adjusted for gender for young person, time from randomisation to 24 month, and intervention receipt did not alter the conclusions, which suggests that there was no evidence of a between-group difference.

Table S4: Secondary analyses of the primary drunkenness outcome

Analysis	Odds ratio	95% Confidence Interval		p-value
		Lower limit	Upper limit	
Without any adjustments	1.27	0.73	2.21	0.392
Adjusting for gender of young person	1.48	0.84	2.58	0.173
Adjusting for time from randomisation to 24-month follow-up	1.35	0.77	2.37	0.298
Adjusting for intervention receipt (binary definition)	1.50	0.80	2.79	0.203
Adjusting for intervention receipt (number of weeks)	1.06	0.97	1.16	0.196
Analysis	Proportional odds ratio	95% Confidence Interval		p-value
		Lower limit	Upper limit	
As an ordinal outcome (adjusted)	1.42	0.82	2.46	0.211
As an ordinal outcome (without any adjustments)	1.26	0.71	2.25	0.429

Primary outcomes – subgroup analysis

An association was found between age of young person at baseline and reported alcohol use at 24-month follow-up (1.82 [1.23-2.71]), but there was no evidence of any differential effect by trial arms (1.30 [0.85-1.98]). In terms of gender of young person, the odds ratios for main effects and subgroup interaction terms suggested that the intervention worked differently for males and females. However, both confidence intervals included one, which suggests that there was no evidence of a differential treatment effect by gender of young person. Additionally, the main effect of young person who reported smoked at baseline (2.08 [1.06-4.11]) and having at least one parent/carer categorised at high-risk from problematic drinking (1.93 [1.01-3.69]) were significantly associated with the alcohol use outcome, but there were no discernible differences between arms. Other planned subgroup analyses provided no evidence of the SFP group having a differential effect.

An association was found between young person who reported smoked at baseline and reported drunkenness at 24-month follow-up (5.73 [2.07-15.90]), but there was no evidence of any differential effect by trial arms (0.57 [0.17-1.94]). In terms of the average young person GCM score (General Child Management), a one-point increase on the GCM score was associated with a 59% decrease in the odds of being classed as a young person who had been drunk in the last 30 days (0.41 [0.18-0.96]) a (higher GCM score indicates greater levels of parent management). However, there was no evidence of any differential effect by trial arm (1.27 [0.45-3.56]). For

For peer review only

gender of young person, the odds ratio for main effect showed a 17% increase for females who reported having been drunk in the previous 30 days at 24-month follow up compared to males, and the subgroup interaction indicated an 8% increase for females allocated to the SFP group compared to males and those allocated to the control group. However, both confidence intervals included one, which suggests that there was no evidence of a differential treatment effect by gender of young person. Overall, none of the subgroup analyses for the primary drunkenness outcome proved a significant association, which suggests that there was no evidence of a differential treatment effect.

For peer review only

Table S5: Subgroup analyses for the primary alcohol use outcome

Subgroup	Main effect odds ratio	95% Confidence Interval		p-value	Subgroup x SFP interaction	95% Confidence Interval		p-value
		Lower limit	Upper limit			Lower limit	Upper limit	
Age of young person at baseline	1.82	1.23	2.71	0.003	1.30	0.85	1.98	0.228
Gender of young person (female)	0.85	0.44	1.57	0.574	1.48	0.65	3.57	0.337
Young person had smoked at baseline	2.08	1.06	4.11	0.034	0.79	0.32	1.91	0.596
Having at least one parent/carer who is at high risk from problematic drinking	1.93	1.01	3.69	0.048	0.79	0.33	1.87	0.593
Baseline Family Affluence Scale (Medium)	1.13	0.44	2.86	0.750	1.08	0.31	3.73	0.589
Baseline Family Affluence Scale (High)	1.09	0.39	3.00		1.75	0.46	6.70	
Occupation Status (At least one parent in the family in part-time (but none in full-time) employment)	0.60	0.24	1.53	0.448	0.86	0.24	3.09	0.720
At least one parent in the family in full-time employment	0.91	0.41	2.02		1.46	0.50	4.25	
Young person belonging to a "family with challenges"	0.99	0.52	1.91	0.984	0.51	0.21	1.24	0.140
Strengths and difficulties score at baseline	1.02	0.97	1.07	0.498	1.00	0.93	1.07	0.929
Cohesion domain of the Family Relationship Index at baseline	1.01	0.99	1.03	0.328	0.99	0.96	1.01	0.273
Expressiveness domain of the Family Relationship Index at baseline	1.01	0.99	1.04	0.339	0.99	0.96	1.03	0.760
Conflict domain of the Family Relationship Index at baseline	1.02	0.99	1.05	0.211	1.00	0.97	1.04	0.871
Average Young person General Child Management score	1.01	0.54	1.91	0.968	0.45	0.18	1.12	0.086
Average Parent/carer General Child Management score	1.54	0.76	3.15	0.233	0.67	0.28	1.64	0.383

For peer review only

Table S6: Subgroup analyses for the primary drunkenness outcome

Subgroup	Main effect odds ratio	95% Confidence Interval		p-value	Subgroup x SFP interaction	95% Confidence Interval		p-value
		Lower limit	Upper limit			Lower limit	Upper limit	
Age of young person at baseline	2.22	0.98	5.04	0.056	1.30	0.67	2.53	0.435
Gender of young person (female)	1.17	0.49	2.80	0.690	1.08	0.35	3.29	0.920
Young person had smoked at baseline	5.73	2.07	15.90	0.001	0.57	0.17	1.94	0.369
Having at least one parent/carer who is at high risk from problematic drinking	1.40	0.58	3.38	0.448	1.68	0.53	5.26	0.376
Baseline Family Affluence Scale (Medium)	1.26	0.36	4.43	0.336	2.57	0.46	14.45	0.563
Baseline Family Affluence Scale (High)	0.99	0.25	3.88		2.18	0.34	14.15	
Occupation Status (At least one parent in the family in part-time (but none in full-time) employment)	1.31	0.46	3.73	0.673	0.76	0.17	3.33	0.550
At least one parent in the family in full-time employment	0.76	0.25	2.30		1.87	0.47	7.43	
Young person belonging to a "family with challenges"	1.19	0.50	2.85	0.692	0.41	0.13	1.30	0.131
Strengths and difficulties score at baseline	0.97	0.90	1.04	0.366	1.03	0.94	1.13	0.570
Cohesion domain of the Family Relationship Index at baseline	1.01	0.98	1.03	0.578	0.99	0.96	1.03	0.730
Expressiveness domain of the Family Relationship Index at baseline	0.99	0.96	1.02	0.516	1.01	0.97	1.05	0.661
Conflict domain of the Family Relationship Index at baseline	1.00	0.97	1.04	0.791	0.99	0.94	1.03	0.584
Average Young person General Child Management score	0.41	0.18	0.96	0.040	1.27	0.45	3.56	0.649
Average Parent/carer General Child Management score	1.13	0.43	2.98	0.804	0.77	0.24	2.46	0.656

Weekly smoking validated by salivary cotinine measures

Overall, 500 young people (53.7% of those randomised, 66.1% of those who provided 24-month follow-up interview data) provided saliva cotinine samples and self-reported weekly smoking behaviour at the 24-month follow-up interview. To identify weekly smokers using saliva cotinine, a cut-off of 15ng/mL was used[10,24]. Comparing cotinine samples with self-reported weekly smoking, 467 young people provided self-reported smoking data that agreed with their cotinine samples (93.4%). A slightly higher percentage of young people in

For peer review only

the SFP group reported being a non-weekly smoker and had cotinine samples ≤ 15 ng/mL and less than those who reported that they were a weekly smoker but had cotinine levels of ≤ 15 ng/mL.

Table S7: Agreement between self-reported weekly smoking behaviour and saliva cotinine

Self-report and saliva cotinine agreement		Control		SFP		Total	
		n	%	n	%	n	%
Concordant	Agree non-weekly smoker	176	78.6	227	82.2	403	80.6
	Agree weekly smoker	30	13.4	34	12.3	64	12.8
Discordant	Self-reported non-weekly smoker and cotinine > 15 ng/mL	13	5.8	9	3.3	22	4.4
	Self-reported weekly smoker and cotinine ≤ 15 ng/mL	5	2.2	6	2.2	11	2.2
Total		224	100.0	276	100.0	500	100.0

For peer review only

References

1. Goodman R. The Strengths and Difficulties Questionnaire: A Research Note. *Journal of Child Psychology and Psychiatry* 1997; **38**(5): 581-6.
2. McMahon R, Metzler C. Selecting parenting measures for assessing family based prevention interventions. In: Ashery R, Robertson E, Kumpfer K, Rockville M, eds. Drug abuse prevention through family interventions: National Institute on Drug Abuse; 1998: 294-323.
3. Spoth R, Redmond C, Haggerty K, Ward T. A Controlled Parenting Skills Outcome Study Examining Individual Difference and Attendance Effects. 1995; **57**(2): 449.
4. Spoth R, Redmond C, Shin C. Direct and indirect latent-variable parenting outcomes of two universal family-focused preventive interventions: extending a public health-oriented research base. *Journal of consulting and clinical psychology* 1998; **66**(2): 385-99.
5. Bandura A. A Guide for Constructing Self-Efficacy Scales. In: Pajares F, Urdan T, Greenwich, CT, eds. Self-Efficacy Beliefs of Adolescents: Information Age Publishing; 2006: 307-37.
6. Kerns K, Aspelmeier J, Gentzler A, Grabill C. Parent–child attachment and monitoring in middle childhood. *Journal of Family Psychology* 2001; **15**: 69-81.
7. Arthur M, Hawkins J, Catalano R, Pollard J. Student Survey of Risk and Protective Factors and Prevalence of Alcohol, Tobacco, & Other Drug Use. University of Washington: Social Development Research Group.
8. Hawkins JD, Guo J, Hill KG, Battin-Pearson S, Abbott RD. Long-Term Effects of the Seattle Social Development Intervention on School Bonding Trajectories. *Applied Developmental Science* 2001; **5**(4): 225-36.
9. Spoth RL, Redmond C, Trudeau L, Shin C. Longitudinal substance initiation outcomes for a universal preventive intervention combining family and school programs. *Psychology of Addictive Behaviors* 2002; **16**(2): 129-34.
10. Campbell R, Starkey F, Holliday J, et al. An informal school-based peer-led intervention for smoking prevention in adolescence (ASSIST): a cluster randomised trial. *The Lancet* 2008; **371**(9624): 1595-602.
11. National Centre for Social Research NFfE, Research. Smoking, Drinking and Drug Use Among Young People in England

in 2008 Full Report: NHS Information Centre for Health and Social Care, 2009.
12. Hibell B, Guttormsson U, Ahlström S, et al. The 2011 ESPAD Report: Substance Use Among Students in 36 European Countries: The Swedish Council for Information on Alcohol and other Drugs (CAN), 2011.
13. Rabin R, Charro FD. EQ-SD: a measure of health status from the EuroQol Group. *Annals of Medicine* 2001; **33**(5): 337-43.
14. The Kidscreen Group Europe. The KIDSCREEN Questionnaires: Quality of life questionnaires for children and adolescents: Handbook. Lengerich: Pabst Science Publishers, 2006.
15. Holahan CJ, Moos RH. Social support and adjustment: Predictive benefits of social climate indices. 1982; **10**(4): 403-15.
16. Billings AG, Moos RH. The role of coping responses and social resources in attenuating the stress of life events. *Journal of Behavioral Medicine* 1981; **4**(2): 139-57.

For peer review only

17. Spoth R, Goldberg C, Neppl T, Trudeau L, Ramisetty-Mikler S. Rural–urban differences in the distribution of parent-reported risk factors for substance use among young adolescents. *Journal of Substance Abuse* 2001; **13**(4): 609-23.
18. Arthur M, Hawkins J, Catalano R, Pollard J. Item-construct dictionary for the student survey of risk and protective factors and prevalence of alcohol, tobacco, and other drug use. Unpublished Technical Document. . Seattle, WA.: University of Washington, 1995.
19. Borland R, Yong HH, O'Connor RJ, Hyland A, Thompson ME. The reliability and predictive validity of the Heaviness of Smoking Index and its two components: Findings from the International Tobacco Control Four Country study. 2010; **12**(Supplement 1): S45-S50.
20. Bush K. The AUDIT Alcohol Consumption Questions (AUDIT-C)<subtitle>An Effective Brief Screening Test for Problem Drinking</subtitle>. *Archives of Internal Medicine* 1998; **158**(16): 1789.
21. Goldberg D. The Detection of Psychiatric Illness by Questionnaire. London: Oxford University Press; 1972.
22. Vieweg BW, Hedlund JL. The General Health Questionnaire (GHQ): A comprehensive review. *Journal of Operational Psychiatry* 1983; **14**(2): 74-81.
23. Ware JE, Sherbourne CD. The MOS 36-ltem Short-Form Health Survey (SF-36). *Medical Care* 1992; **30**(6): 473-83.
24. Benowitz NL, Iii PJ, Ahijevych K, et al. Biochemical verification of tobacco use and cessation. *Nicotine & Tobacco Research* 2002; **4**(2): 149-59.

For peer review only

Checklist of items for reporting pragmatic trials

Section	Item	Standard CONSORT description	Extension for pragmatic trials	Location in the submitted paper
Title and abstract	1	How participants were allocated to interventions (eg, "random allocation," "randomised," or "randomly assigned")		p1 (title); p2 (abstract)
**Introduction**				
Background	2	Scientific background and explanation of rationale	Describe the health or health service problem that the intervention is intended to address and other interventions that may commonly be aimed at this problem	p4
**Methods**				
Participants	3	Eligibility criteria for participants; settings and locations where the data were collected	Eligibility criteria should be explicitly framed to show the degree to which they include	pp5-6

For peer review only

Section	Item	Standard CONSORT description	Extension for pragmatic trials	Location in the submitted paper
			typical participants and/or, where applicable, typical providers (eg, nurses), institutions (eg, hospitals), communities (or localities eg, towns) and settings of care (eg, different healthcare financing systems)	
Interventions	4	Precise details of the interventions intended for each group and how and when they were actually administered	Describe extra resources added to (or resources removed from) usual settings in order to implement intervention. Indicate if efforts were made to standardise the intervention or if the intervention and its delivery were allowed to vary between	P5, p13

For peer review only

Section	Item	Standard CONSORT description	Extension for pragmatic trials	Location in the submitted paper
			participants, practitioners, or study sites	
			Describe the comparator in similar detail to the intervention	p6
Objectives	5	Specific objectives and hypotheses		p5
Outcomes	6	Clearly defined primary and secondary outcome measures and, when applicable, any methods used to enhance the quality of measurements (eg, multiple observations, training of assessors)	Explain why the chosen outcomes and, when relevant, the length of follow-up are considered important to those who will use the results of the trial	Pp7-8

For peer review only

Section	Item	Standard CONSORT description	Extension for pragmatic trials	Location in the submitted paper
Sample size	7	How sample size was determined; explanation of any interim analyses and stopping rules when applicable	If calculated using the smallest difference considered important by the target decision maker audience (the minimally important difference) then report where this difference was obtained	p8
Randomisation—sequence generation	8	Method used to generate the random allocation sequence, including details of any restriction (eg, blocking, stratification)		p7
Randomisation—allocation concealment	9	Method used to implement the random allocation sequence (eg, numbered containers or central telephone), clarifying whether the sequence was concealed until interventions were assigned		p7

For peer review only

Section	Item	Standard CONSORT description	Extension for pragmatic trials	Location in the submitted paper
Randomisation—implementation	10	Who generated the allocation sequence, who enrolled participants, and who assigned participants to their groups		p7
Blinding (masking)	11	Whether participants, those administering the interventions, and those assessing the outcomes were blinded to group assignment	If blinding was not done, or was not possible, explain why	p7
Statistical methods	12	Statistical methods used to compare groups for primary outcomes; methods for additional analyses, such as subgroup analyses and adjusted analyses		pp8-9
**Results**				
Participant flow	13	Flow of participants through each stage (a diagram is strongly recommended)—specifically, for each group, report the numbers of participants randomly assigned,	The number of participants or units approached to take part in the trial, the number which were	Summarised on p9, with full details

For peer review only

Section	Item	Standard CONSORT description	Extension for pragmatic trials	Location in the submitted paper
		receiving intended treatment, completing the study protocol, and analysed for the primary outcome; describe deviations from planned study protocol, together with reasons	eligible, and reasons for non-participation should be reported	included in Figure 1
Recruitment	14	Dates defining the periods of recruitment and follow-up		p9
Baseline data	15	Baseline demographic and clinical characteristics of each group		p10
Numbers analysed	16	Number of participants (denominator) in each group included in each analysis and whether analysis was by "intention-to-treat"; state the results in absolute numbers when feasible (eg, 10/20, not 50%)		Number of participants included in analysis

For peer review only

Section	Item	Standard CONSORT description	Extension for pragmatic trials	Location in the submitted paper
				described on p42. Use of ITT described on p8.
Outcomes and estimation	17	For each primary and secondary outcome, a summary of results for each group and the estimated effect size and its precision (eg, 95% CI)		Pp10-13
Ancillary analyses	18	Address multiplicity by reporting any other analyses performed, including subgroup analyses and adjusted analyses, indicating which are prespecified and which are exploratory		Pp11-14

For peer review only

Section	Item	Standard CONSORT description	Extension for pragmatic trials	Location in the submitted paper
Adverse events	19	All important adverse events or side effects in each intervention group		p14
**Discussion**				
Interpretation	20	Interpretation of the results, taking into account study hypotheses, sources of potential bias or imprecision, and the dangers associated with multiplicity of analyses and outcomes		Pp14-16
Generalisability	21	Generalisability (external validity) of the trial findings	Describe key aspects of the setting which determined the trial results. Discuss possible differences in other settings where clinical traditions, health service organisation, staffing, or resources may vary from those of the trial	Pp14-16

For peer review only

Section	Item	Standard CONSORT description	Extension for pragmatic trials	Location in the submitted paper
Overall evidence	22	General interpretation of the results in the context of current evidence		Pp14-16

Cite as: *Zwarenstein M, Treweek S, Gagnier JJ, Altman DG, Tunis S, Haynes B, Oxman AD, Moher D for the CONSORT and Pragmatic Trials in Healthcare (Practihc) group. Improving the reporting of pragmatic trials: an extension of the CONSORT statement. BMJ 2008; 337;a2390.*

For peer review only

The effectiveness of the Strengthening Families Programme in the UK at preventing substance misuse in 10-14 year olds: a pragmatic randomised controlled trial

Journal:	*BMJ Open*
Manuscript ID	bmjopen-2021-049647.R2
Article Type:	Original research
Date Submitted by the Author:	23-Nov-2021
Complete List of Authors:	Segrott, Jeremy; Cardiff University, Centre for Trials Research; Cardiff University, DECIPHer Centre, Cardiff School of Social Sciences<br>Gillespie, David; Cardiff University, Centre for Trials Research<br>Lau, Mandy; Cardiff University, Centre for Trials Research<br>Holliday, Jo; University of Oxford, UK Biobank, Big Data Institute<br>Murphy, Simon; Cardiff University, DECIPHer Centre, Cardiff School of Social Sciences Cardiff, UK<br>Foxcroft, David; Oxford Brookes University UK, Department of Psychology, Health and Professional Development, Faculty of Health and Life Sciences<br>Hood, Kerenza; Cardiff University, Centre for Trials Research<br>Scourfield, Jonathan; Cardiff University, School of Social Sciences<br>Phillips, Ceri; Swansea University, Human and Health Sciences<br>Roberts, Zoe; Cardiff University, Centre for Medical Education, School of Medicine<br>Rothwell, Heather; Cardiff University, DECIPHer Centre, Cardiff School of Social Sciences<br>Hurlow, Claire; Swansea University, Swansea Trials Unit, Swansea University Medical School<br>Moore, L; University of Glasgow, MRC/CSO Social and Public Health Sciences Unit
**Primary Subject Heading**:	Public health
Secondary Subject Heading:	Public health
Keywords:	PUBLIC HEALTH, Substance misuse < PSYCHIATRY, Community child health < PAEDIATRICS

SCHOLARONE™
Manuscripts

I, the Submitting Author has the right to grant and does grant on behalf of all authors of the Work (as defined in the below author licence), an exclusive licence and/or a non-exclusive licence for contributions from authors who are: i) UK Crown employees; ii) where BMJ has agreed a CC-BY licence shall apply, and/or iii) in accordance with the terms applicable for US Federal Government officers or employees acting as part of their official duties; on a worldwide, perpetual, irrevocable, royalty-free basis to BMJ Publishing Group Ltd ("BMJ") its licensees and where the relevant Journal is co-owned by BMJ to the co-owners of the Journal, to publish the Work in this journal and any other BMJ products and to exploit all rights, as set out in our licence.

The Submitting Author accepts and understands that any supply made under these terms is made by BMJ to the Submitting Author unless you are acting as an employee on behalf of your employer or a postgraduate student of an affiliated institution which is paying any applicable article publishing charge ("APC") for Open Access articles. Where the Submitting Author wishes to make the Work available on an Open Access basis (and intends to pay the relevant APC), the terms of reuse of such Open Access shall be governed by a Creative Commons licence – details of these licences and which Creative Commons licence will apply to this Work are set out in our licence referred to above.

Other than as permitted in any relevant BMJ Author's Self Archiving Policies, I confirm this Work has not been accepted for publication elsewhere, is not being considered for publication elsewhere and does not duplicate material already published. I confirm all authors consent to publication of this Work and authorise the granting of this licence.

For peer review only

The effectiveness of the Strengthening Families Programme in the UK at preventing substance misuse in 10-14 year olds: a pragmatic randomised controlled trial

Jeremy Segrott (0000-0001-6215-0870), David Gillespie, Mandy Lau, Jo Holliday, Simon Murphy, David Foxcroft, Kerenza Hood, Jonathan Scourfield, Ceri Phillips, Zoe Roberts, Heather Rothwell, Claire Hurlow, Laurence Moore

[1, 2]Jeremy Segrott, Senior Lecturer, segrottj@cardiff.ac.uk
[1]David Gillespie, Senior Research Fellow, gillespied1@cardiff.ac.uk
[1]Mandy Lau, Research Associate, lautm@cardiff.ac.uk
[3]Jo Holliday, Health Data Group Lead, jo.holliday@ndph.ox.ac.uk
[1]Simon Murphy, Professor in Social Interventions and Health, Director DECIPHer and Lead for the Schools Health Research Network, murphys7@cardiff.ac.uk
[4]David Foxcroft, Professor of Community Psychology and Public Health, david.foxcroft@brookes.ac.uk
[1]Kerenza Hood, Director of the Centre for Trials Research, hoodk1@cardiff.ac.uk
[5]Jonathan Scourfield, Professor of Social Work, scourfield@cardiff.ac.uk
[6]Ceri Phillips, Professor Emeritus, c.j.phillips@swansea.ac.uk
[7]Zoe Roberts, Lecturer robertsze@cardiff.ac.uk
[2]Heather Rothwell, Retired
[8]Claire Hurlow, Study Manager, c.e.hurlow@swansea.ac.uk
[9]Laurence Moore, Director, MRC/CSO Social & Public Health Sciences Unit, Laurence.Moore@glasgow.ac.uk

[1]Centre for Trials Research, Cardiff University, Heath Park, Cardiff, UK. CF14 4YS
[2]DECIPHer Centre, Cardiff University, 1-3 Museum Place, Cardiff, UK. CF10 3BD.
[3]UK Biobank, Big Data Institute, University of Oxford, Li Ka Shing Centre for Health Information and Discovery, Old Road Campus, Roosevelt Drive, Oxford, UK. OX3 7BN
[4]Department of Psychology, Health and Professional Development, Faculty of Health and Life Sciences, Oxford Brookes University, Headington Campus, Oxford, UK. OX3 0BP.
[5]School of Social Sciences, Cardiff University, Glamorgan Building, King Edward VII Avenue, Cardiff, UK. CF10 3WT.
[6]College of Human and Health Sciences, Swansea University, Singleton Park, Swansea, UK. SA2 8PP.
[7]Centre for Medical Education, School of Medicine, Cardiff University, Heath Park, Cardiff, UK. CF14 4XN
[8]Swansea Trials Unit, Swansea University Medical School, Swansea University, Singleton Park, Swansea, UK. SA2 8PP.
[9]MRC/CSO Social & Public Health Sciences Unit, University of Glasgow, Berkeley Square, 99 Berkeley Street, Glasgow, UK. G3 7HR.

Correspondence to: Dr J Segrott, Centre for Trials Research, Cardiff University, 4th Floor, Neuadd Meirionnydd, Heath Park, Cardiff, CF14 4YS. Email: segrottj@cardiff.ac.uk

Word count for main text: 6048

For peer review only - http://bmjopen.bmj.com/site/about/guidelines.xhtml

Abstract

Objectives
The Strengthening Families Programme 10–14 (SFP10-14) is a USA-developed universal group-based intervention aiming to prevent substance misuse by strengthening protective factors within the family. This study evaluated a Proportionate Universal implementation of the adapted UK version (SFP10-14UK) which brought together families identified as likely/not likely to experience/present challenges within a group setting.

Design
Pragmatic cluster-randomised controlled effectiveness trial, with families as the unit of randomisation and embedded process and economic evaluations.

Setting
The study took place in seven counties of Wales, UK.

Participants
715 families (919 parents/carers, 931 young people) were randomised.

Interventions
Families randomised to the intervention arm received the Strengthening Families Programme 10–14 (SFP10-14) comprising 7 weekly sessions. Families in intervention and control arms received existing services as normal.

Outcome measures
Primary outcomes were the number of occasions young people reported drinking alcohol in the last 30 days; and drunkenness during the same period, dichotomised as 'never' and '1-2 times or more'. Secondary outcomes examined alcohol/tobacco/substance behaviours including: cannabis use; weekly smoking (validated by salivary cotinine measures); age of alcohol initiation; frequency of drinking >5 drinks in a row; frequency of different types of alcoholic drinks; alcohol-related problems. Retention: Primary analysis included 746 young people (80.1%) (alcohol consumption) and 732 young people (78.6%) (drunkenness).

Results
There was no evidence of statistically significant between-group differences 2 years post-randomisation for primary outcomes (young people's alcohol consumption in the last 30 days adjusted odds ratio=1.11, 95% CI: 0.72 to 1.71, p=0.646; drunkenness in the last 30 days adjusted odds ratio=1.46, 95% CI: 0.83 to 2.55, p=0.185). There were no statistically significant between-group differences for other substance use outcomes, or those relating to wellbeing/stress, and emotional/behavioural problems.

Conclusions
Previous evidence of effectiveness was not replicated. Findings highlight the importance of evaluating interventions when they are adapted for new settings.

Trial registration
Current Controlled Trials ISRCTN63550893.

Keywords
Substance misuse, prevention, RCT, Strengthening Families Programme 10-14(UK), SFP10-14UK, young people, parents, family.

For peer review only

Strengths and Limitations

- Both trial arms had good participant retention rates at two-year follow-up.
- Recruitment, intervention delivery systems, and implementation replicated how the intervention would be delivered outside a trial setting, thus maximising applicability of findings.
- There were good levels of agreement between self-reported smoking behaviour and cotinine levels in collected saliva samples.
- Most outcomes – including the primary outcomes relating to alcohol, could only be measured using self-report.

For peer review only

For peer review only - http://bmjopen.bmj.com/site/about/guidelines.xhtml

Title: The effectiveness of the Strengthening Families Programme in the UK at preventing substance misuse in 10-14 year olds: a pragmatic RCT

Introduction

Prevention of substance misuse among young people is a key public health priority internationally. Early initiation of alcohol consumption and drug use during adolescence is associated with short term negative impacts (e.g. accidental injury, educational attainment) and poorer health over the life course, including substance use disorders and mental health problems. Family-based risk and protective factors, encompassing parental modelling of alcohol use,[1] substance use rules and monitoring,[2] and parenting styles and family relationships, have been identified as important,[3] and influence a range of outcomes alongside substance use such as adolescent mental health.[4] This has led to considerable policy interest in developing prevention interventions targeting key parenting/family variables[5] and a need to develop the international evidence base.[6]

The Strengthening Families Programme 10–14 (SFP10-14) is a USA-developed universal group-based intervention that aims to prevent substance misuse in young people by strengthening protective factors within the family.[7]

Three main trials of SFP10-14 in the USA using a universal delivery model have been conducted – each led by the team responsible for intervention development at Iowa State University (ISU). These trials have demonstrated the programme's effectiveness in delaying the onset of alcohol use, reducing uptake of smoking, the incidence of harder drug use (methamphetamine)[8 9] (6.5 years past baseline) and substance use at 10 year follow-up.[10] However, findings from the trials conducted in the USA have been criticised in relation to data analysis and reporting practices, sample 'selection bias', and low rates of intervention attendance.[11 12] For example, Gorman's methodological critique of the ISU's Capable Families and Youth (CAFY) evaluation argues that:

> … there were no statistically significant results reported using 2-tailed tests for individual point-in-time measures of alcohol, cigarettes and marijuana use, or for the drunkenness measure, in the CaFaY evaluation. The five effects reported as statistically significant were based on 1-tailed tests of significance and one of these was for a measure unique to that publication and two were based on a highly irregular practice of using posttest data as the baseline[12] (p35).

A critique by the same author of the third ISU- led trial – PROSPER, notes that only 19% of young people allocated to the intervention attended at least one SFP10-14 session. Another critique of this study is that it fails to provide adequate details concerning rates of refusal by participants to provide data at 7.5 years follow-up, and uncertainty regarding how many of those in the intervention arm who did complete questionnaires had attended any SFP10-14 session. [11]

The current study evaluates the version of the intervention that was culturally adapted for the UK (SFP10-14UK). It seeks to address some of the key limitations of the ISU-led trials. Steps to achieve this include pre-specification of outcomes and analysis plans, prospective publication of a trial protocol, and comprehensive reporting of data on recruitment, retention and intervention attendance rates.

Universal interventions (provided without recourse to criteria relating to risk/need) may inadvertently perpetuate inequalities because of their sometimes low reach among those most at need.[13] Targeted interventions may also face difficulties in identifying and recruiting such groups, create barriers to engagement through stigmatisation of potential participants[14] and generate iatrogenic effects through reinforcement and normalisation of harmful norms.[15 16] Targeted interventions – which bring together families with high levels of need, may also present problems in relation to feasibility of delivering activities with fidelity.[17] A distinctive aspect of this trial is that the intervention was implemented using a Proportionate Universal (PU) approach – combining universal provision but with its intensity/scale varied according to need.[18] This aimed to address some of the limitations of both universal and targeted approaches described above and operationalise delivery of a universal intervention within an existing UK system mainly geared towards targeted interventions. Driven by a desire to address health and social inequalities, a Proportionate Universal approach provides services universally but "at a scale and intensity of action […] proportionate to the level of disadvantage", thus aiming to improve engagement with, and effectiveness among groups with higher needs [19] (p16). Universal provision is designed to achieve sustainability, secure political and financial support, and avoid stigmatisation [20]. Variation in the "scale and intensity" of interventions is applied to recruitment (greater effort/resources may be needed to engage certain social groups) and during programme implementation to meet differing needs, aiding participant retention. Thus, within a Proportionate Universal approach, targeting may comprise aspects of both *selectivism*

For peer review only - http://bmjopen.bmj.com/site/about/guidelines.xhtml

(identifying whether certain groups should be targeted and how) and *particularism* – how interventions can be tailored to meet differing needs[21].

The Proportionate Universalism approach adopted had two key components. First, at recruitment stage the intervention was provided on a universal basis but with greater efforts and resources dedicated to recruiting those families with higher level support needs. This recruitment strategy aimed to reach families with higher level needs whilst avoiding creating barriers to engagement, and the limitations of a purely targeted approach in identifying such families.

Second, groups of families brought together for the intervention were composed of approximately 30% who were likely to experience or present challenges within a group setting - e.g. young people not attending or excluded from school/class/year group or working with behavioural support teams; Autistic Spectrum Condition; aggression/anger; low literacy skills; learning difficulties; mental health issues; English/Welsh not being a family's first language; and substance misuse (parent or young person), and 70% without such challenges. In line with a Proportionate Universal approach, this group composition was designed to ensure that there were sufficient numbers of staff to provide additional support to families who might experience or present challenges within a group setting. It was also designed to enable delivery of activities as intended within a highly manualised and structured intervention, and to promote prosocial group dynamics, with the goal of facilitating behaviour change processes hypothesized to generate the intervention's intended impacts.

Aim

The trial's primary objective was to assess the effectiveness of the SFP 10-14 UK in preventing alcohol misuse in young people. Its secondary objectives were to assess the programme's impact on drug misuse, smoking behaviour, alcohol initiation and drink-related problems and school performance, among young people. Tertiary objectives were to measure the extent to which SFP 10-14 UK had effects on young people's mental health and wellbeing, and protective factors for alcohol and tobacco use/misuse (e.g. family functioning, parenting and young people's peer resistance skills). The trial encompassed a cost-consequences analysis to assess whether the intervention was cost effective. It assessed if there were important variations in delivery and receipt across trial sites, and identified key programme theory, content, and processes.

Methods

Study design

The trial comprised a pragmatic cluster randomised controlled effectiveness trial with families as the unit of randomisation, and all individuals within a family recruited into the study allocated to the same arm. There were embedded process and economic evaluations. The study took place in seven counties of Wales, UK. Ethical approval for the trial was given by the Research Ethics Committee for Wales (reference 09/MRE09/53). Full details of the design, methods and outcomes have been published in the study protocol.[22] Programme delivery systems – including staffing arrangements and participant eligibility were designed to mirror future 'real world' implementation outside of a research context to maximise generalisability of findings. The researchers did not impose any additional standardisation of intervention delivery. Local authorities and third sector organisations with responsibility for supporting families or substance misuse prevention led multiple agency partnerships that delivered the programme in community settings. The trial was prospectively registered with ISCTRN registry on 3rd December 2009, and prior to any participants being enrolled.

Intervention

Drawing on theoretical models relating to bio-psychosocial vulnerability, youth resiliency and family process, SFP10-14 addresses risk and protective factors within the family environment known to shape later substance use behaviours [23]. It comprises 7 weekly sessions each lasting two hours. In the first hour parallel groups of young people and parents/carers from up to 12 families develop their understanding and skills, led by trained facilitators. Each session is guided by a facilitator manual which details activities and how they are to be delivered. In the second hour parents/carers and young people come together in family units to practise key skills. The programme has been culturally adapted for the UK (SFP10-14UK). Adaptations comprised changes to language and terms to fit with a UK context, and production of revised videos which used 'real life' situations filmed in home settings instead of studio-based narration. Core content and function of the intervention were retained and not subjected to significant change. [24]A logic model for the culturally adapted intervention has been developed by the authors[25].

Participants

For peer review only - http://bmjopen.bmj.com/site/about/guidelines.xhtml

Each programme was open to families with young people aged 10–14 from a large geographical area (e.g. town or rural district). Self-referrals to the Programme came forward in response to awareness-raising in community and educational settings. This included distribution of fliers to parents across relevant school year groups, attendance at community events, promotion via social media and placing of leaflets in local venues such as leisure centres. Other referrals came from agencies such as education, health and social services which identified families that may benefit from participating in the Programme. Potential referrers and families were informed that the Programme was being run as part of a trial and were provided with information about the trial. When a family was referred or applied to the SFP 10–14UK they were visited by a member of the programme delivery team who undertook a needs and eligibility assessment, which included exploration of any difficulties or support needs which participants might experience within a group setting. For example, this encompassed exploring the support needs of those with low literacy levels (as much of the programme involved text-based materials) or addressing how conflict within families might manifest when they came to work together on activities during sessions. Based on the information contained in the family referral/application form and the needs and eligibility assessment, the coordinator determined if eligible families were likely to experience or present challenges within a group setting. If families were deemed eligible to attend the programme they were asked if they were willing for a member of the research team to have access to their referral notes and to visit them. Where the family agreed, a research fieldworker visited them to obtain informed consent to participate in the trial and collect baseline data using a computer assisted personal interview (CAPI).

Any family deemed eligible to attend SFP 10–14 UK was included in the research trial, subject to their giving consent. Trial inclusion and exclusion criteria are listed in Table 1. Informed consent was obtained from all participants. For a family to be included in the research trial at least one parent/carer needed to consent to participate, and parent/carer consent for the inclusion of at least one young person in the trial was required. Young people also gave consent for their participation in the trial.

For peer review only

Table 1: Trial inclusion and exclusion criteria

Inclusion criteria	Exclusion criteria
Families in which at least one parent/carer and one young person are willing to attend the programme together	Families in which either a parent or young person does not want to attend the programme
Families with the ability to speak English (help can be provided for parents or young people with low literacy levels). Some programmes may also be delivered through the medium of Welsh if there is sufficient demand	Families in which there are either parents or young people who cannot speak English (or Welsh, where appropriate)
Families where a programme is being offered at a location to which it is practicable for a family to travel (as determined by the programme coordinator) within the next three months	Families where there is no programme being offered at a location to which it is practicable for a family to travel (as determined by the programme coordinator) within the next three months. In such a case the family would not be excluded. They will be placed on a waiting list for the programme and will be contacted when a programme is available. They will then be recruited into the trial
Families with a young person aged 10-14	-
-	Families who do not live together - e.g. the child/children are in care
-	Families with very high needs or challenges (such as serious substance misuse problems, family breakdown or crisis)

Randomisation and masking Procedures

Remote computerised randomisation was conducted by the research fieldworker at the end of the baseline interview once consent and baseline data had been obtained. Families were randomised within strata defined by the seven areas in which the study was conducted and using minimisation with a random element set at 80%. The minimisation algorithm aimed to achieve balance between randomised groups on family categorisation (family without challenge/family with challenges in a group setting), the average age of the young people within a family who were recruited into the trial (<12/12+), and the number of young people within a family who were recruited into the trial (1/>1). Families randomised to the intervention arm received SFP10-14UK in addition to usually provided services. Those randomised to the control continued to receive usually provided services only. These services included local authority parenting and family services, school-based support, and other providers' services such as substance misuse prevention teams, child, and adolescent mental health (CAMHS) and programmes operated by charities. There was some variation in the provision and organisation of services across the trial areas. Data on families' health, social care, education, and criminal justice service utilisation was collected from parents/carers at 9-, 15- and 24-month follow-up points. Due to the nature of the intervention, it was not possible for participants or those delivering the intervention to be blinded to families' group assignment. Assessment of trial outcomes was done blind to group assignment.

Outcomes

The trial had two primary outcomes - the number of occasions that young people reported having drunk alcohol in the last 30 days; and drunkenness during the last 30 days, dichotomised as 'never' and '1-2 times or more'.

Secondary outcomes were: reported use of cannabis (ever vs. never); weekly smoking (yes vs no, validated by salivary cotinine measures); age of alcohol use initiation; frequency of drinking more than 5 drinks in a row in the last 30 days; frequency of different types of alcoholic drinks; alcohol-related problems; and General Certificate of Secondary Education (GCSE) performance at age 15/16 (number of GCSEs passed and grades achieved, measured as a continuous outcome). All primary and secondary outcomes were collected from children at 2-year follow-up, with the exception of GCSE results, which it was proposed to collect via the Secure Anonymised Information Linkage (SAIL) Databank[26], once all participants had completed GCSEs/left compulsory education.

The trial's tertiary outcomes were: parenting (General Child Management Scale child report); family functioning (Family Relationship Index); children's wellbeing and stress (Strengths and Difficulties Questionnaire scores [SDQ]); children's health status (SF-36); parents/carers' health status (General Health Questionnaire - GHQ and EQ5D); costs and an assessment of relative cost-effectiveness, derived from a cost-consequences analysis; children's smoking status (i.e. whether they have ever smoked/smoked now); young

For peer review only - http://bmjopen.bmj.com/site/about/guidelines.xhtml

people's self-efficacy; age of first cigarette; and age of first drug use. Tables S1-S2 (Supplementary material) provide a description of the trial's primary, secondary and tertiary outcomes, and the specific measures used at main follow-up at 24 months.

Selection of outcomes and length of follow-up were driven by the intervention's hypothesized mechanisms of action and impacts, including long term substance use prevention, and short/medium term mediators, particularly family relationships and parenting.

Procedures were put in place to assess and respond to reports of adverse events. Serious Adverse Events (SAEs) were reviewed by a senior member of the trial team within 24 hours to assess the nature of the SAE, its seriousness, causality, and expectedness. Where an SAE was both related (resulting from administration of any of the research procedures) and unexpected the Trial Manager notified the REC within 15 days of receiving notification of the SAE.

Cost-consequences analysis
The costs of the programme were identified, measured and valued in monetary terms and combined with changes in resource utilisation of services as a result of programme participation. Distinctions were made between the costs incurred in each programme area to assess variation and potential for efficiency gains. The costs of delivering the programmes were derived from conversations with relevant colleagues and comprised staff time (plus training costs), venue and equipment costs, provision of support facilities, promotional materials and other resources involved in patient recruitment, together with costs incurred by participants. The cost per participant and cost per family were computed for the study and for each of the areas of implementation. The extent to which participation resulted in changes in utilisation of services was assessed by undertaking a series of participant interviews. Changes to resource utilisation were translated into monetary terms using appropriate published unit cost data and merged with the costs of programme delivery to generate the net cost of the programme, net cost per family, net cost per adult and net cost per young person. The net cost of delivering the programme was used alongside differences in primary, secondary and tertiary outcomes to generate a set of ratios of relative cost-effectiveness within the study period. Sensitivity analyses were undertaken to assess the extent to which changes in parameter estimates affected the baseline findings.

Statistical analysis
We aimed to randomise 756 families in total (378 per arm). Our target sample size provides 80% power with a two-sided alpha at 0.025 (halved to account for two primary outcomes) to detect either a 12 percentage point difference in young people reporting having drunk alcohol at the 24-month follow-up point (assuming a control group prevalence of 48%)[27] or a 10 percentage point difference in young people reporting having been drunk at the 24-month follow-up point (assuming a control group prevalence of 22%)[27]. The sample size was inflated based on the average family including 1.25 young people in the trial, an intracluster correlation coefficient of 0.2, and 25% loss to follow-up.

Descriptive statistics were calculated using means (standard deviation), medians (interquartile range) and percentages as appropriate.

The primary analyses were conducted on a modified intention to treat population (participants who provided outcome data analysed in the arm to which they were randomised) and involved fitting two-level logistic regression models to whether or not young people reported drinking alcohol/being drunk in the 30 days prior to their 24-month interview, with responses from young people nested within families. The models controlled for the variables balanced on at randomisation (other than number of young people, as this was balanced on to ensure equal number of young people per groups, rather than scientific reasons), the study area, and the baseline-reported alcohol use/drunkenness (depending on the outcome). To test the strength of the findings of the primary analyses, the models were re-analysed controlling for gender, time from randomisation to 24-month follow-up, and without controlling for any covariates (pre-planned analyses). The primary outcomes were also analysed as ordinal variables (i.e. the number of occasions in the last 30 days that young people reported drinking alcohol/being drunk). The three categories used in the analysis were 'no occasions', '1-2 occasions' and '3 or more occasions'. Two-level ordinal regression models were fitted to both outcomes, with and without adjusting for variables balanced on at randomisation and baseline alcohol use/drunkenness.

In order to assess the impact that intervention non-receipt had on the findings of the primary analyses, double logistic structural mean models using generalised method of moments were fitted to each primary outcome.[28] A binary definition of intervention receipt was agreed by the study team a priori which involved participants

For peer review only - http://bmjopen.bmj.com/site/about/guidelines.xhtml

attending at least five sessions, without missing more than one session in a row. The quantitative measure of intervention receipt was also used (i.e. the number of weeks attended).

Multiple imputation was used in a sensitivity analysis to assess the impact of missing primary outcome data on the conclusions drawn. Additional sensitivity analyses were also performed using more extreme assumptions about missing primary outcome data. For further details, see the Supplementary material.

Secondary outcomes were analysed by fitting two-level logistic regression models (proportion of young people at 24 months classing themselves as weekly smokers, reporting having ever used cannabis, having used cannabis in the previous 12 months, having used cannabis in the previous 30 days, and having experienced at least one of the listed alcohol-related problems in the previous 12 months) and two-level Cox proportional hazards models (time to initiation of alcohol, smoking, and drugs – in those participants who had not initiated at baseline). Covariate adjustment for secondary analyses mirrored that used for the primary analyses.

Potential modifiers of the intervention with respect to both primary outcomes were investigated by fitting interaction terms to trial arm and pre-planned subgroups (age, gender, and smoking behaviour of the young person, drinking behaviour of the parents/carers, socioeconomic status assessed using the Family Affluence Scale (low, medium or high) and highest occupational status of parents/carers (categories based on the NS-SEC self-coded method), family categorisation (family likely to experience challenges in a group setting / family without challenges in a group setting), young person reported SDQ, domains of the Family Relationship Index, and averaged young person and parent/carer scores of General Child Management scale at baseline).

As it was one of the distinguishing features of this trial, further exploratory post-hoc subgroup analyses were conducted to investigate potential differential intervention effects by family categorisation for the smoking, cannabis use, and SDQ outcomes, the latter being included because of its focus on mental health, conduct problems, and peer influences.

All analyses were conducted using STATA version 13.0.

Role of funder
The study funder was not involved in the design of the study, or collection, analysis, or interpretation of the data. The corresponding author had access to all data in the study and had final responsibility for the decision to submit for publication.

Patient and Public Involvement
Members of the public were involved in refining and piloting of outcome measures and study procedures. This included a mix of families who had already received SFP10-14 (and who were able to comment on the perceived relevance of proposed outcomes) and those without any prior knowledge of the intervention (and were therefore able to provide input on how trial participants with no previous knowledge might perceive study materials). Piloting comprised asking participants to complete baseline interviews with researchers, and then provide feedback on clarity of written information, time taken to complete the baseline interview, and the acceptability and appropriateness of outcome measures. This information was then used to refine the final selection of outcome measures and format of information sheets, etc. Input was also sought from a young people's research advisory group (ALPHA), led by a public involvement officer embedded within the study team. The public involvement officer formed stakeholder groups – formed of trial participants, to advise the study team at key points on recruitment and retention strategies, maintaining contact with the trial cohort, and plans for disseminating trial findings.

Results
Participant recruitment took place between 8th February 2010 and 18th June 2012. Seven hundred and fifteen families were recruited into the trial. This comprised 918 parents/carers and 931 young people. The number of participants completing questionnaires at each assessment point is included in the trial profile (Figure 1). Primary outcome data was collected from young people. The primary analyses included 746 young people for the alcohol use outcome at two-years post-randomisation (80.1%) and 732 for the drunkenness outcome at two-years post-randomisation (78.6%). Completion rates were similar across trial arms. Analysis of levels of service utilisation data at 9, 15 and 24 month follow up did not identify any discernible differences between the intervention and control arms.

For peer review only - http://bmjopen.bmj.com/site/about/guidelines.xhtml

Baseline characteristics

Table 2 illustrates the characteristics of parents/carers and young people at baseline in control and intervention groups. Randomised groups were broadly similar at baseline, with small differences noted for young person gender and parent/carers' reported cigarette use.

Table 2: Baseline characteristics for Parent/carers and young people according to experimental group

Parent/carer baseline characteristics		Control (n=457)	SFP (Intervention) (n=461)
Age (Median, IQR)		37.0 (32.0 to 43.0)	37.0 (33.0 to 42.5)
Gender	Female	105 (23.0%)	103 (22.3%)
	Male	352 (77.0%)	358 (77.7%)
Ethnic group	White	451 (98.7%)	458 (99.6%)
	Non-white	6 (1.3%)	2 (0.4%)
Relationship status	Single, not in a relationship	54 (12.0%)	68 (14.8%)
	Single, in a relationship	97 (21.5%)	91 (19.8%)
	Married/ civil partnership	200 (44.3%)	183 (39.9%)
	Separated/ divorced	95 (21.1%)	113 (24.6%)
	Widowed	4 (0.9%)	3 (0.7%)
	Other	1 (0.2%)	1 (0.2%)
Ever smoked a cigarette	Never	231 (50.5%)	207 (44.9%)
	Yes, at least once	226 (49.5%)	254 (55.1%)
Ever drunk alcohol	Never	85 (18.6%)	90 (19.5%)
	Yes, at least once	372 (81.4%)	371 (80.5%)
AUDIT-C	Low risk	304 (66.5%)	294 (64.6%)
	High risk	153 (33.5%)	161 (35.4%)
Ever taken drug	Never	300 (65.6%)	307 (66.6%)
	Yes, at least once	157 (34.4%)	154 (33.4%)
Deprivation overall score (Median, IQR)		27.5 (18.5 to 41.2)	29.4 (18.0 to 44.5)
Young people baseline characteristics		Control (n=454)	SFP (n=477)
	Age (Median, IQR)	11.5 (10.0 to 13.0)	12.0 (11.0 to 13.0)
Gender	Female	231 (50.9%)	269 (56.4%)
	Male	223 (49.1%)	208 (43.6%)
Ethnic group	White	446 (98.5%)	474 (99.6%)
	Non-white	7 (1.5%)	2 (0.4%)
Family affluence scale	Low (0-2)	83 (18.4%)	99 (20.9%)
	Medium (3-5)	234 (52.0%)	231 (48.8%)
	High (6-9)	133 (29.6%)	143 (30.2%)
Ever smoked a cigarette	Never	330 (72.7%)	348 (73.0%)
	Yes, at least once	124 (27.3%)	129 (27.0%)
Ever drunk alcohol	Never	314 (69.3%)	326 (68.5%)
	Yes, at least once	139 (30.7%)	150 (31.5%)
Ever taken drug	Never	433 (95.4%)	450 (94.3%)
	Yes, at least once	21 (4.6%)	27 (5.7%)
Child-deprivation overall score (Median, IQR)		28.1 (19.7 to 39.2)	28.2 (18.3 to 39.6)

Primary analysis

Table 3 shows the results of alcohol use and drunkenness in the previous 30 days prior to their 24-month follow-up interview for young people. Young people allocated to the SFP group had higher alcohol use and drunkenness prevalence than those allocated to the control group (26.4% versus 24.6% for alcohol use, 10.2% versus 8.3% for drunkenness). However, results from the multilevel models indicated insufficient evidence to reject the null hypothesis of no difference between groups (alcohol use adjusted odds ratio 1.11 [95% CI 0.72 to 1.71], p = 0.646; drunkenness AOR = 1.46 [95% CI 0.83 to 2.55], p = 0.185). Conclusions were unaffected by pre-planned secondary analyses of the primary outcomes (see Supplementary material – tables S3-5).

For peer review only

Table 3: Primary alcohol use and drunkenness outcomes (24-month follow-up)

Outcome	ICC*	Proportion of events in control arm (%)	Proportion of events in SFP arm (%)	Adjusted odds ratio†	95% Confidence Interval		p-value
					Lower limit	Upper limit	
Alcohol use in previous 30 days‡	0.25	85/345 (24.6)	106/401 (26.4)	1.11	0.72	1.71	0.646
Drunkenness in previous 30 days§	0.18	28/338 (8.3)	40/394 (10.2)	1.46	0.83	2.55	0.185

**Intracluster correlation coefficient: proportion of the total variance (in whether or not a young person reports having used alcohol/been drunk in the 30 days prior to their 24-month follow-up interview) attributable to differences between families. Calculated using the standard $\pi^2/3$ estimator for binary outcomes. †Analyses adjusted for variables that were balanced on at randomisation (study area, family categorisation and average age of young people within the family) and baseline alcohol use / drunkenness (corresponding with the outcome). ‡Number of occasions in the last 30 days that participant has drunk alcohol (asked at 24-month follow-up) – dichotomised to 0/1 or more occasions. § Number of occasions in the last 30 days that participant has been drunk (asked at 24-month follow-up) – dichotomised to 0/1 or more occasions.*

Subgroup analysis for primary outcomes

Tables S6 and S7 (Supplementary material) provide the results of the planned subgroup analysis for alcohol use and drunkenness in the previous 30 days. There was no evidence of any statistically significant differential intervention effects for any of the subgroups for either primary outcome.

Other substance use secondary outcomes

There was no evidence of statistically significant differences between groups for the other substance use outcomes (Table 4). Rates of agreement between self-reported weekly smoking behaviour and saliva cotinine are show in Table S8 (supplementary material).

For peer review only

Table 4: Other substance use secondary outcomes (24 month follow-up)

Outcome		ICC*	Proportion of events in control arm (%)	Proportion of events in SFP arm (%)	Adjusted odds ratio	95% Confidence Interval		p-value
						Lower limit	Upper limit	
Alcohol-related problems in the last 12 months		0.18	51/326 (15.6)	60/384 (15.6)	0.95	0.59	1.54	0.849
Currently smoke at least one cigarette a week		0.41	52/347 (15.0)	59/402 (14.7)	0.94	0.53	1.66	0.828
Ever taken cannabis			46/338 (13.6)	58/393 (14.8)	1.21	0.57	2.55	0.617
Taken cannabis at least once in the last 12 months		0.43	41/337 (12.2)	49/393 (12.5)	1.01	0.51	1.97	0.985
Taken cannabis at least once in the last 30 days			20/338 (5.9)	21/393 (5.3)	0.77	0.36	1.68	0.518
Time to alcohol initiation†			88/246 (35.8)	81/273 (29.7)	0.86	0.64	1.17	0.344
Frequency of consuming 5+ drinks in a row in the last 30 days‡	0	0.00	315/345 (91.3)	348/401 (86.8)	1.55	0.95	2.51	0.080
	1 to 2		15/345 (4.3)	36/401 (9.0)				
	3 to 5		15/345 (4.8)	17/401 (4.2)				
Frequency of drinking beer‡	Never	0.00	290/346 (83.8)	331/401 (82.5)	1.15	0.77	1.72	0.492
	Rarely		20/346 (5.8)	17/401 (4.2)				
	Every month		26/346 (7.5)	36/401 (9.0)				
	At least once a week		10/346 (2.9)	17/401 (4.2)				
Frequency of drinking wine‡	Never	0.00	323/345 (93.6)	375/402 (93.3)	1.06	0.59	1.92	0.837
	Rarely		5/345 (1.4)	7/402 (1.7)				
	At least once a month		17/345 (4.9)	20/402 (5.0)				
Frequency of drinking spirits‡	Never	0.71	298/346 (86.1)	351/401 (87.5)	0.85	0.50	1.46	0.567
	Rarely		15/346 (4.3)	13/401 (3.2)				
	Every month		21/346 (6.1)	24/401 (6.0)				
	At least once a week		12/346 (3.5)	13/401 (3.2)				
Frequency of drinking alcopops‡	Never	0.35	303/346 (87.6)	345/402 (85.8)	1.24	0.78	1.97	0.361
	Rarely		19/346 (5.5)	17/402 (4.2)				
	At least once a month		24/346 (6.9)	40/402 (10.0)				
Frequency of drinking cider‡	Never	0.00	305/346 (88.2)	346/402 (86.1)	1.26	0.80	1.97	0.313
	Rarely		12/346 (3.5)	13/402 (3.2)				
	At least once a month		29/346 (8.4)	43/402 (10.7)				

**Calculated using the standard $\pi^2/3$ estimator for binary outcomes. †Single level Cox regression; Percentage of young people who initiated alcohol after baseline; adjusted hazard ratio. ‡Multilevel ordinal regression - not consuming 5+ drinks in a row and never drunk anything alcoholic were the reference categories.*

For peer review only

For peer review only - http://bmjopen.bmj.com/site/about/guidelines.xhtml

GCSE Performance at age 15/16
Based on the analysis of the primary and secondary self-reported outcomes at 2-year follow-up, it was determined that collection of the GCSE data was not justified.

Post-hoc subgroup analysis
Table 5 provides the results of unplanned subgroup analysis aiming to explore differential intervention effects on the secondary substance use outcomes and SDQ score by whether or not the family was likely to experience/present challenges in a group setting. The motivation for this exploratory work stemmed from the subgroup findings for the two primary outcomes, which demonstrated small (not statistically significant) differential effects favouring families experiencing/presenting challenges within a group setting. The attrition rate for the 24-month data was similar between families with or without challenges in a group setting (17.1% for families with challenges versus 12.2% without). The pattern found for the primary outcomes (i.e. differential outcomes favouring families with challenges) was reflected across the additional outcomes investigated. Taking the 'cannabis' outcome as one example, we see that the odds of reporting cannabis use in the last 12 months are higher for the main effect (i.e. higher for those in a family with challenges and allocated to the control arm, compared to families without challenges in a group setting, allocated to the control arm), and the interaction term indicates a reversal of this effect for those in the SFP arm (i.e. cannabis use is higher for families without challenges allocated to the SFP arm than families with challenges allocated to the SFP arm).

Table 5: Post-hoc subgroup analyses for the secondary substance use outcomes and SDQ

Subgroup	Main effect adjusted odds ratio*	95% Confidence Interval		Subgroup x SFP interaction adjusted odds ratio	95% Confidence Interval	
		Lower limit	Upper limit		Lower limit	Upper limit
Currently smoke at least one cigarette a week	1.95	0.83	4.55	0.35	0.11	1.10
Ever taken cannabis	2.14	0.70	6.58	0.33	0.07	1.50
Taken cannabis at least once in the last 12 months	2.11	0.79	5.68	0.25	0.06	0.99
Taken cannabis at least once in the last 30 days	1.22	0.39	3.77	0.30	0.06	1.54
Subgroup	Adjusted mean difference	95% Confidence Interval		Subgroup x SFP interaction adjusted mean difference	95% Confidence Interval	
		Lower limit	Upper limit		Lower limit	Upper limit
Strengths and difficulties total difficulties score	1.77	0.61	2.93	-2.27	-3.83	-0.71

*The main effect can be interpreted as the effect of belonging to a family with challenges in a group setting (relative to a family without challenges in a group setting) for those allocated to the control arm.

Cost-consequences analysis results
The costs associated with the implementation of the intervention were £1,163,278, which equated to £20,773 per programme and £1,240 per participant, with wide variation in costs across programme areas. There was no difference in costs of healthcare utilisation between the groups at 9-, 15- and 24-month follow-up. At 9- and 15-month follow-up the main cost driver was that of primary care usage, while at 24-month follow-up the main driver was in-patient episodes. There was no difference between the groups for adult EQ-5D scores at baseline or 24 –month follow-up.

EQ-5D scores for adults at both baseline and 24 –month follow-up were very similar between the intervention and control arms. Conversely, the EQ-5D scores for the children in the intervention arm saw a difference in the scores between baseline and 24 –month follow-up (a mean increase of 0.043 per participant in the intervention arms, compared to 0.030 in the control).

For peer review only - http://bmjopen.bmj.com/site/about/guidelines.xhtml

A complete case analysis was conducted looking at the number of parents who answered the 9-, 15- and 24-month questionnaires and who also answered both the baseline and 24-month EQ-5D questionnaire (Intervention n=197; Control n=129). The mean EQ-5D difference in the control group was 0.053 at a cost difference of £6,271, compared to 0.007 and £7,175 in the intervention group. This resulted in a difference of -0.046 at a cost of £904 with an incremental cost effectiveness ratio (ICER) of -£19,652. Therefore the intervention arm was dominated.

As with the vast majority of outcomes, there was no difference in resource utilisation or associated costs between the trial arms at 9-, 15- and 24-month follow-up. This was the case for resource utilisation in general and specifically to family/parenting-based support services.

Process evaluation
Detailed findings from the process evaluation have been reported.[25] Of the 56 programmes run, 47 (84%) achieved the target group size (5-12 families) at enrolment. Facilitators rated participant engagement high in 94% of activities. There was no evidence that engagement differed by group size. Twenty-two (39%) of the 56 programmes achieved the target composition of families with challenges (30%) and those without challenges in a group setting (70%). However, most of the remaining programmes (39/56) achieved a mix of families which was in line with the intended group composition model (i.e. the majority of families were those without challenges in a group setting).

Overall implementation fidelity was good. Facilitators were asked to rate fidelity of the sessions they delivered using a checklist designed by the SFP10-14 developers. Data were available for 50 of 56 programmes, across which facilitators rated 96% of individual activities as mostly/fully covered – this varied across trial sites from 90-99%. To assess reliability of facilitator reports a sample of 47 sessions from the 50 programmes for which data were available were observed and scored by researcher observers using the same fidelity assessment sheet. Observers rated 77% of observed activities as mostly or fully covered (range 63-88%): ICC scores from 2 observers = 0.73 (95% CI=0.65-0.79). Facilitators' and observers' scores agreed 83% of the time (area range 73-93%).

Recruitment of families was a key implementation challenge. Practitioners reported that the Proportionate Universal approach heightened such challenges as the universal recruitment diverged from the focus of existing systems and referring agencies on families with higher level needs. However, facilitators identified the Proportionate Universal approach to group composition (with a mix of families with and without challenges within a group setting) as helpful in enabling activities to be delivered as intended, the cultivation of positive group dynamics, and the provision of support to participants with additional needs.

Adverse outcomes
There were no SAEs which were both related (resulting from administration of any of the research procedures) and unexpected.

Discussion
In this paper we have reported findings from a randomised controlled trial of the SFP10-14 following its adaptation for a UK context. We found no evidence of between-group differences 2 years past baseline for the primary outcomes (young people's alcohol consumption and drunkenness in the last 30 days) or for any of the other substance use outcomes. Similarly, there were no between-group differences in relation to young people's wellbeing, stress, and emotional and behavioural problems. Findings were unaltered for pre-planned subgroup analyses. Our study findings contrast with evaluations of SFP10-14 conducted in the USA in which long term effects on substance use outcomes have been detected[8 10]. However, the absence of intervention effects in the UK setting replicates those of more recent European trials in which adapted versions of SFP10-14 have been evaluated. In Sweden no effects on substance use or 'breaking behaviours' were detected[29] though significant adaptations made to the intervention may have undermined its hypothesized mechanisms of action[30]. Likewise, findings from the trial conducted in Poland showed no effects on the primary outcomes (substance use) or aspects of family functioning.[31] An RCT in Germany identified only limited evidence of effectiveness.[32] For young people, there were no differences in rates of substance use, except for tobacco initiation (but only among those followed up at 18 months).

Process evaluation findings indicated that SFP10-14UK was delivered with good fidelity (albeit facilitators' reports were biased towards overstating fidelity), suggesting that this trial was a fair assessment of SFP10-14 when delivered as intended. The Proportionate Universal approach adopted successfully recruited families with

For peer review only - http://bmjopen.bmj.com/site/about/guidelines.xhtml

differing levels of need (but without the potential stigmatisation generated by targeted interventions), and most groups achieved positive group dynamics in line with hypothesized behaviour change mechanisms.

Several strengths of this study should be noted. There were good participant retention rates and high rates of data completion across both trial arms at main follow-up. At 24-month follow-up there were good levels of agreement between self-report of smoking behaviour and cotinine levels in collected samples. This study was designed as a pragmatic trial in which recruitment, intervention delivery systems, and implementation were intended to replicate how the intervention was likely to be delivered outside a trial setting, thus maximising the applicability of our findings. We did not identify evidence of compensatory provision of services in the control arm, increasing our confidence that the study findings would have detected any intervention effects which were present.

In terms of limitations, by virtue of necessity, all outcomes (including the primary outcomes) were measured using self-report. However, self-report of smoking behaviours was validated using measures of salivary cotinine. The trial utilised multiple statistical tests given the large number of outcomes. All outcomes measured hypothesized impacts of SFP10-14UK and mapped on to the intervention's logic model. In line with similar previous studies of complex interventions[33], we pre-specified primary and secondary outcomes, and also pre-defined subgroup analysis, clearly indicating those subgroup-analyses which were post-hoc.

SFP10-14UK did not replicate the impacts on substance misuse prevention found in the American evaluations. There are a number of possible explanations for the null findings. The American trials tended to have longer-term follow-ups and impacts were sometimes stronger in the longer-term (e.g. over 5-10 years). However, the epidemiological trajectory of young people's drinking etc in the UK is earlier than in the US studies so we anticipated any effects to be apparent earlier. The intervention was delivered with good fidelity, and thus implementation failure is unlikely to explain the absence of impacts on hypothesized outcomes. Likewise, cultural adaptations made to SFP10-14 for delivery in the UK prior to this trial focused on aspects of language and presentation of videos, and the core content and functions of the intervention were retained. The intervention as evaluated in this trial is therefore comparable with the original version evaluated in the USA. Trials of SFP10-14 in the USA have been criticised for their approach to data analysis and presentation of outcomes, with some suggestion that these might have led to unfounded claims for intervention effectiveness. [11 12 34] In this trial we addressed these criticisms by pre-specifying trial outcomes (via a published protocol) and how these would be analysed (through a statistical analysis plan which was approved by an independent steering committee).

It is possible that 'usual care' (to which SFP10-14UK was added for the intervention group) may vary in important ways between the American context and the European settings into which the intervention has been introduced. We were not able to compare data on routine provision of health and welfare services across the current and previous trials. In the present study, SFP10-14UK was integrated into existing parenting, family support and substance misuse prevention provision. Such provision, and their take up by families, may have varied across settings in which trials of SFP10-14 have taken place. The relatively well developed suite of services which formed 'usual care' in the current study may have made it more difficult for the intervention to show significant effects.

Another possibility is that intervention content may have interacted in different ways with the context of families receiving it. Post-hoc subgroup analysis of substance use-related secondary outcomes appeared to show that for families with challenges in a group setting, differences between intervention and control group were in favour of the intervention group. For families without challenges in a group setting, this pattern was reversed. It is possible that the intervention activities better met the needs of those families identified as likely to experience or present challenges in a group setting, or that key learning had greater fit with their circumstances and was thus easier to adopt and integrate. Alternatively it may be that group dynamics and the interaction between participants played a role in shaping this patterning, with benefits for those with challenges to the detriment of those without. For example, Wiggins, et al. have previously noted that "some interventions targeting people at risk can expose participants to the influence of new peers who are more supportive of or more engaged in behaviours associated with risk, thereby spreading risk"[35]. However, no such differences according to families with/without challenges in a group setting were identified for our primary outcomes. Whilst some previous trials of SFP10-14 have identified potential patterning of intervention effects by sub groups according to baseline assessment of risk for later substance use[32 36 37], our findings here are not directly comparable, since we were concerned with experience/presentation of challenges within a group setting, and not general levels of support needs or risk of later substance use. Previous research on interventions which are targeted based on assessment of young people's risk behaviours has identified the potential for stigmatisation of

For peer review only - http://bmjopen.bmj.com/site/about/guidelines.xhtml

participants and generation of iatrogenic effects through reinforcement and normalisation of harmful norms.[15 16] The Proportionate Universal approach adopted by implementation of SFP10-14UK in this study appeared to succeed in reaching families with higher levels of need whilst avoiding the stigmatisation of participants encountered by targeted interventions. However, it is unclear whether the potential intervention effects for families with challenges and the potential harms for families without challenges may be driven by intervention 'fit', or at least derives partly from group dynamics and the ways in which participants interact.

This trial highlights the importance of assessing the effectiveness of interventions when they are adapted for and then implemented in new settings. Such assessment involves not only a-priori identifying whether an adapted intervention remains faithful to its original logic model, but also understanding how it interacts with the new context into which is introduced. This context includes both the existing provision of services, and the needs of the participants who receive it.

Author Contributions

JSe wrote the first draft of the paper with input from other authors and led the process evaluation.
DG led the design and conduct of statistical analysis and assisted with writing the first draft of the paper and revisions.
ML contributed to statistical analysis and the writing of the first draft of the paper and subsequent drafts
JH was a trial manager and collected and analysed data on recruitment and retention.
SM provided input on the design, conduct and analysis of data for the process evaluation.
DF advised on the design of the study, the selection of outcome measures and the interpretation of the results.
KH contributed to the overall design of the study the design of the trial's statistical analysis plan, and the analysis of trial data.
JSc provided input on data collection and analysis within the process evaluation.
CP designed and led the health economic evaluation.
ZR contributed to the design and conduct of statistical analysis.
HR conducted data collection and analysis for the process evaluation.
CH was a trial manager and contributed to data collection, collation of data on recruitment and retention.
LM was chief investigator and led the overall design and conduct of the study.

All authors contributors contributed to the writing of the paper.
Jeremy Segrott is responsible for the overall content as guarantor, accepts full responsibility for the work and the conduct of the study, had access to the data, and controlled the decision to publish. The corresponding author attests that all listed authors meet authorship criteria and that no others meeting the criteria have been omitted.

Competing interests

All authors have completed the ICMJE uniform disclosure form at www.icmje.org/coi_disclosure.pdf and declare financial support from the National Prevention Research Initiative (managed by the Medical Research Council), Welsh Government and Cardiff Strengthening Families Team. DF's institution has previously received financial support for the development of the SFP 10–14 UK programme materials from the alcohol industry; there are no other relationships or activities that could appear to have influenced the submitted work.

Ethics Approval

Ethical approval for the trial was given by the Research Ethics Committee for Wales (reference 09/MRE09/53).

Funding

Funding of £2.1 M from the National Prevention Research Initiative, managed by the Medical Research Council – award G0802128, included c.£650 k implementation costs. The NPRI funding partners are Alzheimer's Research Trust; Alzheimer's Society; Biotechnology and Biological Sciences Research Council; British Heart Foundation; Cancer Research UK; Chief Scientist Office, Scottish Government Health Directorate; Department of Health; Diabetes UK; Economic and Social Research Council; Engineering and Physical Sciences Research Council; Health & Social Care Research & Development Office for Northern Ireland; Medical Research Council; The Stroke Association; Welsh Government; and World Cancer Research Fund.

A representative from the study funders was a member of the trial's Independent Trial Steering Committee. The Welsh Government provided c.£675 k of partnership funding, to cover the cost of implementation in three trial areas, and the associated training and support provided by the Cardiff Strengthening Families Programme team. Further support from Welsh Government provided £208k to cover programme delivery in six trial sites from

For peer review only - http://bmjopen.bmj.com/site/about/guidelines.xhtml

August 2011-July 2012. The Cardiff Strengthening Families Programme team also provided financial support for programme delivery and trial recruitment in schools.

At the time of the study DECIPHer was a UKCRC Public Health Research Centre of Excellence. Funding from the British Heart Foundation, Cancer Research UK, Economic and Social Research Council (RES-590-28-0005), Medical Research Council, the Welsh Government and the Wellcome Trust (WT087640MA), under the auspices of the UK Clinical Research Collaboration, is gratefully acknowledged. DECIPHer funding has supported JSe and JH's input into the trial. The centre is now funded by Welsh Government through Health and Care Research Wales. DECIPHer funding has supported JSe and JH's input into the trial.

LM is supported by the Medical Research Council (MC_UU_12017/14) and Chief Scientist Office (SPHSU14).

The Centre for Trials Research is funded by Health & Care Research Wales and Cancer Research UK.

Acknowledgements
We would like to thank the parents/carers and young people who participated in the trial. Grateful thanks are also due to the delivery teams who implemented SFP10-14UK as part of the trial, the independent trial steering committee and data monitoring committee, and the Participant Resource Centre at Cardiff University.

Data sharing
Researchers can request access to de identified participant data by contacting the corresponding author. Requests for access to data will be subject to approval of a proposal, completion of a signed data access agreement, and compliance with institutional and legal requirements. The study protocol is freely available, and the statistical analysis plan is available upon request.

Transparency Declaration
The lead author (the manuscript's guarantor) affirms that the manuscript is an honest, accurate, and transparent account of the study being reported; that no important aspects of the study have been omitted; and that any discrepancies from the study as originally planned (and, if relevant, registered) have been explained.

Licence Statement
I Jeremy Segrott, the Submitting Author has the right to grant and does grant on behalf of all authors of the Work (as defined in the below author licence), an exclusive licence and/or a non-exclusive licence for contributions from authors who are: i) UK Crown employees; ii) where BMJ has agreed a CC-BY licence shall apply, and/or iii) in accordance with the terms applicable for US Federal Government officers or employees acting as part of their official duties; on a worldwide, perpetual, irrevocable, royalty-free basis to BMJ Publishing Group Ltd ("BMJ") its licensees and where the relevant Journal is co-owned by BMJ to the co-owners of the Journal, to publish the Work in BMJ Open and any other BMJ products and to exploit all rights, as set out in our licence.

The Submitting Author accepts and understands that any supply made under these terms is made by BMJ to the Submitting Author unless you are acting as an employee on behalf of your employer or a postgraduate student of an affiliated institution which is paying any applicable article publishing charge ("APC") for Open Access articles. Where the Submitting Author wishes to make the Work available on an Open Access basis (and intends to pay the relevant APC), the terms of reuse of such Open Access shall be governed by a Creative Commons licence – details of these licences and which Creative Commons licence will apply to this Work are set out in our licence referred to above.

List of figures
Figure 1: Trial consort participant flow chart.

For peer review only - http://bmjopen.bmj.com/site/about/guidelines.xhtml

References

1. Foxcroft DR, Lowe G. Adolescents' Alcohol use and Misuse: The socializing influence of perceived family life. *Drugs: Education, Prevention and Policy* 1997;4(3):215-29. doi: 10.3109/09687639709028544
2. Garmienė A, Žemaitienė N, Zaborskis A. Family time, parental behaviour model and the initiation of smoking and alcohol use by ten-year-old children: an epidemiological study in Kaunas, Lithuania. *BMC Public Health* 2006;6(1):287. doi: 10.1186/1471-2458-6-287
3. Velleman RDB, Templetonl LJ. Substance misuse by children and young people: the role of the family and implications for intervention and prevention. *Paediatrics and Child Health* 2007;17(1):25-30. doi: 10.1016/j.paed.2006.12.002
4. Oldfield J, Humphrey N, Hebron J. The role of parental and peer attachment relationships and school connectedness in predicting adolescent mental health outcomes. *Child and Adolescent Mental Health* 2016;21(1):21-29. doi: 10.1111/camh.12108
5. Cuijpers P. Three Decades of Drug Prevention Research. 2003;10(1):7-20. doi: 10.1080/0968763021000018900
6. Turner KMT, Sanders MR. Dissemination of evidence-based parenting and family support strategies: Learning from the Triple P—Positive Parenting Program system approach. *Aggression and Violent Behavior* 2006;11(2):176-93. doi: 10.1016/j.avb.2005.07.005
7. Molgaard VM, Spoth R, Redmond C. Competency training: The Strengthening Families Program for Parents and Youth 10 142000.
8. Spoth R, Redmond C, Shin C, et al. Brief Family Intervention Effects on Adolescent Substance Initiation: School-Level Growth Curve Analyses 6 Years Following Baseline. *Journal of Consulting and Clinical Psychology* 2004;72(3):535-42. doi: 10.1037/0022-006X.72.3.535
9. Spoth RL, Clair S, Shin C, et al. Long-term Effects of Universal Preventive Interventions on Methamphetamine Use Among Adolescents. *Archives of Pediatrics & Adolescent Medicine* 2006;160(9) doi: 10.1001/archpedi.160.9.876
10. Spoth RL, Trudeau LS, Guyll M, et al. Benefits of Universal Intervention Effects on a Youth Protective Shield 10 Years After Baseline. *Journal of Adolescent Health* 2012;50(4):414-17. doi: 10.1016/j.jadohealth.2011.06.010
11. Gorman DM. Letter to the Editor: Sources of bias and need for caution in interpreting the results of Spoth et al.'s (2017) PROSPER study. *Psychological Medicine* 2018;48(4):694-96. doi: 10.1017/s0033291717002355
12. Gorman DM. The decline effect in evaluations of the impact of the Strengthening Families Program for Youth 10-14 (SFP 10-14) on adolescent substance use. *Children and Youth Services Review* 2017;81:29-39. doi: 10.1016/j.childyouth.2017.07.009
13. Dryden R, Williams B, McCowan C, et al. What do we know about who does and does not attend general health checks? Findings from a narrative scoping review. *BMC Public Health* 2012;12(1):723. doi: 10.1186/1471-2458-12-723
14. Bayer JK, Hiscock H, Morton-Allen E, et al. Prevention of mental health problems: rationale for a universal approach. *Archives of Disease in Childhood* 2007;92(1):34-38.
15. Sorhaindo A, Bonell C, Fletcher A, et al. Being targeted: Young women's experience of being identified for a teenage pregnancy prevention programme. *Journal of Adolescence* 2016;49:181-90. doi: 10.1016/j.adolescence.2016.03.013
16. Dishion TJ, Tipsord JM. Peer Contagion in Child and Adolescent Social and Emotional Development. *Annual Review of Psychology* 2011;62(1):189-214. doi: 10.1146/annurev.psych.093008.100412

For peer review only - http://bmjopen.bmj.com/site/about/guidelines.xhtml

17. Segrott J. Recruitment and group composition strategies for family-based substance misuse prevention interventions: an exploratory evaluation. 2013;8(2):89-109. doi: 10.1108/jcs-03-2013-0007
18. Marmott Review. Marmot Review: Fair society, healthy lives: strategic review of health inequalities in England post 2010. London: Institute of Health Equity, 2010.
19. Marmot Review. Marmot Review: Fair society, healthy lives: strategic review of health inequalities in England post 2010. London: Institute of Health Equity, 2010.
20. UCL Institute of Health Equity. Review of social determinants and the health divide in the WHO European Region: final report. Copenhagen: WHO Regional Office for Europe, 2013.
21. Carey G, Crammond B, De Leeuw E. Towards health equity: a framework for the application of proportionate universalism. *International Journal for Equity in Health* 2015;14(1) doi: 10.1186/s12939-015-0207-6
22. Segrott J, Gillespie D, Holliday J, et al. Preventing substance misuse: study protocol for a randomised controlled trial of the Strengthening Families Programme 10–14 UK (SFP 10–14 UK). *BMC Public Health* 2014;14(1):49. doi: 10.1186/1471-2458-14-49
23. Molgaard VM, Spoth R, Redmond C. Competency training: the Strengthening Families Program for parents and youth 10–14. *OJJDP Juvenile Justice Bulletin* 2000;(NCJ 182208)
24. Allen D, Coombes L, Foxcroft DR. Cultural accommodation of the Strengthening Families Programme 10-14: UK Phase I study. *Health Education Research* 2006;22(4):547-60. doi: 10.1093/her/cyl122
25. Segrott J, Murphy S, Rothwell H, et al. An application of Extended Normalisation Process Theory in a randomised controlled trial of a complex social intervention: process evaluation of the Strengthening Families Programme (10-14) in Wales, UK. 2017 doi: 10.1016/j.ssmph.2017.01.002
26. Lyons RA, Jones KH, John G, et al. The SAIL databank: linking multiple health and social care datasets. *Bmc Medical Informatics and Decision Making* 2009;9 doi: 10.1186/1472-6947-9-3
27. Currie C, Zanotti C, Morgan A, et al. Social determinants of Health and Well-Being among Young People. Health Behaviour in School-Aged Children (HBSC) Study: International Report from the 2009/2010 Survey. Health Policy for Children and Adolescents. Copenhagen: WHO Regional Office for Europe, 2012.
28. Vansteelandt S, Goetghebeur E. Causal inference with generalized structural mean models. *Journal of the Royal Statistical Society: Series B (Statistical Methodology)* 2003;65(4):817-35. doi: 10.1046/j.1369-7412.2003.00417.x
29. Skarstrand E, Sundell K, Andreasson S. Evaluation of a Swedish version of the Strengthening Families Programme. *The European Journal of Public Health* 2014;24(4):578-84. doi: 10.1093/eurpub/ckt146
30. Segrott J, Holliday J, Rothwell H, et al. Cultural adaptation and intervention integrity: a response to Skarstrand, Sundell and Andreasson. *The European Journal of Public Health* 2014;24(3):354-55. doi: 10.1093/eurpub/cku039
31. Foxcroft DR, Callen H, Davies EL, et al. Effectiveness of the strengthening families programme 10–14 in Poland: cluster randomized controlled trial. *The European Journal of Public Health* 2016:ckw195. doi: 10.1093/eurpub/ckw195
32. Baldus C, Thomsen M, Sack PM, et al. Evaluation of a German version of the Strengthening Families Programme 10-14: a randomised controlled trial. *European journal of public health* 2016;26(6):953-59. doi: 10.1093/eurpub/ckw082 [published Online First: 2016/07/05]

For peer review only - http://bmjopen.bmj.com/site/about/guidelines.xhtml

33. Bonell C, Allen E, Warren E, et al. Effects of the Learning Together intervention on bullying and aggression in English secondary schools (INCLUSIVE): a cluster randomised controlled trial. *The Lancet* 2018;392(10163):2452-64. doi: 10.1016/s0140-6736(18)31782-3
34. Gorman DM. Flexible data analysis and evaluations of the SFP 10-14. *The European Journal of Public Health* 2015;25(5):900.2-01. doi: 10.1093/eurpub/ckv155
35. Wiggins M, Bonell C, Sawtell M, et al. Health outcomes of youth development programme in England: prospective matched comparison study. *BMJ* 2009;339(jul07 2):b2534-b34. doi: 10.1136/bmj.b2534
36. Spoth R, Trudeau L, Redmond C, et al. Replicating and extending a model of effects of universal preventive intervention during early adolescence on young adult substance misuse. *Journal of Consulting and Clinical Psychology* 2016;84(10):913-21. doi: 10.1037/ccp0000131
37. Spoth R, Trudeau L, Redmond C, et al. Replication RCT of Early Universal Prevention Effects on Young Adult Substance Misuse. *Journal of consulting and clinical psychology* 2014;82 doi: 10.1037/a0036840

For peer review only

Figure 1: Trial consort participant flow chart

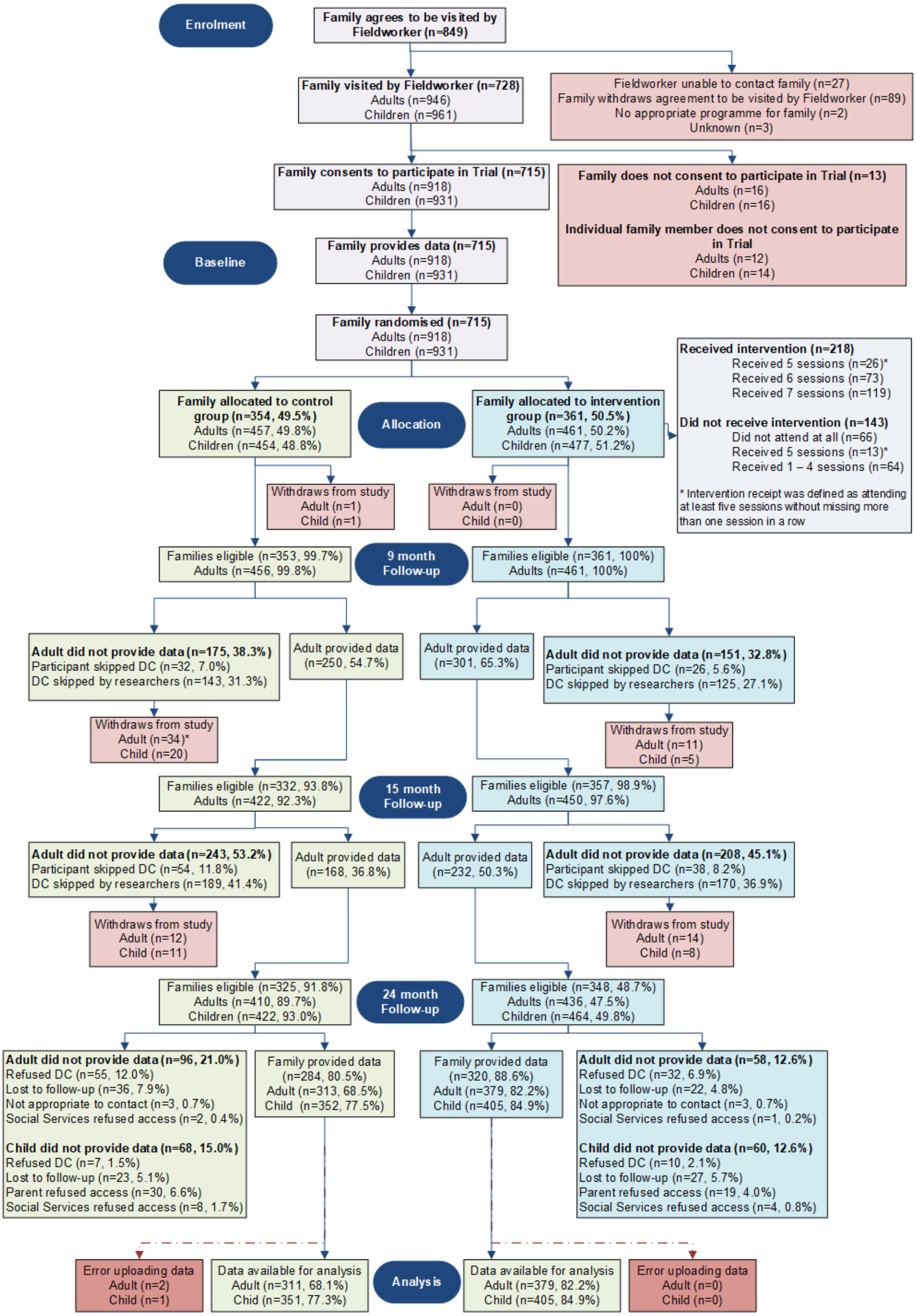

* 32 adults in the Control group withdrew at the 9 month data sweep. A further two adults withdrew prior to being scheduled for their 15 month data sweep

Supplementary material

Table S1: Measures collected at 24 month follow-up from young people

Domain/question topic	Purpose	Measure/Source
**Behaviour - Strengths and Difficulties**		
Strengths and difficulties / wellbeing and stress	TO	SDQ[1]
**Life at Home**		
Family activities (opportunities for involvement in pro-social activities; possibly bonding)	ST	From HBSC/ PEACH study
Young people's own time		From West of Scotland Twenty 07
Parenting/child management	IO/ TO	General Child Management Measure, Project Family[2-4]
Parents and school		Three questions; one adapted from the Child Rearing Practices Measure
Help around the home	IO	Adapted versions of questions asked to parents at 9/15 months.
Development of self-efficacy	ST/ TO	Bandura's Self Efficacy Scale[5]
Attachment to parents	ST	The Security Scale[6]
Befriending pro-social/anti-social peers	ST	Social Development Model scale on friends' anti-social qualities (Interaction with antisocial peers scale)[7]
Positive bonding to school	ST	School Bonding Measure / SDM[8]
**Participant substance use**		
Smoking behaviour – ever smoked?		Question from Substance Initiation Index[9]
Smoking status	TO/ SO	ASSIST study version of NatCen/NFER question[10]
Age first smoked	TO	From HBSC questionnaire
Ever drunk a drink?		NatCen/NFER[11]
Age of first drink	SO	Adapted from HBSC/NatCen/NFER
Drinking frequency, last month (including different types)	SO	Adapted questions from HBSC questionnaire
Ever really drunk?		From HBSC questionnaire
Age first drunk		Adapted version of question in HBSC questionnaire
Number of times drunk, drinking alcohol or 5+ drinks in a row in last month	PO/SO	Amended from HBSC questionnaire
Alcohol-related problems	SO	Q21 from ESPAD survey[12]
Drug use – ever used?		Amended from HBSC/NatCen/NFER[11]
Cannabis use (ever, 12 months, or 30 days)	SO	From HBSC questionnaire
Age of first drug use	TO	Adapted from HBSC questionnaire
**General health**		
Health state today	HE/TO	EQ-5D (child version)[13]
General health	HE/TO	UK Kidscreen 10[14]

For peer review only

Table S2: Measures collected at 24 month follow-up from parents/carers

Domain/question topic	Purpose	Measure/Source
**Life at home**		
Family activities		From HBSC/11-16 West of Scotland adult questionnaire
Family functioning	TO	Family relationship index[15,16]
Parenting/child management	SO, IO	General child management measure, Project Family[2-4]
Parent–child bonding	ST	Adapted from Spoth's rural urban cumulative risk index and Arthur, et al.[17,18]
Parents and school		Question developed by project team and one from Conger's child rearing practices measure
**Participant substance use**		
Smoking behaviour		Heaviness of smoking index[19]
Alcohol use		Adapted AUDIT-C questions, as used by Pre-empt study[20]
Drug use		Adapted from HBSC/NatCen/ NFER[11]
**Health**		
Health status	HE, TO	GHQ[21,22]
Health	HE	1 item from SF-36[23]
Health state today	HE, TO	EQ-5D[13]
**Service utilisation**		
Health, social care, education and criminal justice service utilisation	HE	Modified from ALSPAC

Secondary analyses of the primary outcome

After adjusting alcohol use outcome for gender of young people, time from randomisation to 24 month, and intervention receipt, the results from multilevel modelling indicated that none of the adjustment had altered the results that drawn from the primary analysis (Table 8). Although the alcohol use outcome measure had been restructured into an ordinary outcome measure, the results from ordinal regression model provided a corresponded proportional odds ratio of 1.09 (0.70-1.71) with adjustments and proportional odds ratio of 1.11 (0.73-1.67) without adjustments, which suggests that there was no evidence of a between-group difference.

For peer review only

Table S3: Secondary analyses of the primary alcohol use outcome

Analysis	Odds ratio	95% Confidence Interval		p-value
		Lower limit	Upper limit	
Without any adjustments	1.11	0.74	1.66	0.626
Adjusting for gender of young person	1.11	0.72	1.72	0.641
Adjusting for time from randomisation to 24-month follow-up	1.08	0.70	1.68	0.725
Adjusting for intervention receipt (binary definition)	1.21	0.79	1.86	0.384
Adjusting for intervention receipt (number of weeks)	1.03	0.97	1.09	0.382
Analysis	Proportional odds ratio	95% Confidence Interval		p-value
		Lower limit	Upper limit	
As an ordinal outcome (adjusted)	1.09	0.70	1.71	0.689
As an ordinal outcome (without any adjustments)	1.11	0.73	1.67	0.637

In terms of drunkenness in the previous 30 days, results show that the model without controlling any adjustments reduced the treatment effect odds ratio from 1.46 to 1.27 but did not alter the conclusions that could be drawn from the analysis (Table 9). Similarly, model adjusted for gender for young person, time from randomisation to 24 month, and intervention receipt did not alter the conclusions, which suggests that there was no evidence of a between-group difference.

Table S4: Secondary analyses of the primary drunkenness outcome

Analysis	Odds ratio	95% Confidence Interval		p-value
		Lower limit	Upper limit	
Without any adjustments	1.27	0.73	2.21	0.392
Adjusting for gender of young person	1.48	0.84	2.58	0.173
Adjusting for time from randomisation to 24-month follow-up	1.35	0.77	2.37	0.298
Adjusting for intervention receipt (binary definition)	1.50	0.80	2.79	0.203
Adjusting for intervention receipt (number of weeks)	1.06	0.97	1.16	0.196
Analysis	Proportional odds ratio	95% Confidence Interval		p-value
		Lower limit	Upper limit	
As an ordinal outcome (adjusted)	1.42	0.82	2.46	0.211
As an ordinal outcome (without any adjustments)	1.26	0.71	2.25	0.429

For peer review only

Multiple imputation for primary outcomes

We randomised 931 young people within 715 families in total. For our two primary outcomes, this represents our full ITT population. We conducted sensitivity analysis in order to estimate the full ITT effect.

We had two participants who did not respond to the baseline alcohol questions and assumed that neither had consumed alcohol within the past 30 days when asked at baseline.

We imputed primary outcomes using a single-level imputation model which contained variables included in the analysis model and whether the young person had ever smoked (as reported at baseline). We imputed 20 datasets and used augmented regression to avoid perfect prediction.

In the table below, we present estimates from the primary analyses (which adjusts for multiple young people within families), estimates of a single-level version of the primary analyses, and single-level imputed estimates.

Table S5: Primary analyses accounting for missing data using multiple imputation

Outcome	Model	OR	95% CI	p-value
Alcohol use at 24-months	Primary	1.11	0.72 to 1.71	0.646
	Single-level primary	1.11	0.77 to 1.61	0.574
	Single-level imputed	1.11	0.75 to 1.63	0.612
Drunkenness at 24-months	Primary	1.46	0.83 to 2.55	0.185
	Single-level primary	1.46	0.83 to 2.55	0.185
	Single-level imputed	1.48	0.85 to 2.56	0.162

Primary outcomes – subgroup analysis

An association was found between age of young person at baseline and reported alcohol use at 24-month follow-up (1.82 [1.23-2.71]), but there was no evidence of any differential effect by trial arms (1.30 [0.85-1.98]). In terms of gender of young person, the odds ratios for main effects and subgroup interaction terms suggested that the intervention worked differently for males and females. However, both confidence intervals included one, which suggests that there was no evidence of a differential treatment effect by gender of young person. Additionally, the main effect of young person who reported smoked at baseline (2.08 [1.06-4.11]) and having at least one parent/carer categorised at high-risk from problematic drinking (1.93 [1.01-3.69]) were significantly associated with the alcohol use outcome, but there were no discernible differences between arms. Other planned subgroup analyses provided no evidence of the SFP group having a differential effect.

An association was found between young person who reported smoked at baseline and reported drunkenness at 24-month follow-up (5.73 [2.07-15.90]), but there was no evidence of any differential effect by trial arms (0.57 [0.17-1.94]). In terms of the average young person GCM score (General Child Management), a one-point increase on the GCM score was associated with a 59% decrease in the odds of being classed as a young person who had been drunk in the last 30 days (0.41 [0.18-0.96]) a (higher GCM score indicates greater levels of parent management). However, there was no evidence of any differential effect by trial arm (1.27 [0.45-3.56]). For gender of young person, the odds ratio for main effect showed a 17% increase for females who reported having been drunk in the previous 30 days at 24-month follow up compared to males, and the subgroup interaction indicated an 8% increase for females allocated to the SFP group compared to males and those allocated to the control group. However, both confidence intervals included one, which suggests that there was no evidence of a differential treatment effect by gender of young person. Overall, none of the subgroup analyses for the primary drunkenness outcome proved a significant association, which suggests that there was no evidence of a differential treatment effect.

For peer review only

Table S6: Subgroup analyses for the primary alcohol use outcome

Subgroup	Main effect odds ratio	95% Confidence Interval		p-value	Subgroup x SFP interaction	95% Confidence Interval		p-value
		Lower limit	Upper limit			Lower limit	Upper limit	
Age of young person at baseline	1.82	1.23	2.71	0.003	1.30	0.85	1.98	0.228
Gender of young person (female)	0.85	0.44	1.57	0.574	1.48	0.65	3.57	0.337
Young person had smoked at baseline	2.08	1.06	4.11	0.034	0.79	0.32	1.91	0.596
Having at least one parent/carer who is at high risk from problematic drinking	1.93	1.01	3.69	0.048	0.79	0.33	1.87	0.593
Baseline Family Affluence Scale (Medium)	1.13	0.44	2.86	0.750	1.08	0.31	3.73	0.589
Baseline Family Affluence Scale (High)	1.09	0.39	3.00		1.75	0.46	6.70	
Occupation Status (At least one parent in the family in part-time (but none in full-time) employment)	0.60	0.24	1.53	0.448	0.86	0.24	3.09	0.720
At least one parent in the family in full-time employment	0.91	0.41	2.02		1.46	0.50	4.25	
Young person belonging to a “family with challenges”	0.99	0.52	1.91	0.984	0.51	0.21	1.24	0.140
Strengths and difficulties score at baseline	1.02	0.97	1.07	0.498	1.00	0.93	1.07	0.929
Cohesion domain of the Family Relationship Index at baseline	1.01	0.99	1.03	0.328	0.99	0.96	1.01	0.273
Expressiveness domain of the Family Relationship Index at baseline	1.01	0.99	1.04	0.339	0.99	0.96	1.03	0.760
Conflict domain of the Family Relationship Index at baseline	1.02	0.99	1.05	0.211	1.00	0.97	1.04	0.871
Average Young person General Child Management score	1.01	0.54	1.91	0.968	0.45	0.18	1.12	0.086
Average Parent/carer General Child Management score	1.54	0.76	3.15	0.233	0.67	0.28	1.64	0.383

For peer review only

Table S7: Subgroup analyses for the primary drunkenness outcome

Subgroup	Main effect odds ratio	95% Confidence Interval		p-value	Subgroup x SFP interaction	95% Confidence Interval		p-value
		Lower limit	Upper limit			Lower limit	Upper limit	
Age of young person at baseline	2.22	0.98	5.04	0.056	1.30	0.67	2.53	0.435
Gender of young person (female)	1.17	0.49	2.80	0.690	1.08	0.35	3.29	0.920
Young person had smoked at baseline	5.73	2.07	15.90	0.001	0.57	0.17	1.94	0.369
Having at least one parent/carer who is at high risk from problematic drinking	1.40	0.58	3.38	0.448	1.68	0.53	5.26	0.376
Baseline Family Affluence Scale (Medium)	1.26	0.36	4.43	0.336	2.57	0.46	14.45	0.563
Baseline Family Affluence Scale (High)	0.99	0.25	3.88		2.18	0.34	14.15	
Occupation Status (At least one parent in the family in part-time (but none in full-time) employment)	1.31	0.46	3.73	0.673	0.76	0.17	3.33	0.550
At least one parent in the family in full-time employment	0.76	0.25	2.30		1.87	0.47	7.43	
Young person belonging to a "family with challenges"	1.19	0.50	2.85	0.692	0.41	0.13	1.30	0.131
Strengths and difficulties score at baseline	0.97	0.90	1.04	0.366	1.03	0.94	1.13	0.570
Cohesion domain of the Family Relationship Index at baseline	1.01	0.98	1.03	0.578	0.99	0.96	1.03	0.730
Expressiveness domain of the Family Relationship Index at baseline	0.99	0.96	1.02	0.516	1.01	0.97	1.05	0.661
Conflict domain of the Family Relationship Index at baseline	1.00	0.97	1.04	0.791	0.99	0.94	1.03	0.584
Average Young person General Child Management score	0.41	0.18	0.96	0.040	1.27	0.45	3.56	0.649
Average Parent/carer General Child Management score	1.13	0.43	2.98	0.804	0.77	0.24	2.46	0.656

Weekly smoking validated by salivary cotinine measures

Overall, 500 young people (53.7% of those randomised, 66.1% of those who provided 24-month follow-up interview data) provided saliva cotinine samples and self-reported weekly smoking behaviour at the 24-month follow-up interview. To identify weekly smokers using saliva cotinine, a cut-off of 15ng/mL was used[10,24]. Comparing cotinine samples with self-reported weekly smoking, 467 young people provided self-reported smoking data that agreed with their cotinine samples (93.4%). A slightly higher percentage of young people in

For peer review only

the SFP group reported being a non-weekly smoker and had cotinine samples ≤ 15 ng/mL and less than those who reported that they were a weekly smoker but had cotinine levels of ≤ 15 ng/mL.

Table S8: Agreement between self-reported weekly smoking behaviour and saliva cotinine

Self-report and saliva cotinine agreement		Control		SFP		Total	
		n	%	n	%	n	%
Concordant	Agree non-weekly smoker	176	78.6	227	82.2	403	80.6
	Agree weekly smoker	30	13.4	34	12.3	64	12.8
Discordant	Self-reported non-weekly smoker and cotinine > 15 ng/mL	13	5.8	9	3.3	22	4.4
	Self-reported weekly smoker and cotinine ≤ 15 ng/mL	5	2.2	6	2.2	11	2.2
Total		224	100.0	276	100.0	500	100.0

For peer review only

References

1. Goodman R. The Strengths and Difficulties Questionnaire: A Research Note. *Journal of Child Psychology and Psychiatry* 1997; **38**(5): 581-6.

2. McMahon R, Metzler C. Selecting parenting measures for assessing family based prevention interventions. In: Ashery R, Robertson E, Kumpfer K, Rockville M, eds. Drug abuse prevention through family interventions: National Institute on Drug Abuse; 1998: 294-323.

3. Spoth R, Redmond C, Haggerty K, Ward T. A Controlled Parenting Skills Outcome Study Examining Individual Difference and Attendance Effects. 1995; **57**(2): 449.

4. Spoth R, Redmond C, Shin C. Direct and indirect latent-variable parenting outcomes of two universal family-focused preventive interventions: extending a public health-oriented research base. *Journal of consulting and clinical psychology* 1998; **66**(2): 385-99.

5. Bandura A. A Guide for Constructing Self-Efficacy Scales. In: Pajares F, Urdan T, Greenwich, CT, eds. Self-Efficacy Beliefs of Adolescents: Information Age Publishing; 2006: 307-37.

6. Kerns K, Aspelmeier J, Gentzler A, Grabill C. Parent–child attachment and monitoring in middle childhood. *Journal of Family Psychology* 2001; **15**: 69-81.

7. Arthur M, Hawkins J, Catalano R, Pollard J. Student Survey of Risk and Protective Factors and Prevalence of Alcohol, Tobacco, & Other Drug Use. University of Washington: Social Development Research Group.

8. Hawkins JD, Guo J, Hill KG, Battin-Pearson S, Abbott RD. Long-Term Effects of the Seattle Social Development Intervention on School Bonding Trajectories. *Applied Developmental Science* 2001; **5**(4): 225-36.

9. Spoth RL, Redmond C, Trudeau L, Shin C. Longitudinal substance initiation outcomes for a universal preventive intervention combining family and school programs. *Psychology of Addictive Behaviors* 2002; **16**(2): 129-34.

10. Campbell R, Starkey F, Holliday J, et al. An informal school-based peer-led intervention for smoking prevention in adolescence (ASSIST): a cluster randomised trial. *The Lancet* 2008; **371**(9624): 1595-602.

11. National Centre for Social Research NFfE, Research. Smoking, Drinking and Drug Use Among Young People in England

in 2008 Full Report: NHS Information Centre for Health and Social Care, 2009.

12. Hibell B, Guttormsson U, Ahlström S, et al. The 2011 ESPAD Report: Substance Use Among Students in 36 European Countries: The Swedish Council for Information on Alcohol and other Drugs (CAN), 2011.

13. Rabin R, Charro FD. EQ-SD: a measure of health status from the EuroQol Group. *Annals of Medicine* 2001; **33**(5): 337-43.

14. The Kidscreen Group Europe. The KIDSCREEN Questionnaires: Quality of life questionnaires for children and adolescents: Handbook. Lengerich: Pabst Science Publishers, 2006.

15. Holahan CJ, Moos RH. Social support and adjustment: Predictive benefits of social climate indices. 1982; **10**(4): 403-15.

16. Billings AG, Moos RH. The role of coping responses and social resources in attenuating the stress of life events. *Journal of Behavioral Medicine* 1981; **4**(2): 139-57.

For peer review only

17. Spoth R, Goldberg C, Neppl T, Trudeau L, Ramisetty-Mikler S. Rural–urban differences in the distribution of parent-reported risk factors for substance use among young adolescents. *Journal of Substance Abuse* 2001; **13**(4): 609-23.
18. Arthur M, Hawkins J, Catalano R, Pollard J. Item-construct dictionary for the student survey of risk and protective factors and prevalence of alcohol, tobacco, and other drug use. Unpublished Technical Document. . Seattle, WA.: University of Washington, 1995.
19. Borland R, Yong HH, O'Connor RJ, Hyland A, Thompson ME. The reliability and predictive validity of the Heaviness of Smoking Index and its two components: Findings from the International Tobacco Control Four Country study. 2010; **12**(Supplement 1): S45-S50.
20. Bush K. The AUDIT Alcohol Consumption Questions (AUDIT-C)<subtitle>An Effective Brief Screening Test for Problem Drinking</subtitle>. *Archives of Internal Medicine* 1998; **158**(16): 1789.
21. Goldberg D. The Detection of Psychiatric Illness by Questionnaire. London: Oxford University Press; 1972.
22. Vieweg BW, Hedlund JL. The General Health Questionnaire (GHQ): A comprehensive review. *Journal of Operational Psychiatry* 1983; **14**(2): 74-81.
23. Ware JE, Sherbourne CD. The MOS 36-Item Short-Form Health Survey (SF-36). *Medical Care* 1992; **30**(6): 473-83.
24. Benowitz NL, Iii PJ, Ahijevych K, et al. Biochemical verification of tobacco use and cessation. *Nicotine & Tobacco Research* 2002; **4**(2): 149-59.

For peer review only

Checklist of items for reporting pragmatic trials

Section	Item	Standard CONSORT description	Extension for pragmatic trials	Location in the submitted paper
Title and abstract	1	How participants were allocated to interventions (eg, "random allocation," "randomised," or "randomly assigned")		p1 (title); p2 (abstract)
**Introduction**				
Background	2	Scientific background and explanation of rationale	Describe the health or health service problem that the intervention is intended to address and other interventions that may commonly be aimed at this problem	p4
**Methods**				
Participants	3	Eligibility criteria for participants; settings and locations where the data were collected	Eligibility criteria should be explicitly framed to show the degree to which they include	pp5-6

For peer review only

Section	Item	Standard CONSORT description	Extension for pragmatic trials	Location in the submitted paper
			typical participants and/or, where applicable, typical providers (eg, nurses), institutions (eg, hospitals), communities (or localities eg, towns) and settings of care (eg, different healthcare financing systems)	
Interventions	4	Precise details of the interventions intended for each group and how and when they were actually administered	Describe extra resources added to (or resources removed from) usual settings in order to implement intervention. Indicate if efforts were made to standardise the intervention or if the intervention and its delivery were allowed to vary between	P5, p13

For peer review only

Section	Item	Standard CONSORT description	Extension for pragmatic trials	Location in the submitted paper
			participants, practitioners, or study sites	
			Describe the comparator in similar detail to the intervention	p6
Objectives	5	Specific objectives and hypotheses		p5
Outcomes	6	Clearly defined primary and secondary outcome measures and, when applicable, any methods used to enhance the quality of measurements (eg, multiple observations, training of assessors)	Explain why the chosen outcomes and, when relevant, the length of follow-up are considered important to those who will use the results of the trial	Pp7-8

For peer review only

Section	Item	Standard CONSORT description	Extension for pragmatic trials	Location in the submitted paper
Sample size	7	How sample size was determined; explanation of any interim analyses and stopping rules when applicable	If calculated using the smallest difference considered important by the target decision maker audience (the minimally important difference) then report where this difference was obtained	p8
Randomisation—sequence generation	8	Method used to generate the random allocation sequence, including details of any restriction (eg, blocking, stratification)		p7
Randomisation—allocation concealment	9	Method used to implement the random allocation sequence (eg, numbered containers or central telephone), clarifying whether the sequence was concealed until interventions were assigned		p7

For peer review only

Section	Item	Standard CONSORT description	Extension for pragmatic trials	Location in the submitted paper
Randomisation—implementation	10	Who generated the allocation sequence, who enrolled participants, and who assigned participants to their groups		p7
Blinding (masking)	11	Whether participants, those administering the interventions, and those assessing the outcomes were blinded to group assignment	If blinding was not done, or was not possible, explain why	p7
Statistical methods	12	Statistical methods used to compare groups for primary outcomes; methods for additional analyses, such as subgroup analyses and adjusted analyses		pp8-9
**Results**				
Participant flow	13	Flow of participants through each stage (a diagram is strongly recommended)—specifically, for each group, report the numbers of participants randomly assigned,	The number of participants or units approached to take part in the trial, the number which were	Summarised on p9, with full details

For peer review only

Section	Item	Standard CONSORT description	Extension for pragmatic trials	Location in the submitted paper
		receiving intended treatment, completing the study protocol, and analysed for the primary outcome; describe deviations from planned study protocol, together with reasons	eligible, and reasons for non-participation should be reported	included in Figure 1
Recruitment	14	Dates defining the periods of recruitment and follow-up		p9
Baseline data	15	Baseline demographic and clinical characteristics of each group		p10
Numbers analysed	16	Number of participants (denominator) in each group included in each analysis and whether analysis was by "intention-to-treat"; state the results in absolute numbers when feasible (eg, 10/20, not 50%)		Number of participants included in analysis

For peer review only

Section	Item	Standard CONSORT description	Extension for pragmatic trials	Location in the submitted paper
				described on p42. Use of ITT described on p8.
Outcomes and estimation	17	For each primary and secondary outcome, a summary of results for each group and the estimated effect size and its precision (eg, 95% CI)		Pp10-13
Ancillary analyses	18	Address multiplicity by reporting any other analyses performed, including subgroup analyses and adjusted analyses, indicating which are prespecified and which are exploratory		Pp11-14

For peer review only

Section	Item	Standard CONSORT description	Extension for pragmatic trials	Location in the submitted paper
Adverse events	19	All important adverse events or side effects in each intervention group		p14
**Discussion**				
Interpretation	20	Interpretation of the results, taking into account study hypotheses, sources of potential bias or imprecision, and the dangers associated with multiplicity of analyses and outcomes		Pp14-16
Generalisability	21	Generalisability (external validity) of the trial findings	Describe key aspects of the setting which determined the trial results. Discuss possible differences in other settings where clinical traditions, health service organisation, staffing, or resources may vary from those of the trial	Pp14-16

For peer review only

Section	Item	Standard CONSORT description	Extension for pragmatic trials	Location in the submitted paper
Overall evidence	22	General interpretation of the results in the context of current evidence		Pp14-16

Cite as: *Zwarenstein M, Treweek S, Gagnier JJ, Altman DG, Tunis S, Haynes B, Oxman AD, Moher D for the CONSORT and Pragmatic Trials in Healthcare (Practihc) group. Improving the reporting of pragmatic trials: an extension of the CONSORT statement. BMJ 2008; 337;a2390.*

For peer review only
